# Spatially variant immune infiltration scoring in human cancer tissues

**DOI:** 10.1038/s41698-022-00305-4

**Published:** 2022-09-01

**Authors:** Mayar Allam, Thomas Hu, Jeongjin Lee, Jeffrey Aldrich, Sunil S. Badve, Yesim Gökmen-Polar, Manali Bhave, Suresh S. Ramalingam, Frank Schneider, Ahmet F. Coskun

**Affiliations:** 1 Wallace H. Coulter Department of Biomedical Engineering, Georgia Institute of Technology and Emory University, Atlanta, GA USA; 2 School of Electrical and Computer Engineering, Georgia Institute of Technology, Atlanta, GA USA; 3 Department of Hematology and Medical Oncology, Emory University School of Medicine, Atlanta, GA USA; 4 Winship Cancer Institute, Emory University, Atlanta, GA USA; 5 Department of Pathology and Laboratory Medicine, Emory University School of Medicine, Atlanta, GA USA; 6 Interdisciplinary Bioengineering Graduate Program, Georgia Institute of Technology, Atlanta, GA USA; 7 Parker H. Petit Institute for Bioengineering and Bioscience, Georgia Institute of Technology, Atlanta, GA USA

**Keywords:** Tumour immunology, High-throughput screening, Mathematics and computing

## Abstract

The Immunoscore is a method to quantify the immune cell infiltration within cancers to predict the disease prognosis. Previous immune profiling approaches relied on limited immune markers to establish patients’ tumor immunity. However, immune cells exhibit a higher-level complexity that is typically not obtained by the conventional immunohistochemistry methods. Herein, we present a spatially variant immune infiltration score, termed as SpatialVizScore, to quantify immune cells infiltration within lung tumor samples using multiplex protein imaging data. Imaging mass cytometry (IMC) was used to target 26 markers in tumors to identify stromal, immune, and cancer cell states within 26 human tissues from lung cancer patients. Unsupervised clustering methods dissected the spatial infiltration of cells in tissue using the high-dimensional analysis of 16 immune markers and other cancer and stroma enriched labels to profile alterations in the tumors’ immune infiltration patterns. Spatially resolved maps of distinct tumors determined the spatial proximity and neighborhoods of immune-cancer cell pairs. These SpatialVizScore maps provided a ranking of patients’ tumors consisting of immune inflamed, immune suppressed, and immune cold states, demonstrating the tumor’s immune continuum assigned to three distinct infiltration score ranges. Several inflammatory and suppressive immune markers were used to establish the cell-based scoring schemes at the single-cell and pixel-level, depicting the cellular spectra in diverse lung tissues. Thus, SpatialVizScore is an emerging quantitative method to deeply study tumor immunology in cancer tissues.

## Introduction

Lung cancer remains to be one of the leading causes of death worldwide, causing the loss of over 100,000 patients yearly. The therapeutic inefficacy is partly due to the disease heterogeneity, late diagnosis, and the aggressiveness of lung cancers. Both chemotherapies and radiation suffer from off-target effects, increasing the cytotoxicity and the adverse side effects for patients. Emerging immunotherapy approaches are utilizing the patient’s immune system in fighting their growing cancers to reduce the potential adverse effects of more conventional cancer therapies and to overcome drug resistance. However, these immune-based therapies work for a small subset of lung cancer patients and they suffer from spatial and temporal differential responses to treatments^1^. The main reason behind the poor success rates of immunotherapies is the lack of understanding of the molecular and cellular drivers of the tumor-driven immune changes between patients across different cancer stages and grades^2^.

The current established model to stratify patients’ tumors is based on the international American Joint Committee on Cancer/Union for International Cancer Control (AJCC/UICC) tumor-node-metastasis (TNM) classification system that completely lacks patients’ immune signatures. TNM is widely adopted in clinical practice to describe tumor progression and metastasis, and to predict patients’ prognosis^3^. On the other hand, patients with the same TNM classification exhibit vastly different responses to the same treatment because the TNM system fails to capture crucial biochemical, metabolic, and genetic signatures of patients’ tumors^4^. In prior reports, the degree of immune cell infiltration correlated with the prognosis of lung cancer patients, providing opportunities to classify immune and cancer cell spatial neighboring for predicting patients’ response to immunotherapies and improving their overall prognosis^5,6^.

Immunoscore classifies tumors based on immune cell infiltration and is used as a prognostic tool^7^. One of the early approaches for immunoscore was the manual or the digital numerical quantification of tumor-infiltrating CD3+ and CD8+ lymphocytes at the center and at the invasive margin of the tumors using patients’ whole slice tissue samples stained with immunohistochemistry (IHC) techniques. However, this approach is prone to fallacies due to the variability of staining protocols, results, and interpretations^3^. Several computational approaches complemented the IHC-based assays to quantify and visualize CD3+ and CD8+ tumor-infiltrating lymphocytes (TILs) including Visiopharm, HALO image analysis, or other custom packages^8–10^. Immunoscore approaches have also been extracted from publicly available databases, including the Cancer Genome Atlas (TCGA), to specifically examine the immune gene enrichments and their correlation with disease progression^11^. These prior immunoscore methods have relied mainly on a few subsets of immune cells including CD3+ and CD8+ lymphocytes due to the technical limitations of imaging datasets.

The spatial neighboring of a complex immune cell programming contributes to cancer prognosis including other phenotypes of T cells (e.g., CD4: Treg cells, Th1, Th2, Th17, and Tfh; CD8: Stem-like, exhausted, memory, and cytotoxic T cells), B cells, different phenotypes of macrophages (M1, and M2), mast cell, neutrophils, among others^5^. Thus, recent immunoscore approaches have started utilizing the rich molecular profiles generated by the multiplexed imaging techniques to visualize multiple immune markers in situ from the same tissue and apply spatial scoring methods to identify the different spatial infiltration patterns of immune cells distributions. Such an immunoscoring approach has recently identified the immunological regions in tumor architecture of diffuse large B-cell lymphoma (DLBCL)^12^. DLBCL exhibited spatial layering of immune infiltration patterns, akin to earth layers composed of an inner core, mantle, crust, and dispersed layers. The tumor core analysis demonstrated an immune desert with few immune cell infiltration, mainly CXCR3+ CD4+ cells, suggesting a new role for CXCR3 that grants access to CD4+ cells. The tumor mantle showed high infiltration of suppressive Treg cells, and exhausted CD8+ cells, causing an immune-suppressed layer. The outer edge of the tumor or the dispersed layer in the earth model presented highly infiltrated immune cells including the proliferating macrophages and CD8ɑ^12^. While this approach is promising, there is still an important need to visually quantify localized distributions of immune infiltrates in multiplexed tumor data. The presented study has established a spatially variant scoring approach to quantify the immune infiltration in tumor regions. Multiplexed imaging mass cytometry (IMC) with a metal isotope-tagged antibody panel of 26 markers was utilized to visualize the tumor-immune architecture and quantify the immune infiltration in lung cancers. These biospecimens get ionized from the surface of the stained tissue by an argon plasma and are then sent through a time-of-flight mass cytometer (CyTOF) through helium gas flow where they get analyzed based on their mass-to-charge ratio^13^.

A tumor microarray was annotated from the hematoxylin and eosin (H&E) stained tissues by an expert pathologist to study unique anatomical regions with varying cancer stages, grades, and immune infiltration patterns. In this pipeline, 16 immune markers were used to develop a spatially variant immunoscore tool for mapping the immune continuum of lung tumors, providing a more spatially resolved immune scoring solution compared to the prior approaches.

**Fig. 1:**
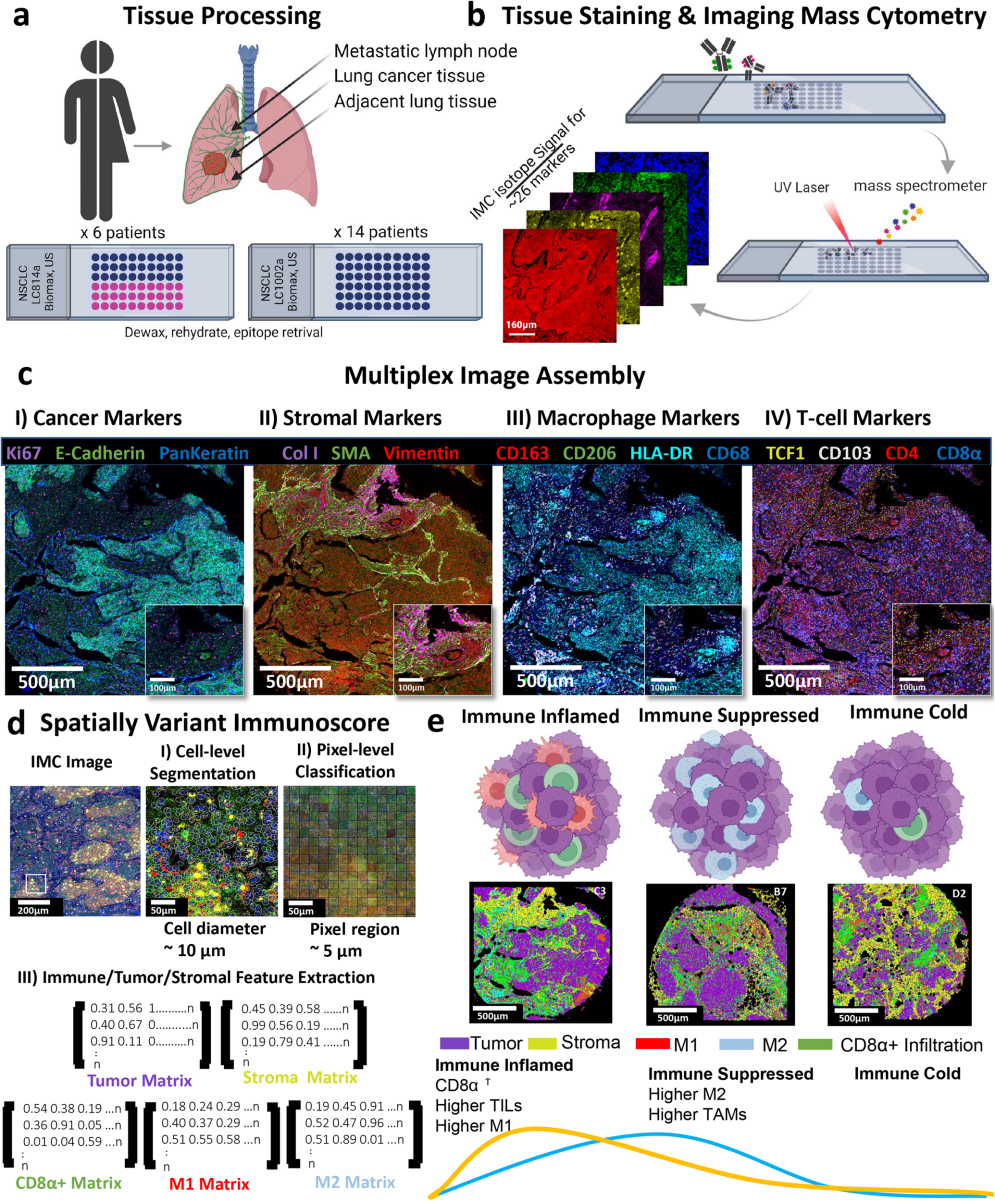
Imaging mass cytometry enables spatially resolved quantification of immune infiltrates in tumors using multiplexed images of human FFPE cancer tissues. **a** Tissue samples from 6 lung cancer patients and their matched 6 metastatic lymph nodes were chosen from a tissue array containing non-small lung cancers, and their matched metastatic lymph nodes (LC814a). Additional tissue samples from 14 lung cancer patients were chosen from a tissue array containing non-small lung cancer, cancer adjacent tissues, and normal tissue samples (LC1002a). **b** The tissue array was stained with a cocktail mix of 26 metal-tagged antibodies. Regions of interest (ROIs) with a size of 1500 µm x 1500-µm were then ablated using an Ultraviolet (UV) laser, and the ionized metal isotopes were analyzed based on their time-of-flight by a mass spectrometer. The imaging resolution was 1-µm. **c** Using the multiplexed antibody panel, unique tissue regions can be visualized by assigning pseudo-colors to isotope signals and their conjugated antibodies. Several tissue regions were identified on the same tissue section including cancer, stromal, and immune components to investigate the tumor microenvironment. **d** Cell-level segmentation and pixel-level classification were used to identify distinct tissue regions. Using the cell-level segmentation masks, the resulting average cell diameter was 10-µm. For the pixel-level classification, the pixel patch size was 5-µm, and the divided ROI area into 300 × 300 pixel patch squares or 90,000 total pixel patch squares. Separate arrays were generated to extract distinct tissue features including tumor regions using the expression data from E-Cadherin and PanKeratin, and stromal regions using the expression data from Smooth Muscle Actin, and Collagen Type 1. The immune cells infiltration was divided into CD8α+ cells for the inflammatory T-cells, CD68+HLA-DR+ for M1 macrophages, and CD68+CD206+CD163+ for M2 macrophages. **e** Immune cells distribution was found to follow a continuum, wherein “immune inflamed” cancers exhibited high infiltration of tumor-infiltrating lymphocytes (TILs) as well as M1 macrophages, “immune suppressed” cancers demonstrated high infiltration of M2 macrophages, and “immune cold” tumors with poor immune infiltration. Created with BioRender.com.

To develop a comprehensive understanding of the immune infiltration in lung tumors, we used CD8ɑ as a marker for cytotoxic T cells correlating with inflamed tumors and better disease prognosis^5^. In this panel, CD68 was chosen as a pan-macrophage marker corresponding with tumor-associated macrophages (TAMs). Since different phenotypes of TAMs were shown to have distinct roles in the disease development, CD163 and CD206 were targeted to identify the pro-tumor M2 phenotype and HLA-DR to identify the anti-tumor M1 phenotype^5,14^. These phenotypes were further supported with additional immune markers including PD-1, PD-L1, granzyme B, FoxP3, CD20, CD4, CD3, CD45RO, TCF1, CD103, and CD95 to investigate the complexity of immunity in the tumor microenvironment of the lung cancer patients’ tissues. Herein, we present a computational tool termed as SpatialVizScore using the multiplexed imaging data to visualize and quantify the immune infiltration patterns within lung tumors and their surrounding tumor microenvironment both at the single-cell and the pixel-level resolution (Fig. 1). The SpatialVizScore is emerging for multiplexed spatial data visualization, yielding the tumor’s immune infiltration continuum and discrete patient stratification to design personalized and precision immunotherapies.

## Results

The protein expression data for 26 markers were obtained from 12 tissues (Cohort 1) corresponding to six lung cancer patients along with their metastatic lymph nodes and an additional 14 lung cancer tissues (Cohort 2) used for validation (Fig. 1a, b and Supplementary Tables 1–2). Several tissue regions were then labeled based on the markers’ expression. Cancerous/paracancerous regions were labeled by the expression of pan-keratin and e-cadherin whereas stromal regions were marked by the expression of smooth muscle actin (SMA) and collagen type 1 (COL1) (Fig. 1c). E-cadherin is a transmembrane protein that is predominantly expressed by cells of epithelial origin and it functions as an adhesion protein. Similarly, pan-keratin is a widely known marker for epithelial cells and carcinomas. Therefore, these markers were used to identify cancer regions^15^. Besides, collagen I and SMA accumulation signifies extracellular matrix (ECM) alignment, remodeling, and fibrogenesis. Therefore, collagen I and SMA were used to define stromal regions on the patients’ samples^15^. Similarly, several immune markers were used to classify the various immune cell phenotypes (Fig. 1c). CD8ɑ was used as a marker for TILs and an inflamed state of the growing tumors. On the other hand, CD68 was used as a pan-macrophage marker along with CD163/CD206 for the pro-tumor M2 phenotype and HLA-DR for the anti-tumor M1 phenotype^5^. HLA-DR was used to profile M1-polarized macrophages as it functions to increase the antigen-presenting capacity of the differentiated monocytes which further leads to the production of the pro-inflammatory cytokines^16^. On the other hand, CD163 and CD206 were used to identify M2-polarized macrophages as they are macrophage scavengers or macrophage mannose receptors, respectively.

To extract the single-cell protein expression data, two single-cell segmentation techniques were evaluated including cell-level segmentation and pixel-level segmentation (Fig. 1d). These segmentation techniques were used to extract the labels’ expression and their spatial locations, which were then used in generating spatial proximity network maps to understand the infiltration pattern in patients’ tumors and their metastatic lymph nodes. The resulting spatial neighborhood networks were used to quantify patients’ immune infiltration in tumor and stromal regions and a final immunoscore was given to each patient tissue. The SpatialVizScore results showed a continuum of immune cell infiltration that ranged from immune cold tumors to immune suppressed tumors and to immune hot/inflamed tumors (Fig. 1e). This continuum was further analyzed with additional immune markers including FoxP3, PD-1, PD-Ll, granzyme B, CD3, CD4, CD45RO, CD20, CD95, CD103, and TCF1. (Fig. 1a–e).

The multiplexed and isotope-conjugated antibody panel of IMC was used to investigate the tissue composition (Supplementary Figs. 1–6). Since IMC ablates the tissue specimens after imaging, it was essential to acquire the H&E stained images of a sequential section of the tissues to validate cancer, stroma, and lymphocyte enriched regions (Supplementary Figs. 7–9). From both the H&E data and the visual IMC data, patients’ tumors were highly heterogeneous in the tumor and stromal regions and the infiltration of the immune cells (Supplementary Figs. 1–7). The resulting H&E images were verified by a pathologist against a third-party vendor’s information to identify the samples’ cancer type and grade and to validate the IMC data.

### Single-cell analysis for spatial infiltration scoring in the tumor microenvironment

**Fig. 2:**
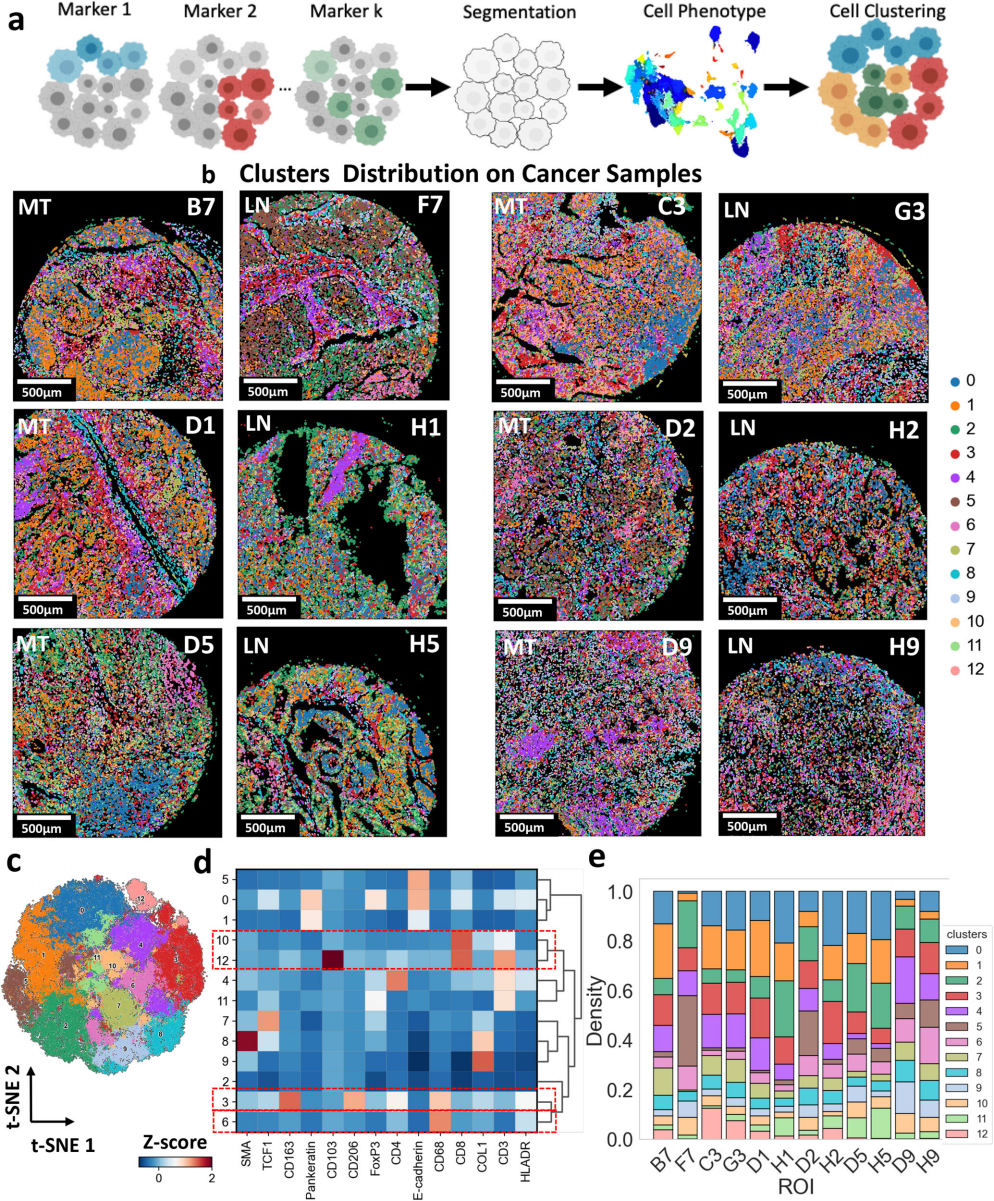
Cell-level segmentation and clustering showed the heterogeneity of markers’ expressions within the patient population in lung tumors. **a** Representative schematic shows the process of cell-level segmentation. Cell segmentation was performed using the deep-learning CellPose algorithm. The nuclei were segmented using signals from intercalators conjugated to ^191^Ir and ^193^Ir along with other markers associated with the nucleus including Histone 3, Ki67, and FoxP3. The cytosol was segmented by expanding the nuclei by 2 pixels. The number of cells *n* = 264,191. Created with BioRender.com. **b** The resulting data were clustered using a subset of markers including CD103, CD163, CD206, CD3, CD4, CD68, Collagen I, E-cadherin, HLA-DR, PanKeratin, SMA, TCF1, and Vimentin. The resulting clusters were visualized on the tissue samples by attributing each cell mask to the corresponding cluster color, indicating distinct separation of different tissue regions. MT represents metastatic tumor and LN indicates lymph nodes. **c** UMAP displayed the distribution and the separation of the resulting 13 clusters from the single-cell phenotypes showing the heterogeneity in markers’ expressions within the patient population. **d** Correlative heatmap provided the co-expression of markers across the 13 clusters that make up the dataset. **e** Marker abundance heatmap yielded the cluster makeup for all patients’ tissues.

Several multiplexed imaging modalities were developed to explore the complex architecture of the tumor microenvironment, including IMC^17^, multiplexed ion beam imaging (MIBI)^18^, cyclic immunofluorescence (CyCIF)^19^, and CO-Detection by indEXing (CODEX)^20^, among others^13^. The first step to deciphering the complexity in the tissue images is to segment and cluster individual cells to extract the expression levels of multiple markers in the multiplexed data at the single-cell level. Thereby, the SpatialVizScore pipeline starts with a two-step clustering approach. First, cell-level segmentation was performed on the tissue images using a deep-learning method via *Cellpose*^21^, and another cell mask identification technique via *CellProfiler*^22^. The segmented cells were assigned to phenotypes based on their marker expression levels. Second, a combinatorial graph-based clustering approach was used to cluster individual cells by their marker expression level (Fig. 2a and Supplementary Fig. 10). Representative patients’ tissue images were then reconstructed from the cell masks, and their clustering results by assigning each segmented cell to its corresponding cluster (Fig. 2b). Finally, each cluster was assigned a different color to visualize the clusters’ distribution and tumor microenvironment composition of the patients’ sample tissues.

We used unsupervised clustering with all the 26 markers included in the dataset which resulted in 13 distinct clusters (*i.e*., chosen to be optimum to identify major cell phenotypes) across all samples, identified by a t-distributed stochastic neighbor embedding (t-SNE) visualization and Leiden clustering analysis^23,24^ (Fig. 2c–e). Cluster 3 corresponded to M2-polarized macrophages with the markers CD68, CD163, and CD206. Further, Cluster 6 corresponded to M1-polarized macrophages with the marker expression CD68 and HLA-DR. Clusters 10 and 12 demonstrated the expression of CD3 and CD8ɑ, providing TILs. Cluster 12 yielded the expression of CD103 along with CD3 and CD8ɑ, corresponding to a specific subset of the TILs and the tissue-resident memory T-cells (Fig. 2d).

Besides the main immune clusters, cluster 0 showed the co-expression of pan-keratin and e-cadherin and cluster 8 showed the co-expression of collagen type I and SMA. Given the marker expression profiles of these two clusters, they corresponded to the tumor and stromal regions respectively. Clusters 3, 6, 10, and 12 indicated variant densities across the different primary tumor and metastatic lymph nodes, further necessitating more quantification of their infiltration patterns (Fig. 2e).

**Fig. 3:**
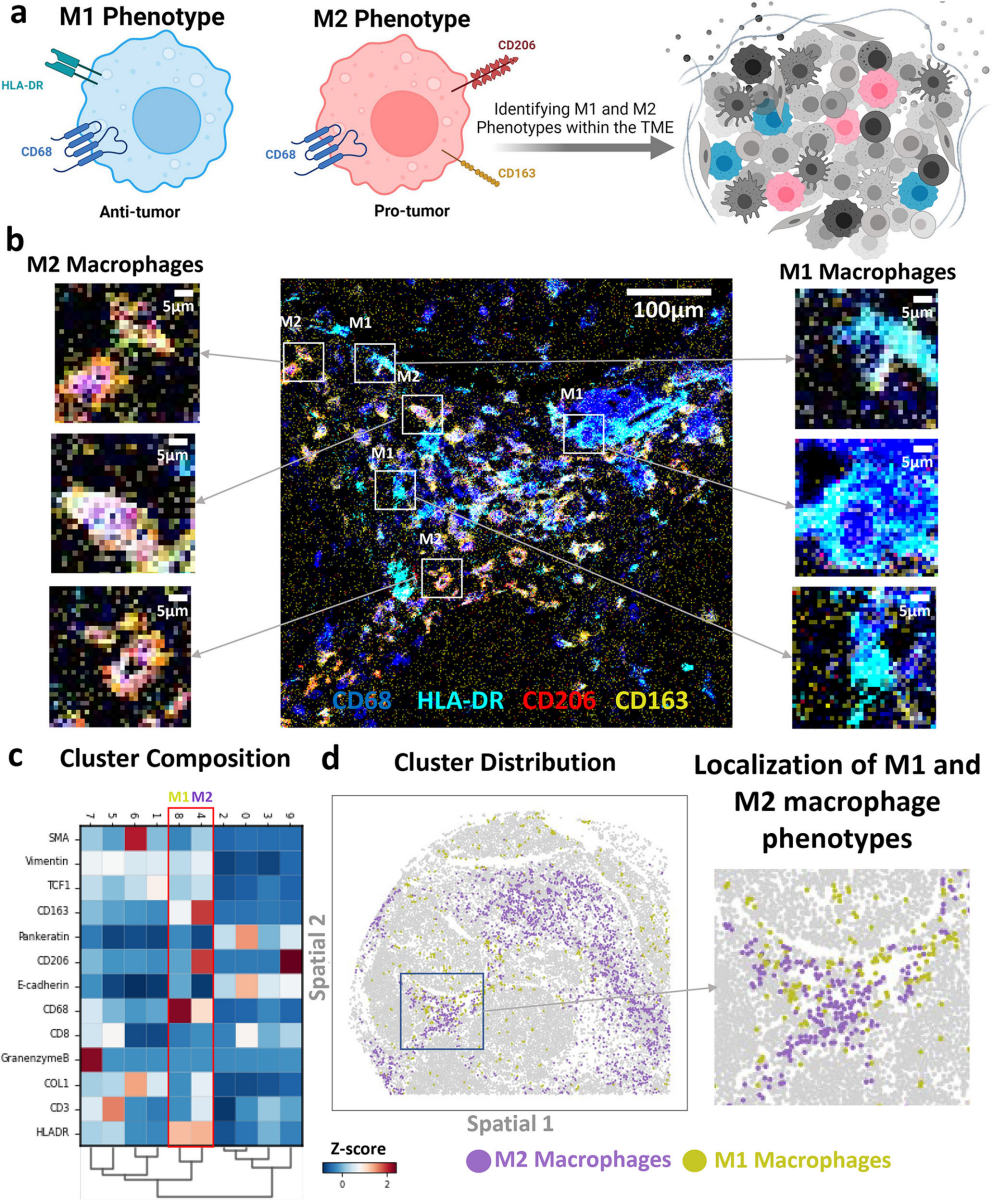
Identification of M1 and M2 macrophage phenotypes based on the expression of CD68, HLA-DR, CD163, and CD206 in the multiplexed IMC panel. **a** Representative schematic highlighting the polarization of macrophages into the anti-tumor M1 phenotype (CD68+HLA-DR+) and the pro-tumor M2 phenotype (CD68+CD163+CD206+). Both phenotypes occupy the tumor microenvironment and impact the overall disease prognosis. Created with BioRender.com. **b** Representative IMC images present the expression of CD68 in blue, HLA-DR in cyan, CD206 in red, and CD163 in yellow. CD68 and HLA-DR were chosen to identify M1-polarized macrophages whereas CD68, CD163, and CD206 were used to identify M2-polarized macrophages. **c** Correlation heatmap yielded the co-expression of markers across the different clusters. The macrophage M1 phenotype cluster showed the expression of CD68 and HLA-DR whereas the macrophage M2 phenotype showed the expression of CD68, CD163, and CD206. **d** Cluster distribution map demonstrated the interaction and localization between M1 and M2 clusters on an example cancer tissue image.

With this distinct separation of several phenotypes in the native tissue microenvironment, it came of special interest to quantify the infiltration of immune cell subsets within the different anatomical regions. M1 macrophages are known for their pro-inflammatory properties that lead to anti-tumor responses. However, M2 macrophages are known for their anti-inflammatory properties that lead to the development of a tolerogenic immune microenvironment that supports cancer progression (Fig. 3a, b). M1 and M2 were shown to be present in distinct clusters in lung cancer tissues, demonstrating the unique distributions of the macrophage markers (Fig. 3c). M1 and M2 clusters were found to be co-localized in some tissue regions or have unique infiltration patterns in other tissue regions (Fig. 3d). This allowed us to analyze the infiltration patterns of M1 and M2 macrophages in patients’ tumor samples.

To quantify the immune infiltration in cancer tissue samples, the tumor microenvironment was further classified into cancer-rich and stroma regions using the multiplex protein data. Patients’ tissue samples were reconstructed into tumor regions (with pan-keratin and e-cadherin markers expression), stromal regions (with collagen and smooth muscle action markers expression), and different subsets of immune cells. The CD8ɑ+ cells were utilized to identify the TILs. Moreover, CD68+CD163+CD206+ cells were marked to identify the M2-polarized macrophages, and CD68+HLA-DR+ cells were used to identify the M1-polarized macrophages (Supplementary Fig. 11).

**Fig. 4:**
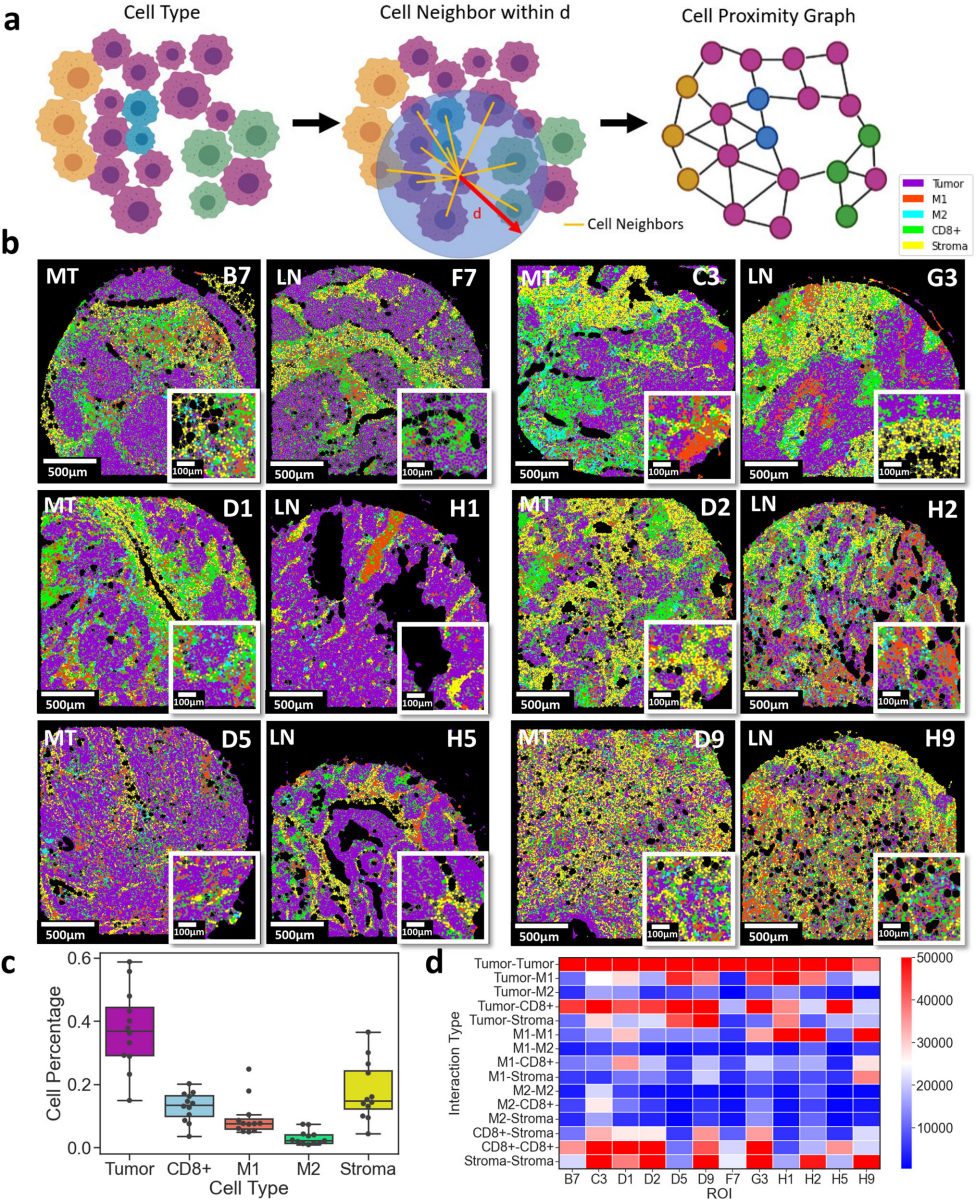
Tissue neighborhood analysis using single-cell data identifies the tumor, stromal, and immune cell relationships in lung cancer patients’ tissues. **a** Representative schematic demonstrates the process of neighborhood analysis. Each cell was assigned to a distinct type (tumor, stroma, CD8α, M1, and M2) based on the highest intensity values of markers. Tumor cells (T) were marked by the expression of pankeratin and e-cadherin, and stromal cells (S) were marked by the expression of collagen type 1 and SMA. CD8α+ cells were marked by the expression of CD8ɑ, M1 cells were marked by the expression of CD68 and HLA-DR, and M2 cells were marked by the expression of CD68, CD163, and CD206. A Cell network was then generated by connecting every cell centroid to its neighboring centroids within a 30-µm distance. Created with BioRender.com. **b** Cell network graphs on patients’ samples were presented. Magenta indicates tumor regions, yellow indicates stromal regions, green indicates CD8α cells, red indicates M1 cells, and cyan indicates M2 cells. **c** Box plot provided the anatomical composition of patients’ samples. Box plot demonstrated the distribution of the data with the minimum, first quartile (Q1), median, third quartile (Q3), and maximum. Original Data was overlaid on the boxplot. Tumor regions were the most predominant followed by stromal regions, CD8α+, M1, and lastly M2 cells (*N* = 12). **d** Heatmap summarized the distribution of neighborhood scores across different tissue regions.

Each patient sample was reconstructed based on the five major tissue compositions, including tumor, stroma, CD8ɑ+, M1, and M2 phenotypes (Supplementary Figs. 12–13). The spatial relationship among cell types was quantified using cell neighborhood analysis within a distance of 30-µm (Supplementary Figs. 12–13). The distance of 30-µm was chosen empirically to best reflect the spatial connectivity of single-cells to capture ligand-receptor interactions, soluble cytokine interactions, and the varying cell size range of cancer and immune cells (Supplementary Fig. 14). Each segmented cell centroid was extracted as a node position and the node was associated with the cell type based on the maximum intensity of markers. Cell pairs within a 30-µm distance of each other are connected with an edge (Fig. 4a). This approach resulted in cell proximity graphs where nodes represented single cells and edges indicated spatial proximity and cellular neighborhoods. Cell proximity maps were overlaid on the tissue images to show the distinct composition of each patient’s tissue (Fig. 4b), revealing cell pair spatial neighboring between distinct tissue regions. The immune composition of the patients’ tissues was quantified by the node counts of each cell type including tumor, stroma, CD8ɑ+ cells, M1 cells, and M2 cells. SpatialVizScore assigned a final immunoscore to each region of interest (ROI) based on the spatial neighboring between tumor, stroma, and immune regions using the total cell counts. The tissue composition rankings were mostly tumor and stromal cells followed by CD8ɑ+ cells, M1 cells, and M2 cells (Fig. 4c). The neighborhood analysis showed that all patients’ samples have a strong spatial neighboring between tumor regions and CD8ɑ+ cells; however; a few patients showed a high spatial neighboring with tumor residues in their metastatic lymph nodes and CD8ɑ+ cells. Patients with consistently high CD8ɑ+ cells infiltration within their lung tumors and metastatic lymph nodes are C3/G3, D1/H1, and D5/H5 (Fig. 4d). Tumor samples D5, D9, and D1 yielded the highest tumor-M1 spatial neighboring while this level of spatial neighboring was not found for the metastatic lymph nodes of these patients (Fig. 4d). On the other hand, the level of tumor-M2 spatial neighboring was lower than that of tumor-M1 with relatively high spatial neighboring in tumor samples C3, D1, D9, and D5. The tumor-M2 spatial neighboring in the matched metastatic lymph nodes was different from that of the primary tumor samples (Fig. 4d). Collectively, these results indicated the heterogeneity in the immune composition among patients’ samples.

**Fig. 5:**
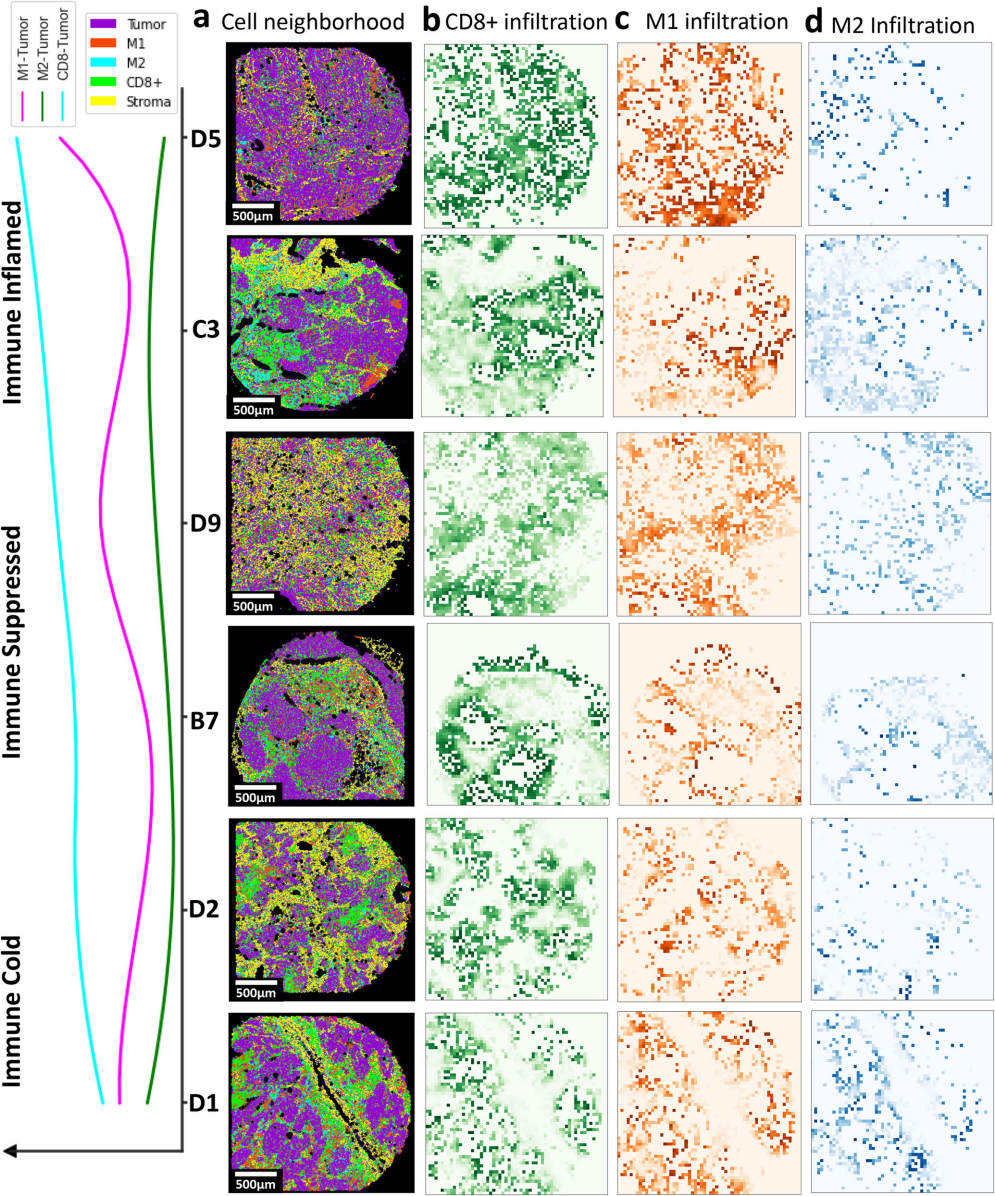
Spatial cellular interaction maps and SpatialVizScores of the tumor microenvironment demonstrate the immune continuum in lung cancer tissues. **a** Cell network and spatial proximity maps on lung cancer samples provided the interaction of tumor/T (in magenta), stroma/S (in yellow), CD8α (in green), M1 (red), and M2 (cyan). **b** Spatial interaction map demonstrated the distribution of tumor CD8α cells. It follows a continuum where it’s highest for immune inflamed and immune suppressed tumors but damps down for immune cold tumors. This is further validated by the line graph showing the distribution of the interaction among tumor regions, CD8α, M1, and M2 ranked by CD8α-Tumor score. **c** Spatial interaction map indicated the distribution of tumor and M1 cells. It follows a continuum where it’s highest for immune inflamed tumors, but damps down for immune suppressed tumors, and tapers off for immune cold tumors. **d** Spatial interaction map provided the distribution of tumor and M2 cells. It follows a continuum where it was the highest for immune suppressed tumors, but decreased for immune inflamed tumors, and tapered off for immune cold tumors.

The spatial proximity maps of the patients’ samples (Fig. 5a) were further used to generate infiltration maps of CD8ɑ+ (Fig. 5b), M1 (Fig. 5c), and M2 (Fig. 5d) in lung tumors. After ranking the tumor samples based on the number of spatial neighboring between Tumor and CD8ɑ+ cells, M1 infiltration distribution was found to be highly matched with that of CD8ɑ+, indicating immune inflamed tumors. Further, M2 was found to have the highest infiltration in tumor samples with mediocre CD8ɑ+ and M1 infiltration, identifying immune suppressed tumors. Finally, several tumor samples exhibited low infiltration for all three immune phenotypes, alluding to immune cold tumors. SpatialVizScore generated immune cell infiltration maps using several cancer markers (pan-keratin & e-cadherin), stromal markers (SMA & collagen 1), and immune markers (CD8ɑ, CD68, CD163, CD206, and HLA-DR).

The immune infiltration exhibited a continuum distribution that ranges from immune cold tumors with scarce immune cell infiltration, to immune suppressed tumors with the inhibitory immune microenvironment, and immune hot tumors with inflamed tumor immune microenvironments. Immune inflamed tumors showed a high presence of TILs and the pro-inflammatory M1-polarized macrophages whereas immune-suppressed tumors yielded M2-polarized macrophages. As expected, immune cold tumors exhibited neither TILs nor TAMs in patients’ tissues (Fig. 5)^25,26^.

The immune cold tumor samples are D1 and D2 as they showed dispersed spatial neighboring maps for the tumor and CD8ɑ cells, M1, and M2 cells (Supplementary Table 3). Besides, B7 and D9 tissues exhibited dense tumor-M2 spatial maps and high tumor-M2 scores (Fig. 5 and Supplementary Table 3), demonstrating immune-suppressive microenvironments that favored infiltration and polarization of TAMs into the M2 phenotype. Finally, D5 and C3 tissues provided immune inflamed states defined by the dense tumor-CD8ɑ and tumor-M1 spatial neighboring maps and the high T-CD8ɑ and tumor-M1 scores (Fig. 5 and Supplementary Table 3).

### Correlation of spatially variant immunoscores between primary lung cancers and their matched metastatic lymph nodes

**Fig. 6:**
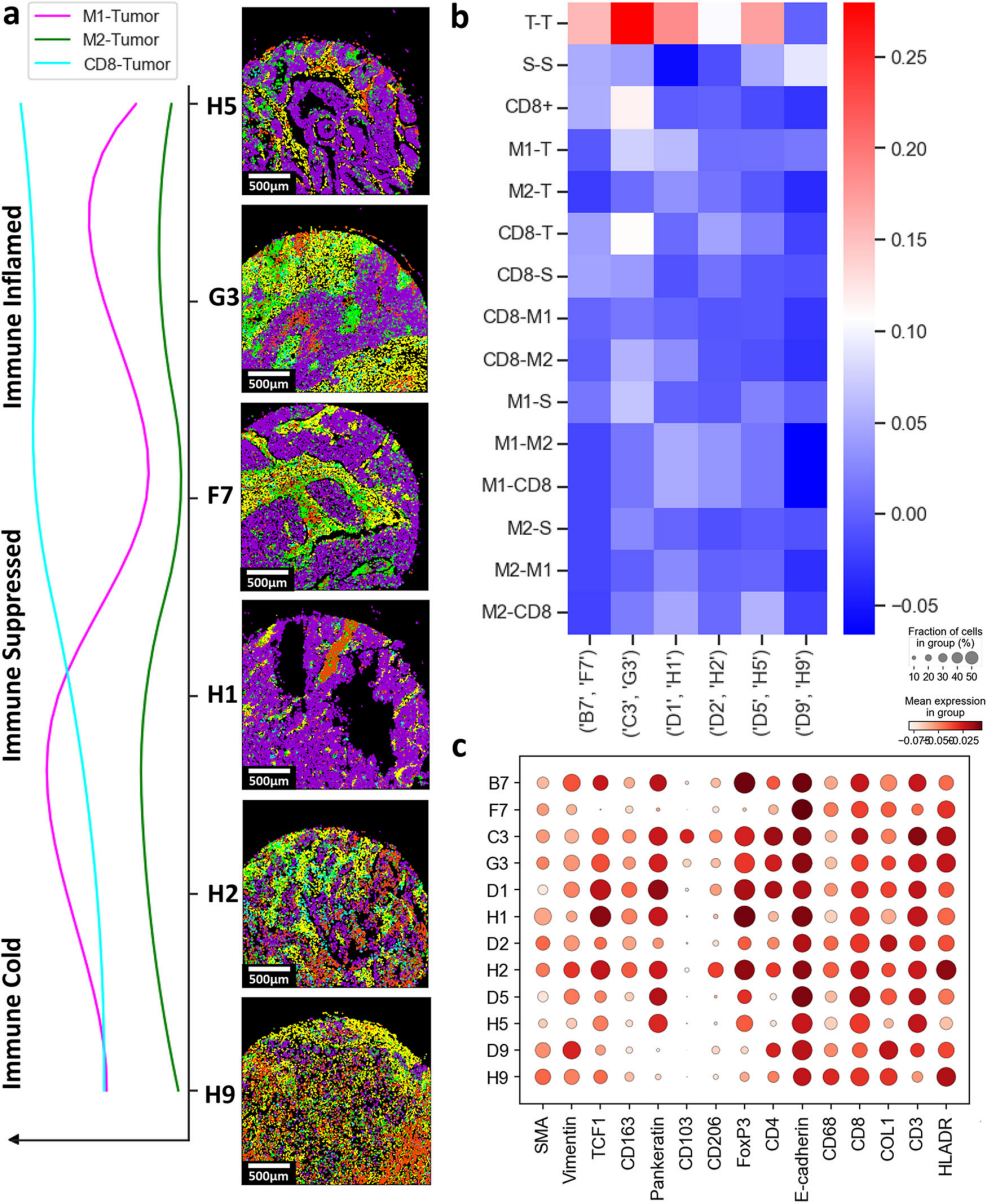
Correlation of the SpatialVizScores between lung cancer samples and their matched metastatic lymph nodes. **a** Cell network graphs on patients’ samples were demonstrated. Magenta represents tumor regions, yellow represents stromal regions, green represents CD8α cells, red represents M1 cells, and cyan represents M2 cells for the primary tumor and their matched metastatic lymph node samples. A line plot showed the continuum of immune-tumor interactions amongst the immune categoriesgorizes. Tumor-M1 interactions were shown in magenta, Tumor-M2 interactions were show in green, and Tumor-CD8α interactions were shown in cyan. **b** Heat map provided the correlation in immunoscores for each matching tissue pair of the primary tumor and matched metastatic lymph node. **c** Dot plot demonstrated the expression of markers across the primary lung cancer samples and their matched metastatic lymph nodes.

The matched metastatic lymph nodes of the primary lung cancers were further investigated with the same SpatialVizScore approach. Largely, the immune infiltration pattern within the matched metastatic lymph nodes agreed well with that of the primary tumors (Fig. 6a). D5/H5 and C3/G3 pairs were found to be immune inflamed whereas B7/F7 was determined to be immune suppressed and D2/H2 was identified to be immune cold. Two patients were recognized to have different immune infiltration patterns between their primary tumors and their matched metastatic lymph nodes including D1/H1 and D9/H9. Both D5/H5 and C3/G3 had a high correlation between their T-CD8 and tumor-M1. However, B7/F7 exhibited a high correlation between their tumor-M2 scores (Fig. 6b). This relationship could be attributed to the markers’ expression across the tissues such that D1 and H1 have variable CD206 and CD8ɑ expressions. Similarly, D9 and H9 have variable CD8ɑ and CD68 expressions (Fig. 6c).

### Pixel-level analysis for spatial scoring in the lung tumor microenvironment

**Fig. 7:**
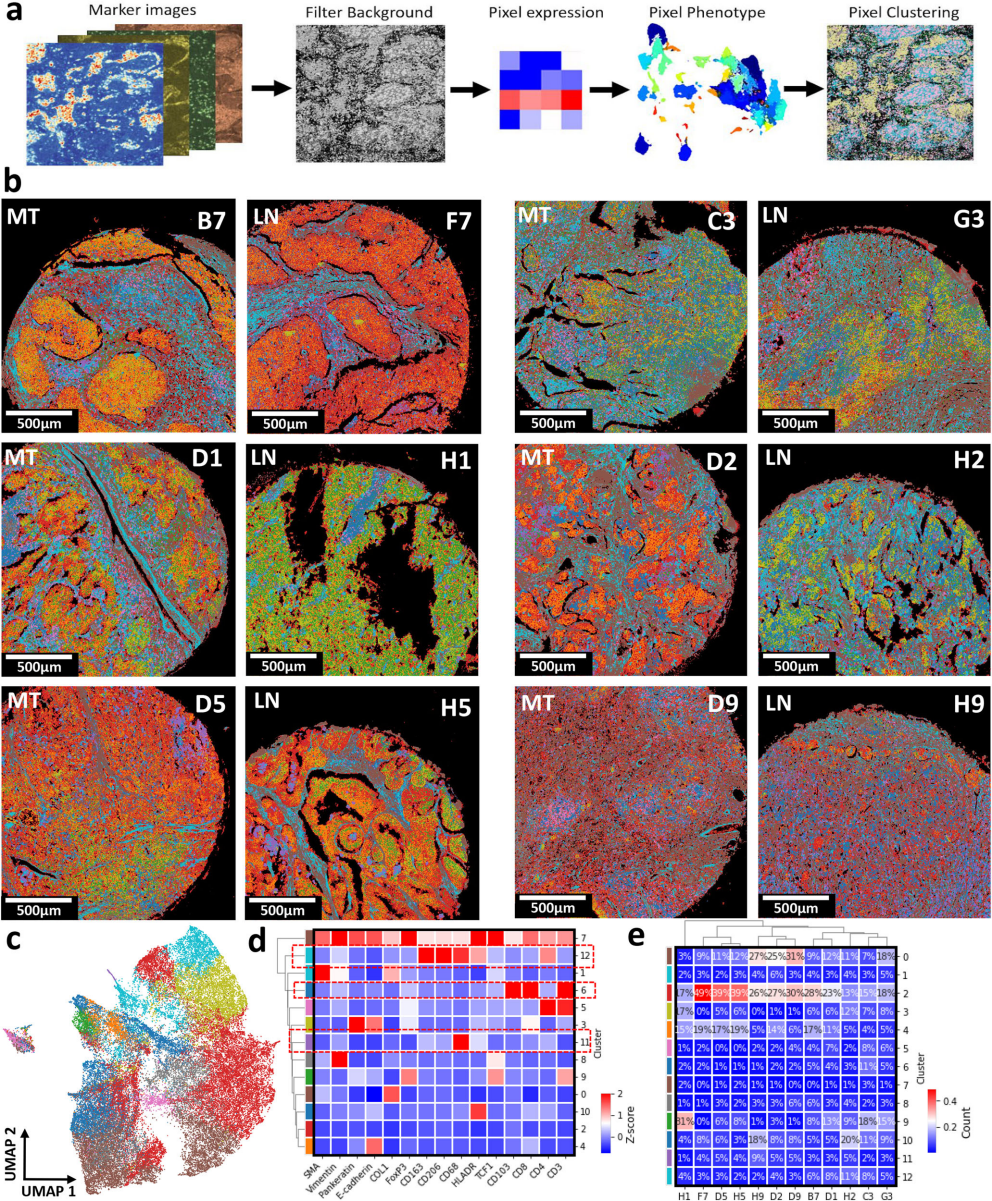
Pixel-level classification and clustering reveal the distinct tissue compositions and marker distribution within lung cancer patients’ samples. **a** Representative schematics demonstrates the process of pixel classification. The intensity information for all markers at all pixel locations (pixel size = 1 µm) was extracted and normalized. The background signal was filtered by eliminating pixel locations with an intensity value lower than 0.3. The resulting marker intensity data considered as non-background was clustered. The number of pixels was *n* = 5,093,784. Created with BioRender.com. **b** The resulting pixel intensity data were clustered using a subset of markers. The resulting clusters were visualized on the tissue samples by attributing each pixel to the corresponding cluster color, yielding distinct separation of different tissue regions. **c** UMAP displayed the distribution and the separation of the resulting 13 clusters from the pixel-level phenotypes. **d** Correlative heatmap presented the co-expression of markers across the 13 clusters that make up the data set. **e** Marker abundance heatmap yielded the cluster compositions for all patients’ cancer tissue samples and their matched metastatic lymph nodes.

Another pixel-level clustering approach was used on the patients’ multiplex image data to further obtain higher sub-cellular accuracy. The pixel-level phenotype was represented as a vector of length equal to the number of markers with a value equal to the marker intensity levels. Pixel locations where all marker expression levels are low are considered as background. Then, a combinatorial graph-based clustering approach was used to cluster individual pixels by the phenotype of their marker expression level (Fig. 7a and Supplementary Fig. 15). The representative images of patients’ tumor samples were reconstructed by assigning pixels to their corresponding clusters (Fig. 7b).

We employed unsupervised pixel-level clustering with all the 26 markers included in the dataset, resulting in 13 unique clusters with variant marker expressions identified by a Uniform Manifold Approximation and Projection (UMAP) visual and Leiden data clustering^24,27^ (Fig. 7c, d). Cluster 11 showed the co-expression of CD68 along with HLA-DR and lower expression of CD163 and CD206 which could be attributed to the M1-polarized macrophages. Further, cluster 12 indicated the co-expression of CD68 and CD163 and CD206 and lower expression of HLA-DR which corresponds to M2-polarized macrophages. Cluster 6 demonstrated the expression of CD8ɑ along with CD3 and CD103 corresponding to the tissue-resident memory T-cells (Fig. 7d). Patients D2, D9, and D5 exhibited high infiltration of cluster 11 or the M1-polarized macrophages. Interestingly, their metastatic lymph nodes H2, H9, and H5 showed a high composition of cluster 11 as well (Fig. 7e). Patients D1 and C3 showed higher infiltration of cluster 12 or M2-polarized macrophages, which lined up with their metastatic lymph nodes H1 and G3 samples as well. Although the metastatic lymph node sample H2 had one of the highest infiltration percentages for cluster 12, this fails to match with that of the primary tumor D2 (Fig. 7e).

The pixel-level classification provided a better separation between the unique clusters and cellular phenotypes. This can be attributed to a better segmentation of different cell types with varying shapes, sizes, and orientations with pixel-level classification and clustering. It is rather challenging to develop a representative cell segmentation mask using IMC data due to the resolution (1-µm) as compared to simple fluorescent microscopes achieving a resolution in the <300-nanometer range. Further, patients’ tissues have a myriad of cell types with varying morphologies, making the process of cell-based segmentation cumbersome. For example, some cell types also have asymmetric morphologies including stromal cells, complicating the cell segmentation tasks. Cell-based segmentation also relies on extracting the boundaries of the cells where there is a change in the gray-level intensity of the pixel values. This segmentation approach can be challenging to perform when cells have varying shapes and sizes or if two edges are touching and have a weak edge gradient^28,29^.

**Fig. 8:**
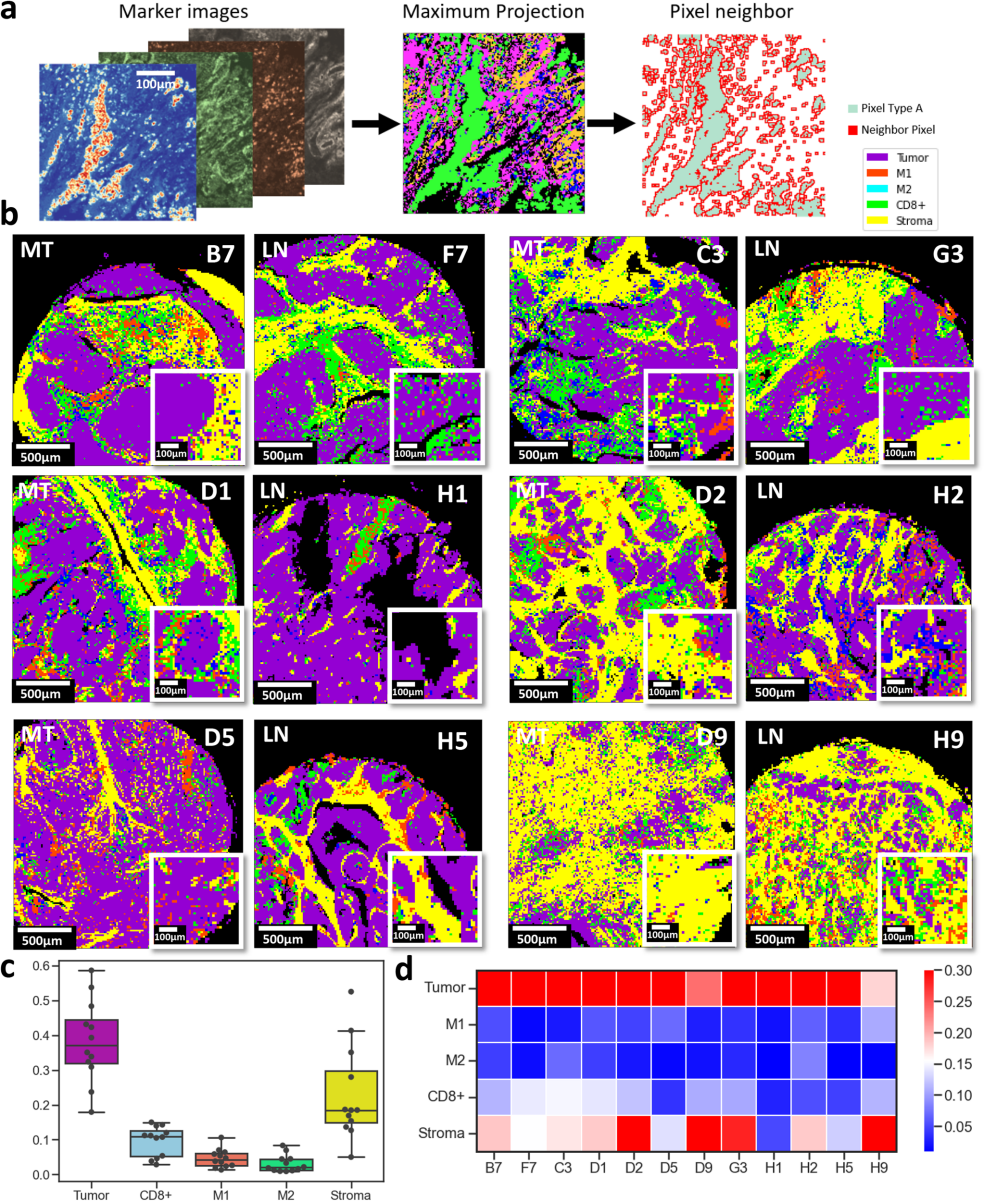
Spatial neighborhood maps and SpatialVizScores of the lung tumor microenvironment using pixel-level classification of cancer, stromal, and lymphocyte regions. **a** Pre-identified marker images were used to mark distinct anatomical regions (Tumor: pankeratin and e-cadherin, Stroma: SMA and collagen Type 1, CD8ɑ+ cells: CD8ɑ, M1 cells: CD68 and HLA-DR, and M2 cells: CD68, CD206, and CD163. This resulted in 4 final images for each patient tissue. The maximum projection image was generated by dividing each ROI image into patches with pixel size = 5-µm where each patch was assigned a tissue region based on the maximum intensity value of the pre-identified markers list. Pixel neighborhood analysis was then performed within the unique anatomical regions’ organization. **b** Maximum projection images of ROIs were shown. Magenta signifies tumor regions, yellow represents stromal regions, green demonstrates CD8ɑ+ cells infiltrated regions, red indicates the M1 infiltrated regions and cyan yields the M2 infiltrated regions. **c** Box plot demonstrates the tissue composition of patients’ samples. Box plot showing the distribution of the data with minimum, first quartile (Q1), median, third quartile (Q3), and maximum. Original Data was overlaid on the boxplot. Tumor regions were the most predominant followed by stromal regions, CD8ɑ, M1, and lastly M2 regions (*N* = 12). **d** Heatmap displays the density of the anatomical regions across the patients’ cancer samples and their metastatic lymph nodes.

An alternative tissue neighborhood reconstruction was implemented at the pixel level by dividing each ROI into patches of 5 by 5 pixels and the marker mean expression level in each patch was calculated. The maximum intensity projection of multiplexed data was used to determine the patch type between tumor (Pan-Keratin+E-cadherin), stroma (COL1+SMA), CD8ɑ, M1 (HLA-DR+CD68), and M2 (CD68+CD163+CD206) (Fig. 8a). The resulting tissue images were reconstructed by assigning each 5 by 5-patches to the corresponding type (T, S, CD8ɑ, M1, and M2). This approach better captured the continuum between the variation of regions as it relied on pixel-level reconstruction rather than cell-level segmentation and clustering. The mean intensity of each marker in patches of 5 pixels created a downsampling process and filter noise from the marker imaging data. The pixel reconstruction captured the multiplex marker images better with less background noise as introduced by the single-cell level segmentation (Fig. 8b). Similar to the cell segmentation results, the tissue composition was mostly covered by T, S, CD8ɑ, M1 macrophage, and finally M2 macrophage enriched regions (Fig. 8c, d).

### Comparisons of the spatially variant scores from single cells and pixels

**Fig. 9:**
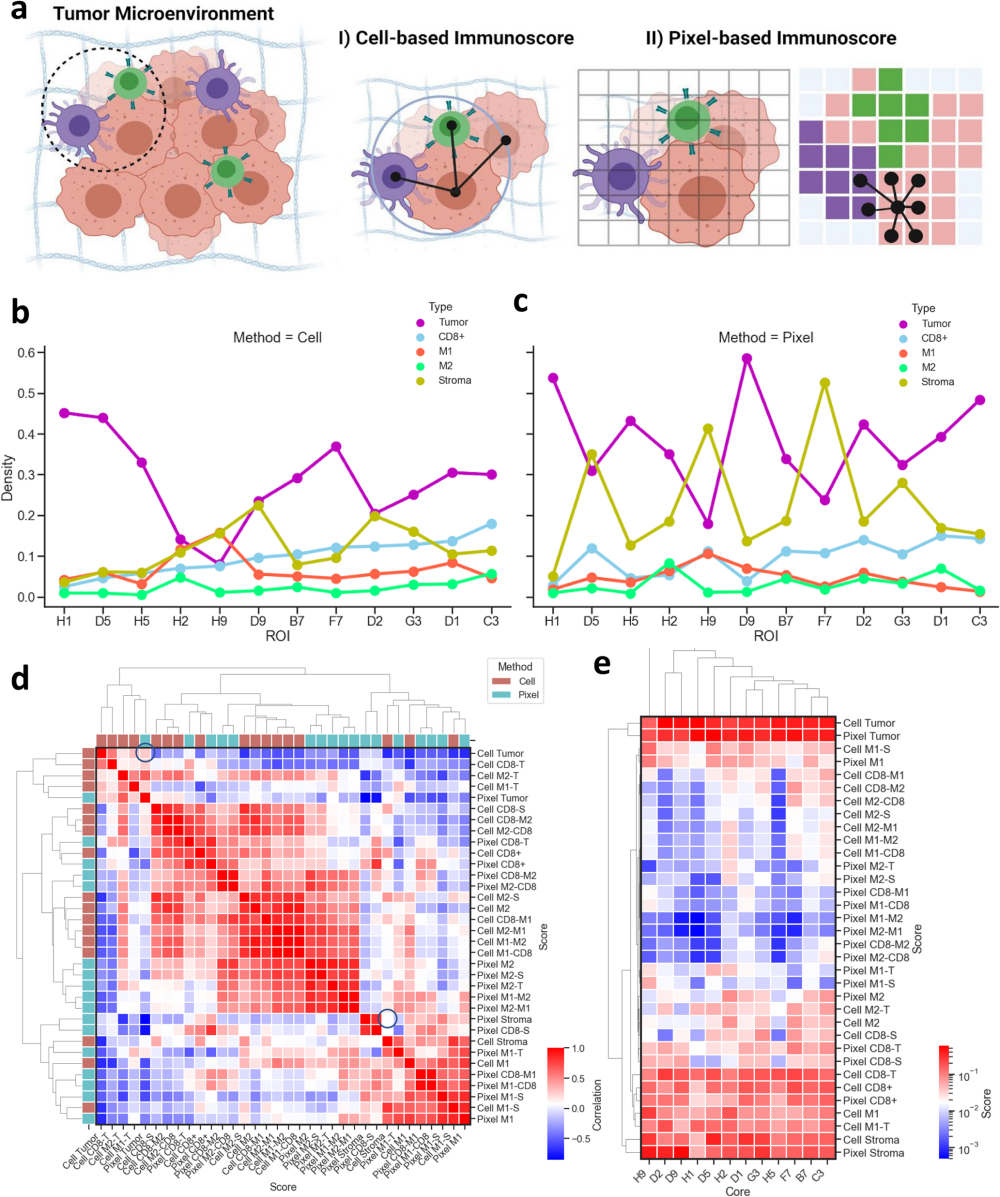
Cell-level segmentation and pixel-level classification enable distinct and spatially variant immunoscores in lung cancer patients’ samples. **a** Representative schematic shows the difference between cell-based and pixel-based immunoscoring. The cell-based immunoscore relied on cell segmentation masks to identify individual cells. The cell network was generated by identifying the distance between cell centroids within a diameter of 30-µm distance. The pixel-level immunoscore relied on the pixel-based classification, where the 1500-µm × 1500-µm patients’ tissues were divided into 300 × 300 square patches with patch size = 5-µm. Each patch was assigned a phenotype based on the highest intensity of marker expression. Similar to the cell network, the pixel network was generated to assess the tissue regions’ neighborhoods. Created with BioRender.com. **b** Line chart displays the density of the 5 different anatomical regions and phenotypes across patients’ cancer samples and their metastatic lymph nodes using the cell-based segmentation. **c** Line chart indicates the density of the 5 different anatomical regions and phenotypes across patients’ cancer samples and their metastatic lymph nodes using the pixel-based classification. **d** Correlation heatmap was shown between all the generated scores using the two methods: Cell-based segmentation and pixel-based classification. **e** Correlation heatmap was presented among all the generated scores for all patients’ tissues using the two methods: Cell-based segmentation and pixel-based classification.

The efficiency of the pixel classification to segment distinct tissue regions was further observed in different types of lung cancer tissues (Fig. 9a). Cell segmentation masks can undersegment immune and stromal cells, especially in dense tumor regions due to the varying morphologies of cells in the tumor microenvironment (Fig. 9b, c). Cell segmentation also fails to distinguish between different morphological regions such as the peripheries of T and S regions (Supplementary Fig. 16). Cell segmentation can also undersegment or oversegment cells at the stromal regions due to the dense fibrous alignment but this information can be efficiently preserved in the pixel classification (Supplementary Fig. 16). Finally, it is critical to choose the optimum patch size for the pixel classification that can detect markers’ expression signals with high sensitivity and low background noise much like setting thresholds for cell circularity and cell size throughout the segmentation process. Small patch sizes can detect noisy signals and unclear separation between tissue regions. Practically, 5-µm was found to be the most optimum after testing several patch sizes (Supplementary Fig. 15).

The SpatialVizScores generated from the cell segmentation and the pixel classification correlated with each other across patient tissue samples (Fig. 9d, e). In other words, comparisons of several immune spatial neighboring scores from cell segmentation and pixel classification showed similar results. This level of agreement was strongest in the case of detecting tumor regions as detected by the high correlation between segmented tumor regions by cell segmentation and pixel classification. The correlation was weaker in detecting stromal regions, demonstsrating that the pixel-level classification could be superior to cell segmentation in detecting highly heterogeneous cell populations, including CD8ɑ, M1, and M2 cell spatial neighboring with cancer cells (Fig. 9d, e and Supplementary Fig. 16).

### Cell phenotype enrichment model in distinct inflamed patient groups

**Fig. 10:**
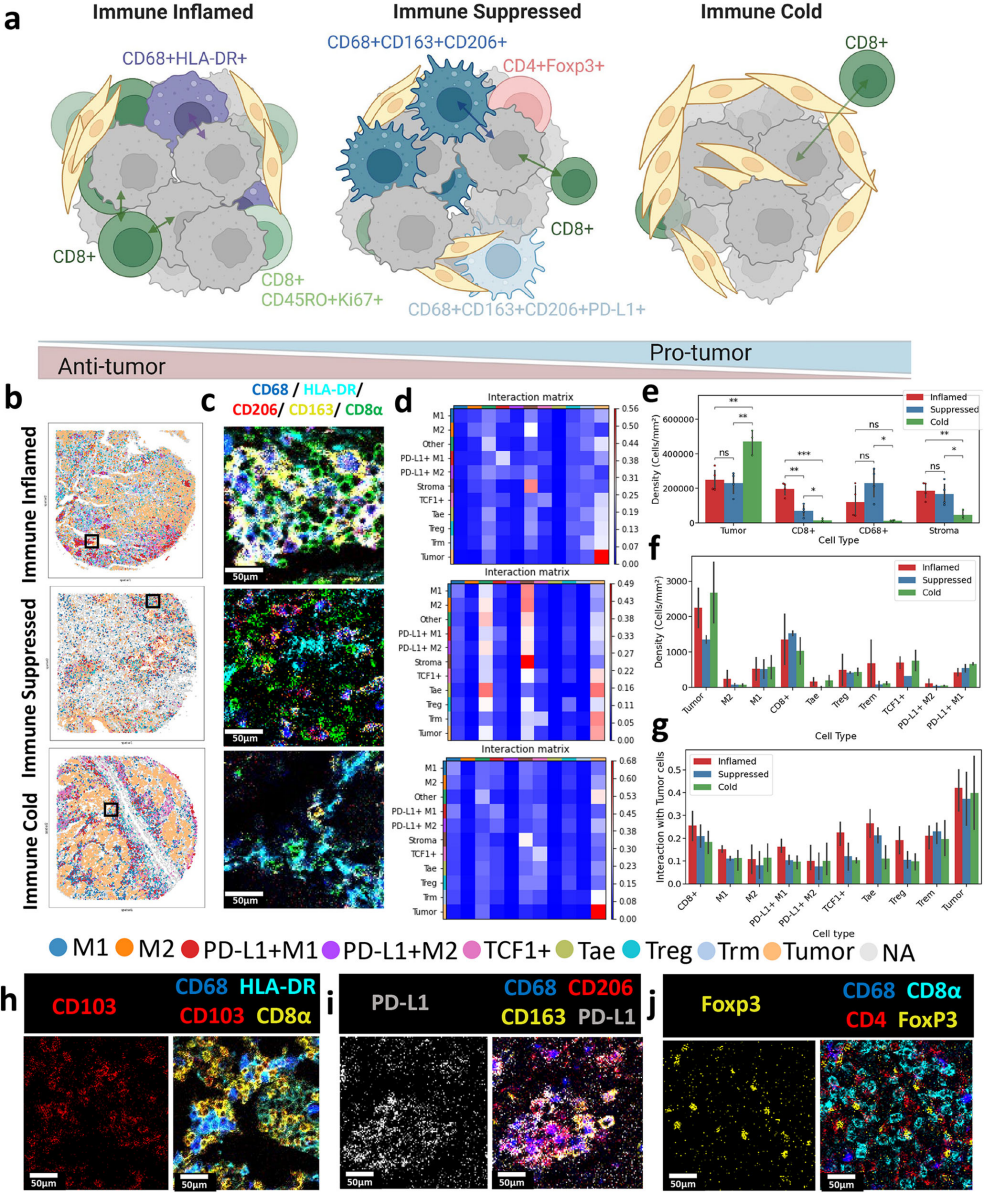
Cell phenotypic enrichment model of T cell and macrophage subsets is distinct in immune inflamed, suppressed, and cold tumors. **a** Representative schematic presents the composition of immune inflamed tumors with high infiltration of cytotoxic tumor-infiltrating lymphocytes and M1-polarized macrophages, immune suppressed tumors with high infiltration of M2-polarized macrophages, and low infiltration of CD8+ cells, and immune cold tumors with high fibrotic tissues and scare infiltration of immune cells. Created with BioRender.com. **b** Representative tissue infiltration maps for immune inflamed, immune suppressed, and immune cold cancers with different immune phenotypes were demonstrated. **c** Visual representation of IMC images demonstrated the infiltration of M1 (CD68 in blue, HLA_DR in cyan) and M2 macrophages (CD68 in blue, CD206 in red, CD163 in yellow) phenotypes alongside CD8+ (in green) T-cells for immune inflamed, immune suppressed, and immune cold tumors. **d** Interaction matrix was shown between different anatomical regions and immune cell phenotypes for immune inflamed, immune suppressed, and immune cold tumors. **e** Bar plot indicates the density of tumor cells, stromal cells as well as CD8ɑ+, CD68+ in patients cohorts 1 and 2 categorized as immune inflamed (red), immune suppressed (blue), and immune cold (green). The error bar shows a 95% confidence interval. **f** Bar plot demonstrates the density of additional immune phenotypes in patients cohort 2 categorized as immune inflamed (red), immune suppressed (blue), and immune cold (green). The error bar shows a 95% confidence interval. **g** Bar plot provides the interaction between subsets of immune cells and tumor cells in patients cohort 2 categorized as immune inflamed (red), immune suppressed (blue), and immune cold (green). Asterisk indicates the statistical significance for pairwise comparison. *P*-value calculated using Wilcoxon Rank Sum Test (ns: 0.05 < *p*, *: 0.01< *p* <= 0.01, **: 0.001 < *p* <= 0.01 ***: 0.0001 < *p* <= 0.001, ****: *p*<=0.0001). Error bar shows 95% confidence interval. **h**–**j** Visual representations of IMC images for 3 different Fields of View (FOV) were presented for the immune inflamed, suppressed, and cold tumors. **h** Tissue-resident memory T-cells were visualized. CD103 in red, CD68 in blue, HLA-DR in cyan, and CD8α in yellow. **i** PD-L1 M2 macrophages were visualized. PD-L1 in white, CD68 in blue, CD206 in red, and CD163 in yellow. **j** Regulatory T-cells were visualized. Foxp3 in yellow, CD68 in blue, CD8α in cyan, and CD4 in red.

SpatialVizScore has demonstrated distinct phenotypic distributions of immune cells in lung cancer patients’ samples (Fig. 10a). Immune cold tumors showed scarce immune cells within the tumor core or at the tumor peripherals, most likely due to poor neoantigen presentation and poor immune cells recruitment or secretion of immune repelling chemokines, or the overexpression of FasL that might result in immune cells apoptosis^26^. Immune suppressed tumors demonstrated moderate to high infiltration of immune cells with suppressive immune cells being the most predominant including M2-polarized TAMs, matching the previous reports associating M2 macrophages with immunosuppressive microenvironments for lung cancers^30^. Immune inflamed tumors exhibited a higher infiltration of TILs as well as M1-polarized TAMs associated with a pro-inflammatory tumor microenvironment that plays an important role to destroy cancer cells^30^ (Fig. 10b, c, e). By combining patients from cohorts 1 and 2 (*n* = 26), there was a significant (*p* < 0.05) difference in the infiltration of CD8+ cells within all patients’ groups (Fig. 10e).

The phenotypic enrichment included tissue-resident memory T-cells (Trm) that were characterized by their expression of CD8+CD103+^31^ (Fig. 10h and Supplementary Fig. 17). Trm cells showed higher infiltration of the cell density/area within the immune inflamed tumors (Fig. 10f). Further, the Trm cells demonstrated higher spatial neighboring with tumor cells within the immune inflamed tumors including C3 (Fig. 10d, g).

The proliferating antigen-experienced T-cells (Tae) are marked by their expression of Ki67+CD45RO+CD8+^32^ (Supplementary Fig. 17). They exhibited higher infiltration of the cell density/area within the immune inflamed tumors including D5 and C3 (Fig. 10f). Similarly, they showed higher spatial neighboring with the tumor cells within the immune inflamed tumors (Fig. 10d, g).

PD-L1+M2 macrophages were identified by the expression of CD68, CD163, CD206, and PD-L1(Fig. 10i and Supplementary Fig. 17). PD-L1+M2 macrophages lacked significant difference in their infiltration density or tumor spatial neighboring between the immune suppressed or the immune cold tumors (Fig. 10d, f, g). Further, the regulatory T-cells (Treg) were marked by their expression of CD4+ FoxP3+ (Fig. 10j and Supplementary Fig. 17). Treg cells demonstrated high infiltration within all the immune categories. Despite being insignificant due to the low sample size, Treg cells had the highest spatial neighboring scores for the immune inflamed tumor samples (Fig. 10d, f, g). We analyzed additional markers to identify the stem-like T-cells with CD8ɑ and TCF-1 expression^33^. However, there was an unexpected expression from the TCF1 in double-negative cells (CD8ɑ- CD4-), which is not the main focus of this paper and is currently under active investigation.

## Discussion

In this highly multiplexed single-cell study of the tumor immune architecture of lung tumors, we have quantified the heterogeneity of the lung cancer landscape and revealed the phenotypic enrichment of immune cell subsets infiltrating lung tumors. The highly multiplexed IMC has recently been used to profile the tumor microenvironment of several cancer types including breast cancer, lymphomas, melanoma, and lung cancer^12,34–36^. However, IMC lacked a quantifiable spatially variant tool for the immune infiltration pattern into patients’ tumors. Thereby, we sought to use IMC to investigate the tumor immune microenvironment to meet the increasing need to develop more efficient patient stratification tools, especially for patients undergoing immunotherapy treatments (Supplementary Table 4). The SpatialVizScore maps from IMC analysis offer new possibilities for spatial organization analysis of many cell types including tumor, stromal, and immune cells while preserving the native tissue microenvironment in cancer tissues^8,10^.

SpatialVizScore was developed as a companion computational tool along with the highly multiplexed IMC data to quantify and visualize the immune infiltration. In prior reports, CD8ɑ was consistently used to identify TILs and their counts were associated with longer disease-free survival (DFS) and/or improved overall survival (OS)^26^. The spatial proximity of the TILs and the tumor regions were further associated with favorable immune checkpoint inhibitor response^32^. CD8 positive cells have demonstrated several subsets of T-cells including the proliferating antigen-experience cytotoxic cells that exhibit different spatial infiltration patterns associated with cancer progression^32,37,38^. Furthermore, CD68+ was used as a pan-macrophage marker for tumor-associated macrophages (TAMs)^39^. Additional markers such as HLA-DR were included to identify the anti-tumor M1-polarized phenotype or CD163 and CD206 to define the pro-tumor M2-polarized macrophages. Under the several immunosuppressive signals in the tumor microenvironment, M1-macrophages can polarize into M2-macrophages which inhibit the cytotoxic activity of TILs by limiting their cytokine release and their proliferation which justifies using them as immunosuppressive cells^40^. Thus, it is crucial to consider M1 and M2 subtypes of CD68 positive cells in spatially variant immune scoring schemes for comprehensive immunotherapy designs.

Prior studies divided tumors into an immune “cold” state for low infiltration of immune cells or immune “hot” for highly infiltrative tumors^26^. However, the infiltration of immune cells follows a spectrum from immune cold tumors to immune-suppressed and immune inflamed tumors and later can further be classified into discrete states. This spectrum and discrete patient stratification were shown by major phenotypes of immune cells including the CD8+ infiltrating lymphocytes, M1-polarized, and M2-polarized macrophages. Several immune cell populations matched with the immunoscore distributions including inflammatory immune cell phenotypes (e.g., Tae), and suppressive immune cell phenotypes (e.g., PD-L1+M2, Treg). Trm cells (CD8+CD103+) were previously correlated with the high infiltration of M1-TAMs within lung tumors. The M1-polarized TAMs secrete CXCL9 to serve as a chemoattractant to Trm through its CXCR3 receptors and they elevate their update of unique fatty acids from the tumor microenvironment that are required for the survival and maintenance of Trm^31^. Besides, the infiltration of Tae cells matched our SpatialVizScore results. The Ki67+CD45RO+CD8+ proliferating Tae cells were shown to have higher infiltration in myeloma patients with better drug response and prolonged survival^32^. This highlights their importance in maintaining an anti-tumor immune microenvironment. Finally, we have additional markers to profile more phenotypes of T-cells, especially stem-like T-cells. However, we observed signals from TCF1 (stemness marker) in CD8ɑ- and CD4-.

To evaluate the immune checkpoints in tissues, the overexpression of PD-L1 and the engagement of PD-1/PD-L1 are known to lead to inhibiting TCR-mediated activation of T cells, inhibiting the section of T-lymphocytes cytokines, and eventually promoting T lymphocytes apoptosis^31^. Interestingly, PD-L1 was found to be expressed on M2-polarized TAMs that cause the suppression of the cytotoxic functions of CD8+ T cells^41^. In addition, CD4+Foxp3+ Tregs cells are an immunosuppressive sub-phenotype of T cells that functions to decrease inflammation and restore homeostasis. Tregs were previously correlated with consistent poor disease outcomes for several cancer models including lung cancers^5^.

To further illustrate the clinical utility of the SpatialVizScore, we have generated discrete patient stratification using numerical quantification of the immune continuum based on the CD8-T infiltration scores including low (−0.001, 2.255), medium (2.255, 6.695), and high (6.695, 10.0) in 26 human tissues from lung cancer patients, combining cohort 1 (CH1) of 6 primary lung tumors and 6 matched lymph nodes in the first tissue microarray, and cohort 2 (CH2) of 14 additional lung tumors in the second tissue microarray. These SpatialVizScores were further validated by the distribution of tumor-M1 and tumor-M2 scores. The same trend was observed for patients’ samples ranging from immune cold to suppressed to inflamed lung tumors (Supplementary Fig. 18a–c and Supplementary Tables 5–6). Further, the distribution of CD8-T exhibited a heterogeneous distribution for the tumor-M1 and the tumor-M2 scores in a 3D visual of 12 tissues, yielding an intricate M1 or M2 enrichment in distinct tumors in 3D score distributions (Supplementary Fig. 18d, e). This highlights the complexity of immune cell distribution in lung cancers between the inflammatory and the suppressive immune cell types. These findings support advances in the SpatialVizScore approach compared to the prior tools used for immunoscore that only rely on oversimplified CD8 scores.

SpatialVizScore was benchmarked against established data analysis and visualization tools. First, *CellPose* was integrated into the SpatialVizScore pipeline for single-cell segmentation and it relies on deep learning algorithms to precisely segment individual cells in 2D and 3D data without training or parameter adjustments^21^. This technique was benchmarked against *CellProfiler*, an established open-source software used to generate cell segmentation masks after adjusting the parameters for cell circularity and cell size^22^. Both segmentation techniques showed comparable results in segmenting cells in various tissue types from different patient donors. At the same time, *CellPose* was more robust in cell segmentation in diverse tissues (Supplementary Fig. 19). SpatialVizScore clustering pipeline was then benchmarked against *HistoCAT*, an open-source toolbox developed to analyze IMC data^42^. The cluster compositions from both methods indicated similar marker compositions whereas SpatialVizScore also visualized immune infiltration patterns in tumors (Supplementary Figs. 20 and 21). SpatialVizScore was then verified against *Giotto*, a toolbox designed to analyze and visualize single-cell spatial data (Supplementary Fig. 22)^43^. Although both pipelines could cluster the data based on marker expression, SpatialVizScore outperformed in visualizing the clusters on patients’ tissues and providing infiltration scores in human cancer tissues.

Further, SpatialVizScore is based on multiplexed protein data from IMC with an antibody panel of 26 markers. This panel targeted tumor, stromal, functional, and immune markers to dissect the tumor microenvironment in patients’ tumor samples. Prior immunoscore techniques relied on quantifying the infiltration of TILs from H&E stained tissue data that is only limited to the morphological analysis between major cell types (tumor, stroma, and immune cells) and fails to detect the different types of immune cells^44,45^. Further, other studies utilized IHC and immunofluorescence (IF) to quantify different immune markers at the tumor core and peripherals. However, these studies are limited to a few markers (1-3) that lack a fully comprehensive understanding of the immune infiltration states within patients’ tumors (Supplementary Table 7)^8,10,46–48^. Further, multiplexed IHC techniques suffer from non-quantitative or semiquantitative results, posing limits to the analysis of infiltration patterns of several immune cell types^49^. Therefore, IMC is superior to the routinely used lab techniques, making it more relevant to clinical applications.

One potential limitation of this study was that we examined small tissue samples from the patients (1500-µm × 1500-µm), yielding a decent coverage of the full tumor-immune landscape to capture several morphological regions within this area with a resolution of 1-µm. Multiplexed images were able to discern the heterogeneity of the tumor microenvironment and describe a consistent continuum pattern and discrete classification of the infiltration of the immune cells in lung tumors.

Besides, the immune panel can be expanded to further analyze more phenotypes within the macrophage spectrum that ranges from M1 to M2a, M2b, M2c, and M2d and relate them to cancer progression and the disease outcome (Supplementary Fig. 23). Additionally, several papers showed the significance of the M2/M1 ratio rather than the discrete count of the phenotype^14^. Higher M1/M2 ratio was generally associated with a favorable prognosis including longer overall survival and better response to chemotherapy and radiotherapy. Lower M1/M2 ratios were associated with poor response to chemotherapy and radiotherapy and shorter overall survival^14^. Thereby, M2/M1 ratio could also be incorporated within SpatialVizScore to investigate their correlation with the immune infiltration pattern in cancer tissues (Supplementary Fig. 24).

Another aspect of this study was the lack of information related to the treatment history of the patients as it can alter the immune infiltration within the tumors and can impact the final immunoscore generated by SpatialVizScore. Additional validations with more patient data including prior treatment, response, and disease progression would enrich the immune scores generated by SpatialVizScore. Combining SpatialVizScore with more patients’ clinical data along with the multi-omics profiling of the biopsies will be powerful in further validating this emerging tool and moving it to clinical practice. In an attempt to validate SpatialVizScore in clinical samples with drug response, we applied the same computation tool in quantifying the immune infiltration pattern within breast cancer H&E images with the drug response data to immune checkpoint inhibitors (ICIs) (Supplementary Fig. 25 and Supplementary Table 8). Different cellular phenotypes (Tumor, Stroma, and Immune Cells) were identified based on their variant morphologies and the same tissue neighborhood scoring approach was tested in H&E stained tissues of triple-negative breast cancer (TNBC) tumors. Responder patients (*n* = 3) exhibited higher SpatialVizScore values within their tumors than non-responder patients (*n* = 3), validating our computational rationale for SpatialVizScore. Of note, the ICI cohort included about 20 subjects and the rest of the patients exhibited partial response, which was not included in this analysis. Multiplexed IMC imaging of a larger cohort will be the subject of a follow-up study as more patients with complete ICI responses are obtained in the near term. We further benchmarked our SpatialVizScore approach against previously published approaches to generate TILs spatial infiltration maps in H&E stained tissues of lung tumors in the Cancer Genome Atlas (TCGA) histological imaging data (Supplementary Table 9). These TILs maps showed a decent agreement with cancer patients’ survival and immune infiltration profiles in a cohort of 26 patients^45^. SpatialVizScore generated similar infiltration maps compared to deep learning-based lymphocyte infiltration maps, further validating the presented immunoscoring pipeline (Supplementary Fig. 26).

To independently demonstrate the efficacy of SpatialVizScore, we analyzed 14 lung cancer patients (11 cancerous and 3 paracancerous/adjacent tissues) with variant cancer subtypes, stages, and grades in addition to patient CH1. We also applied the same SpatialVizScore approach using single-cell segmentation, clustering, cell neighborhood analysis, and infiltration scoring in lung tumors of CH2. Several clusters corresponded to unique cellular phenotypes including Treg cells for cluster 6, and Tae cells for cluster 4 (Supplementary Fig. 27). The same tissue neighborhood analysis was applied between tumor/epithelial, stromal, CD8+, and CD68+ regions (Supplementary Fig. 28) and revealed a similar trend for the immune infiltration spectrum. Immune cell infiltration within the patients’ tissues showed a continuum distribution that ranged from immune cold, to immune suppressed, and immune inflamed tumors that can then be used for distinct patient stratification (Supplementary Fig. 29). This distribution was further validated by the expression of other immune markers including FoxP3, Granzyme B, CD4, CD45RO, CD20, and CD3. Immune cold tumors exhibited a sparse distribution of all immune markers whereas immune suppressed tumors had a higher distribution of immune inhibitory markers including FoxP3 and CD4 which could be attributed to Treg cells. Further, immune inflamed tumors had higher expression of GranzymeB, CD45RO, and CD3 (Supplementary Figs. 30 and 31). Moreover, we applied the equivalent rationale of segmentation, clustering, and tissue neighborhood analysis to the tissues using pixel-based classification. The same cell phenotype clusters and patients’ immune infiltration patterns persisted using pixel-based segmentation (Supplementary Figs. 32 and 33). As expected, pixel-based segmentation outperformed cell-based segmentation in several regions. Cell segmentation also fails to distinguish between different morphological regions and can miss important information at the peripheries of tumors and stromal regions. Cell segmentation can also undersegment or oversegment cells in the stromal regions due to the dense fibrous alignment but this information can be preserved much more efficiently with the pixel classification (Supplementary Fig. 34). By comparing the immunoscores results from both approaches, they showed a high level of correlation between the different anatomical regions except for stromal-related scores (Supplementary Fig. 35).

In summary, SpatialVizScore provides a platform for the multiplex analysis of the IMC data to develop more efficient patient stratification methods. This can identify the patients as potentially responder candidates for immunotherapies based on their immune infiltration pattern to maximize the benefit of the administered ICI drugs. IMC analysis of the tumor immune microenvironment of human cancer tissues reveals the continuity of immune cell infiltration patterns and discrete patient stratification that can be used as a translational tool toward precision oncology^13,50^. This digital pipeline is applicable for multi-dimensional protein datasets acquired from single-cell multiplexed imaging technologies including IMC and potentially many others. The presented SpatialVizScore scheme quantified the immune spectra of lung cancer tissues and associated patients’ tumors into three main categories including immune inflamed, immune suppressed, and immune cold along a tumor immunity continuum axis. A higher dimensional panel would provide the multiparameter immunoscore including immune-stimulatory and immune inhibitory markers to further distinguish the immune subsets making up tumors’ complexity. This pipeline may apply to different cancer types, larger patient cohorts, and clinical information including survival data and treatment regimens.

## Methods

### FFPE tissues

Patients’ samples in cohort 1 were obtained from a tumor microarray (TMA) purchased from a third-party vendor (Biomax, US) with the tissue ID: LC10012a. This TMA included a total of 100 tissue cores of formalin-fixed paraffin-embedded (FFPE) non-small cell lung carcinoma, cancer adjacent tissues, and normal lung tissue samples obtained from 50 patients. Each tissue core had a diameter of 1 mm and a thickness of 5 µm which is within the tissue thickness recommended for IMC (≤7-µm). Patients’ samples in cohort 2 were obtained from a different TMA with the tissue ID: LC814a. This TMA contained a total of 80 FFPE lung carcinoma and their matched metastatic lymph nodes tissue samples were obtained from 40 patients. Each tissue core had a diameter of 1.5 mm and a thickness of 5 µm. We used pathologist annotation to select tissue cores with a high density of tumor cells, no necrotic tissues, and high immune infiltration. We chose 14 tissue cores from TMA: LC10012a and an additional 12 tissue cores from TMA: LC814a from different cancer stages and grades. Both TMA samples were collected with patients’ consent following high ethical and medical standards. All human tissues are collected under HIPAA-approved protocols. For both TMAs, the same tissue labeling procedure was followed as previously reported^51^, including the antigen retrieval, protein blocking, metal-tagged antibody labeling, and nucleus counterstains. First, the samples were baked in a 60 °C oven for 2 hours. Then, the samples were deparaffinized by immersing them in xylene and rehydrated by dipping them into descending concentrations of ethanol (100%, 95%, 80%, 70%, and 50% ethanol in water). Finally, the samples were washed with deionized water before proceeding with the antigen retrieval step. The heat-induced epitope retrieval (HIER) approach under basic conditions was used to achieve antigen retrieval using Dako’s target retrieval solution with pH = 9 (Catalog number: S2367, Agilent Dako). The slides were immersed in the target retrieval solution and were incubated in the pressure cooker in a high-pressure setting for 20 minutes. The slides were then left in the target retrieval solution for an additional 20 minutes at room temperature. The slides were washed with Maxpar Water (Catalog number 201069, Fluidigm) for 10 minutes followed by Maxpar PBS (Catalog number 201058, Fluidigm) for an additional 10 minutes with gentle agitation. To minimize the volumes of solutions used on the tissue samples, a PAP pen was then used to draw a hydrophobic barrier around the tumor microarray samples on the slides. Dako’s ready-to-use protein blocking buffer solution (Catalog number: X090930-2, Agilent Dako) was applied to the tissues and incubated for 45 minutes at room temperature. The slides were then washed 3 times with Maxpar PBS. The antibody cocktail mix was prepared in Dako’s protein-blocking buffer solution and incubated with the tissues overnight at 4 °C, following Fluidigm’s labeling protocol. The tissue slides were then washed with 0.2% Triton X-100 in Maxpar PBS for 8 minutes with gentle agitation followed by Maxpar PBS for additional 8 minutes. The nuclear staining was then performed using an Iridium-conjugated intercalator (Catalog number 201192 A, Fluidigm) prepared in Maxpar PBS for 30 min at room temperature in a hydration chamber. Finally, the slides were washed with Maxpar water and left to dry at room temperature for 20 minutes. After the staining process is complete, the stained tissues were stored at 4 °C until imaging time (Fig. 1).

### Whole-slide breast cancer H&E images

Patients at the Winship Cancer Institute of Emory University with advanced/unresectable or metastatic triple-negative breast cancer treated with an immune checkpoint inhibitor plus chemotherapy between 2019 and 2021 were retrospectively evaluated. Demographic, clinicopathologic, and outcomes data were obtained from the electronic medical record after approval by Emory University Institutional Review Board (Supplementary Table 6). The de-identified tissues were obtained by (J.A.) with the Emory IRB: STUDY00002958. The use of tissue samples is covered under a general protocol for studying specimens from the biorepository called 5231-21 Discovery of New Therapies and Treatment Outcomes for Breast Cancer and Related Neoplasms (Winship Discovery protocol).

### Data

To set up the Hyperion imaging system, regions of interest (ROIs) of 1500-μm × 1500-μm were chosen within each tissue core to cover most of the tissue. To choose the most optimum laser ablation power, several testing points were chosen from the tissue cores that represent the tissue heterogeneity. The acquired data was automatically saved in.mcd format that can be viewed using Fluidigm’s MCD Viewer software (v 1.0.560.2).

### Images

Each ROI image was extracted using MCD Viewer (v 1.0.560.2) with minimum threshold intensity of 0 and maximum threshold intensity of 50. Each image intensity range was then scaled to 0 and 99.9th intensity percentile for processing. When comparing the intensity range across ROI, the intensity was scaled to the 20th and 99th intensity percentile of all the marker images in the ROI to reduce high background staining in some ROIs.

### H&E validations

First, hematoxylin and eosin (H & E) images were obtained from serial sections of the tumor microarrays, serving as a validation of cancer-enriched regions. Next, from multiplexed images, each ROI H&E image was reconstructed. The average image from multiple markers was computed for both nuclei and cytosol. For nuclei, the markers consisted of Histone3, H3K9me3, Ki67, FoxP3, and intercalators conjugated to ^191^Ir and ^193^Ir. For cytosol, the markers included Vimentin, CD68, MHC-II, GranzymeB, CD20, E-Cadherin, and PanKeratin. For the nuclei reconstruction image, the colormap was transformed from matplotlib package ‘bwr_r’ colormap by selecting the upper half of RGB quantization levels of the colormap. The alpha was set using the NumPy function linspace going from 0 to 1 with *N* equal to the number of RGB quantization levels in the colormap. For the cytosol reconstruction image, the colormap was transformed from matplotlib package ‘PiGr_r’ colormap by selecting the upper half of RGB quantization levels of the colormap. The alpha was set using the NumPy function linspace going from 0.5 to 1 with *N* equal to the number of RGB quantization levels in the colormap. Finally, H&E stain reconstruction was achieved by superposing the nuclei reconstruction image on top of the cytosol reconstruction image.

### Segmentation

Single cells were segmented using *CellProfiler* software to extract the nuclei using the ^191^Ir and ^193^Ir intercalator signals. After a few iterations, the nuclei diameter range was set to range between 5–20 pixels to generate the nucleus segmentation masks. Further, the cell membrane was segmented by expanding the nuclear segmentation mask by 3 pixels to generate the cell segmentation masks. Separately, single-cell nuclei regions were also segmented using *Cellpose* by combining the intercalator signals from ^191^Ir and ^193^Ir with Histone3, Ki67, and FoxP3 marker expression images. The cytosol region was calculated by expanding the nuclei segmented region by 2 pixels. A distance of 2 pixels was obtained by taking the average of the major axis of the ellipse that exhibited the same normalized second central moments of the nuclei and cytosol region using *CellProfiler*.

### Cell clustering

Cell phenotypes were clustered using a Leiden algorithm^24^, a graph-based community detection algorithm. From each segmented cell region, the mean intensity of each marker expression was calculated. The resulting feature matrix consisted of *n* rows of the total number of cells (*n* = 264,191) and *p* columns of marker expression. Each column of the feature matrix was *z*-score normalized. The neighborhood graph was constructed and used for unsupervised community detection. Each cell was associated with a cell phenotype cluster and attributed a cluster color showing the cell-level clustering of each ROI.

### Pixel clustering

Pixel phenotypes were clustered in a two-step clustering pipeline. The image was first downsampled by a factor of 2 to filter out the noise in the marker expression data. Then, from each pixel location, the intensity value of each marker expression was extracted. To filter out the background, each pixel location with all markers intensity lower than 0.3 was considered as background and dropped from the feature matrix. The resulting feature matrix consisted of *n* rows of the total number of pixels (*n* = 5,093,783) and *p* columns of marker expression. Each column of the feature matrix was min-max normalized. The feature matrix was clustered using the Parc algorithm^52^ with the feature embedding visualized using UMAP. The mean expression from clusters resulting from the Parc algorithm was calculated. Hierarchical clustering was performed on the cosine similarity of the mean expression using the average linkage method with a threshold of 0.25 from the maximum pairwise distance. Each pixel was associated with a pixel phenotype cluster and attributed a cluster color showing the pixel-level clustering of each ROI.

### Spatial proximity network

From single-cell segmentation, cell centroids and mean expression levels within the cells were extracted. Each cell was assigned to the highest expression type (Tumor/Epithelial: ECadherin+Pankeratin, CD8+: CD8, M1: CD68+HLA DR, M2: CD68+CD163+CD206, and Stroma: Col1+SMA). Tumor/Epithelial regions were multiplied by 0.6 to take into account the overstaining factor and this was determined empirically for better cell type classification. CD8ɑ marker images were denoised using a median filter with a disk size of 1 pixel due to some ROI being immune cold with low CD8ɑ staining per cell resulting in higher noise before processing. Cell networks were created by connecting centroids within 30-μ*m* of each other, thus generating a spatial proximity network for each ROI.

### Spatial variant infiltration score

Each ROI was divided into a patch of 25 by 25-μ*m* area (equivalent to a 25-by-25 pixels area). For patch *i*, the total number of cells was defined as *n*_*i,cell*_, the total number of tumor cells was defined as *n*_*i,tumor*_. Therefore, tumor density of patch *i* was defined as $P_{i,tumor} = \frac{{n_{i,tumor}}}{{n_{i,cell}}}if\,n_{i,cell} \,>\, 0\,else\,0.$ Likewise, we defined the stroma density of patch *i* is as $P_{i,stroma} = \frac{{n_{i,stroma}}}{{n_{i,cell}}}if\,n_{i,cell} \,>\, 0\,else\,0.$

The cell centroid node in patch *i* was defined as *c*_*i,j*_ for $j \in [0,n_{i,cell} - 1]$. Let *N*_*i,j*_ be the neighbor node of *c*_*i,j*_ in the cell spatial proximity network. The number of the link between category A cells and B cells in patch *i* was defined as $L_{i,A - > B} = \mathop {\sum}\nolimits_{c_{i,j} \in A} {\mathop {\sum}\nolimits_{n \in N_{i,j}} {1_B\left( n \right)(1)} }$ with $1_B(n) = 1if{\kern 1pt} n \in Belse0$. In each patch region, the spatial neighboring score between two cell types was then defined as the number of edges of the two types divided by the total number of possible edges. This provided us with a 2D heatmap of spatial score and infiltration from cell to cell neighboring information. Two cells were considered neighbors if the centroid of the cell was within a distance of 30 pixels (equivalent to 30-µm). This distance for connecting neighborhood cells in the spatial graph was chosen empirically to best reflect the neighboring pattern in tissues. The distance parameter can be manually changed to suit different types of tissues with various cell densities. Single-cell marker variation plots were generated using the mean expressions of each marker and plotted by fixing the ROI (x-axis) in the same order as increasing the CD8+ T cell neighboring score.

### Spatial variant infiltration pixel score

Each ROI was divided by *n* by *n* pixels (*n* from 1 to 5). The intensity levels were rescaled between 0 and 1. For patch ***i***, the mean intensity of each of the 5 marker types (Tumor, M1, M2, CD8, and Stroma) was calculated, and each patch was assigned to the maximum mean intensity marker type. If all mean intensities of the 4 marker types were less than 0.1, the corresponding patch is considered to be the background. The resulting maximum projection image showed the pixel-level classified phenotype in each patch in each ROI. The density of type (Tumor, M1, M2, CD8, and Stroma) was defined as the number of patches of size *n* by *n* pixels with a maximum mean intensity of a specific type divided by the total number of patches. Two patches are considered to be neighbors if they are direct neighbors to each other in the reconstructed projection image. The immunoscore between two types is the number of neighbors between two patch types divided by the number of total possible neighbors.

### Reporting summary

Further information on research design is available in the Nature Research Reporting Summary linked to this article.

## Supplementary information

Supplementary materials

REPORTING SUMMARY

## Data Availability

The IMC image data supporting the findings of this study are available at 10.5281/zenodo.6784253. The mass spectrometry proteomics data have been deposited to the ProteomeXchange Consortium via the PRIDE^53^ partner repository with the dataset identifier PXD035340.

## Code availability

The IMC image processing codes are available at https://github.com/coskunlab/SpatialVizScore. The IMC data was exported using MCD Viewer (v.1.0.560.2). Analysis used Anaconda (v. 4.12.0) and Jupyterlab (v. 3.2.8).
