# Supplementary material for: Spatially variant immune infiltration scoring in human cancer tissues
Source: NPJ Precis Oncol. 2022 Sep 1;6:60. doi: 10.1038/s41698-022-00305-4 (PMC9437065; doi:10.1038/s41698-022-00305-4)
Supplement: Supplementary file 1 — Supplementary materials [file 41698_2022_305_MOESM1_ESM.pdf]

Spatially variant immune infiltration scoring in human cancer tissues

Mayar Allam[1,#], Thomas Hu[1,2,#], Jeongjin Lee[1], Jeffrey Aldrich[3,4], Sunil S Badve[4,5], Yesim Gökmen-Polar [4,5], Manali Bhave[3,4], Suresh Ramalingam[3,4], Frank Schneider[4,5], and Ahmet F. Coskun [1,4,6,7*]

[1] Wallace H. Coulter Department of Biomedical Engineering, Georgia Institute of Technology and Emory University, Atlanta, GA, USA

[2] School of Electrical and Computer Engineering, Georgia Institute of Technology, Atlanta, GA, USA

[3] Department of Hematology and Medical Oncology, Emory University School of Medicine, GA, USA

[4] Winship Cancer Institute, Emory University, GA, USA

[5] Department of Pathology and Laboratory Medicine, Emory University School of Medicine, GA, USA

[6] Interdisciplinary Bioengineering Graduate Program, Georgia Institute of Technology, Atlanta, GA, USA

[7] Parker H. Petit Institute for Bioengineering and Bioscience, Georgia Institute of Technology, 315 Ferst Dr. NW, Atlanta, GA, USA

[#]Equal authorship *Corresponding author

Ahmet F. Coskun, Ph.D. (ahmet.coskun@bme.gatech.edu)

Supplementary Figures

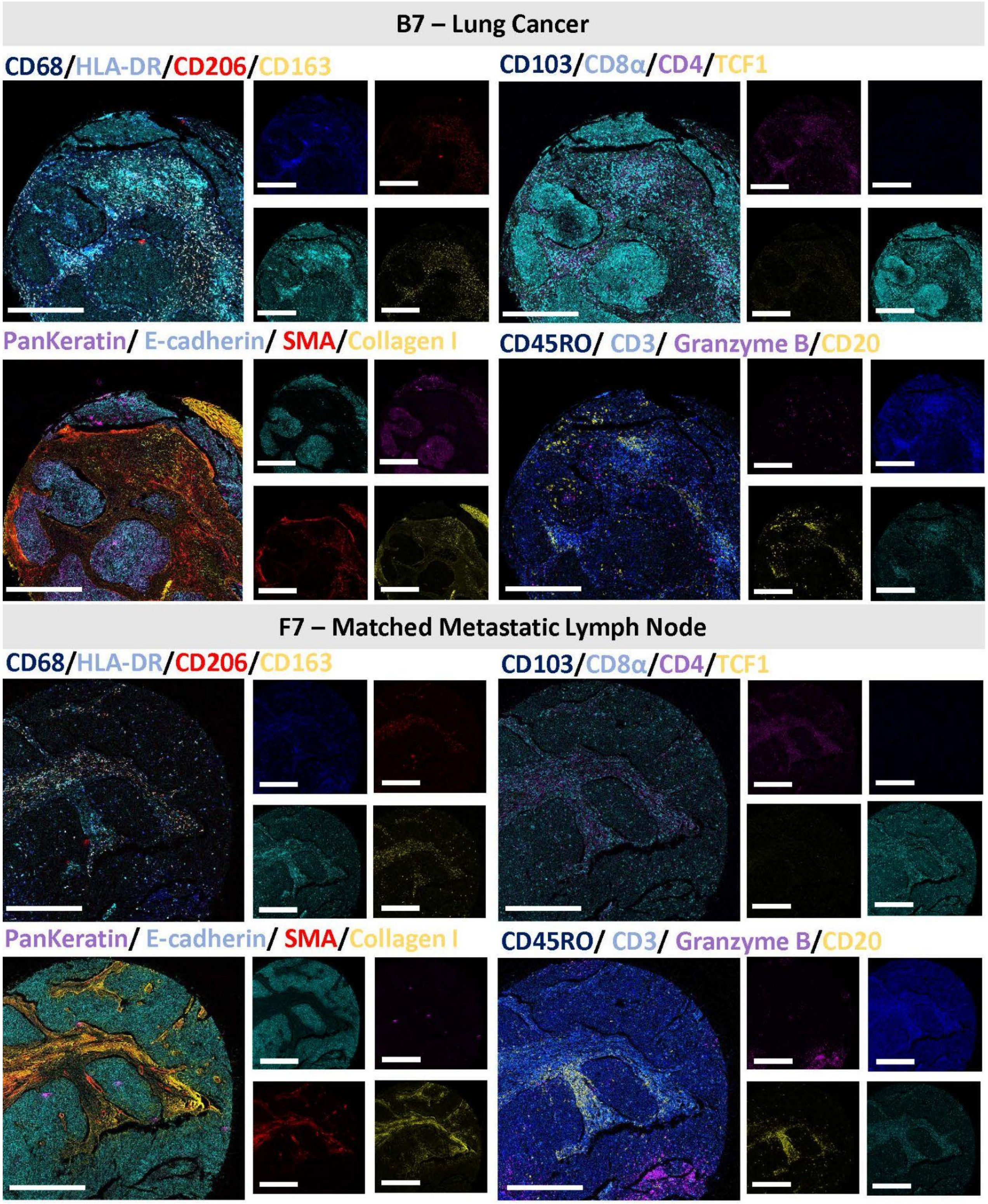

Supplementary Figure 1. Representative IMC images of primary lung cancer sample B7 and its matched metastatic lymph node sample F7. Scale bars represent 500-µm.

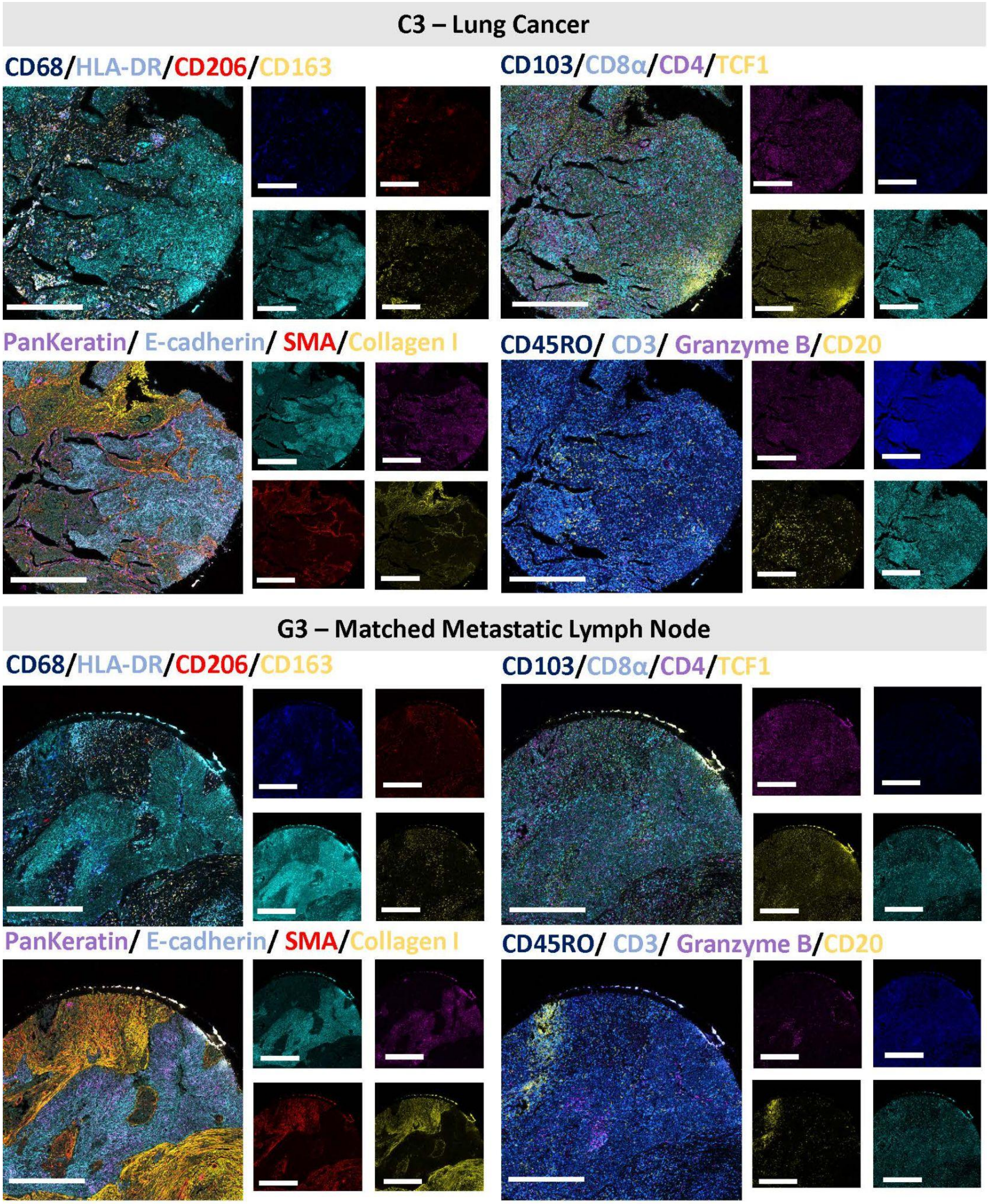
C3 – Lung Cancer
CD68/HLA-DR/CD206/CD163
CD103/CD8α/CD4/TCF1
PanKeratin/ E-cadherin/ SMA/Collagen I
CD45RO/ CD3/ Granzyme B/CD20
G3 – Matched Metastatic Lymph Node
CD68/HLA-DR/CD206/CD163
CD103/CD8α/CD4/TCF1
PanKeratin/ E-cadherin/ SMA/Collagen I
CD45RO/ CD3/ Granzyme B/CD20

Supplementary Figure 2. Representative IMC images of primary lung cancer sample C3 and its matched metastatic lymph node sample G3. Scale bars represent 500-µm.

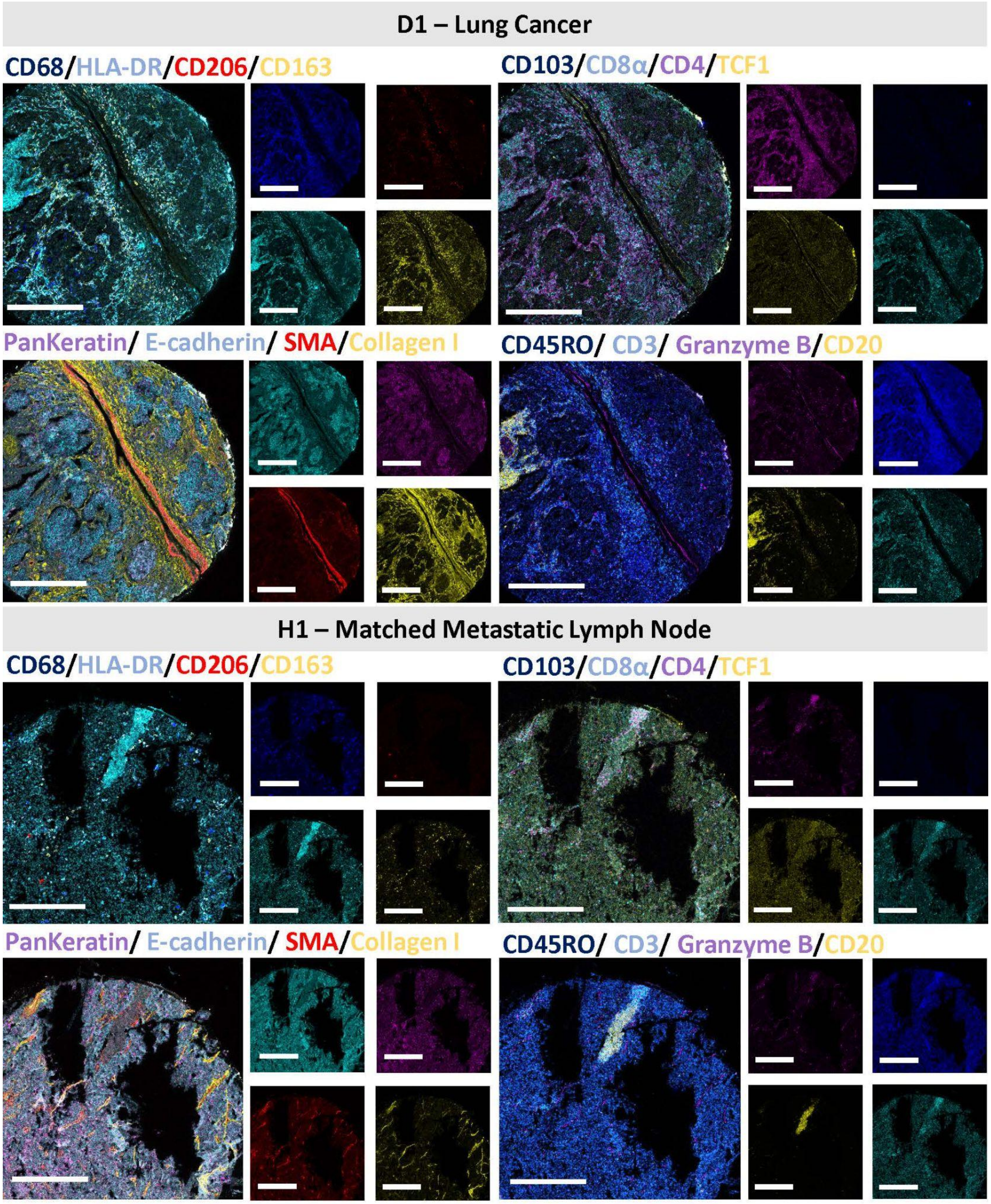
D1 – Lung Cancer
CD68/HLA-DR/CD206/CD163
CD103/CD8α/CD4/TCF1
PanKeratin/ E-cadherin/ SMA/Collagen I
CD45RO/ CD3/ Granzyme B/CD20
H1 – Matched Metastatic Lymph Node
CD68/HLA-DR/CD206/CD163
CD103/CD8α/CD4/TCF1
PanKeratin/ E-cadherin/ SMA/Collagen I
CD45RO/ CD3/ Granzyme B/CD20

Supplementary Figure 3. Representative IMC images of primary lung cancer sample D1 and its matched metastatic lymph node sample H1. Scale bars represent 500-µm.

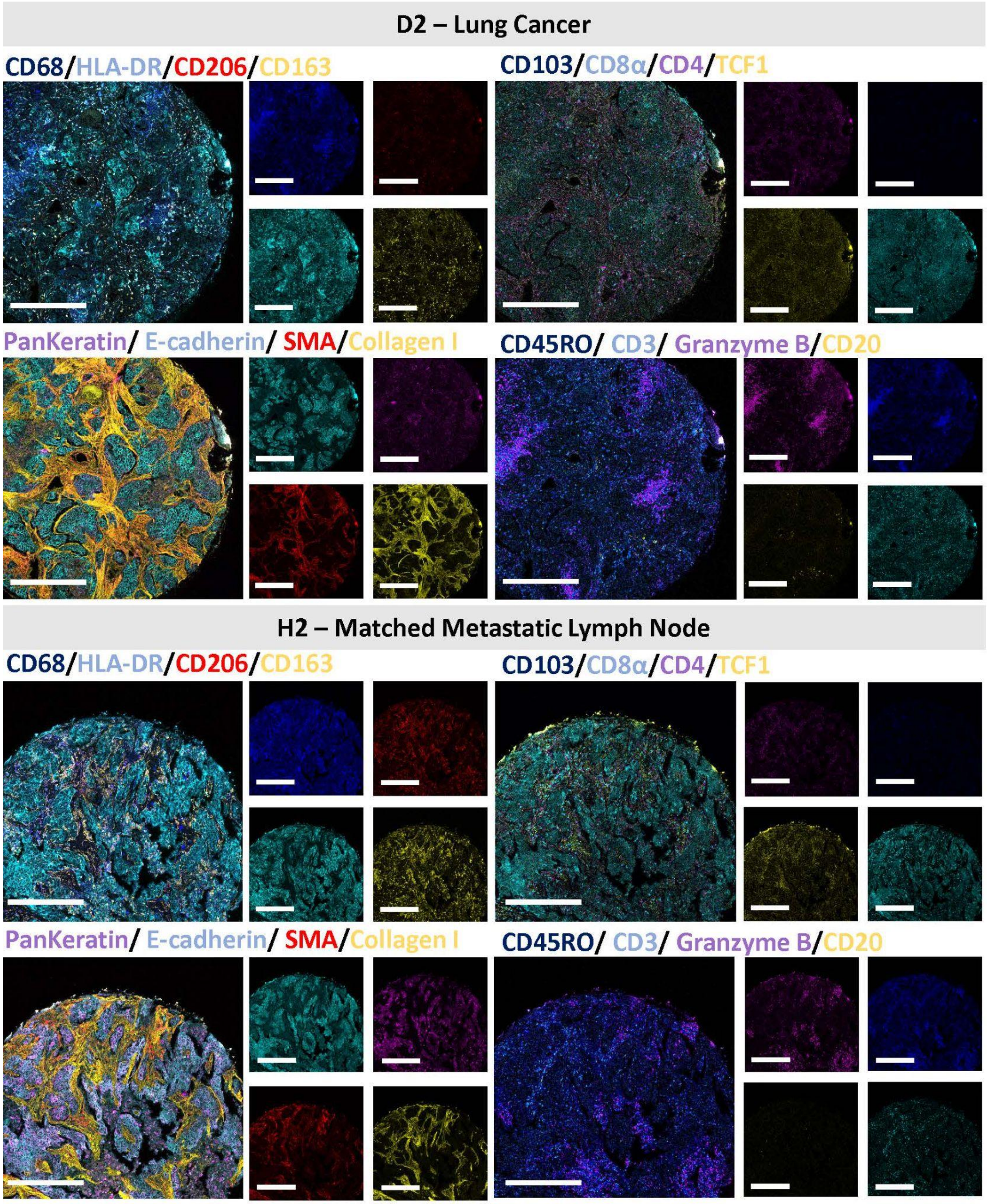
D2 – Lung Cancer
CD68/HLA-DR/CD206/CD163
CD103/CD8α/CD4/TCF1
PanKeratin/ E-cadherin/ SMA/Collagen I
CD45RO/ CD3/ Granzyme B/CD20
H2 – Matched Metastatic Lymph Node
CD68/HLA-DR/CD206/CD163
CD103/CD8α/CD4/TCF1
PanKeratin/ E-cadherin/ SMA/Collagen I
CD45RO/ CD3/ Granzyme B/CD20

Supplementary Figure 4. Representative IMC images of primary lung cancer sample D2 and its matched metastatic lymph node sample H2. Scale bars represent 500-µm.

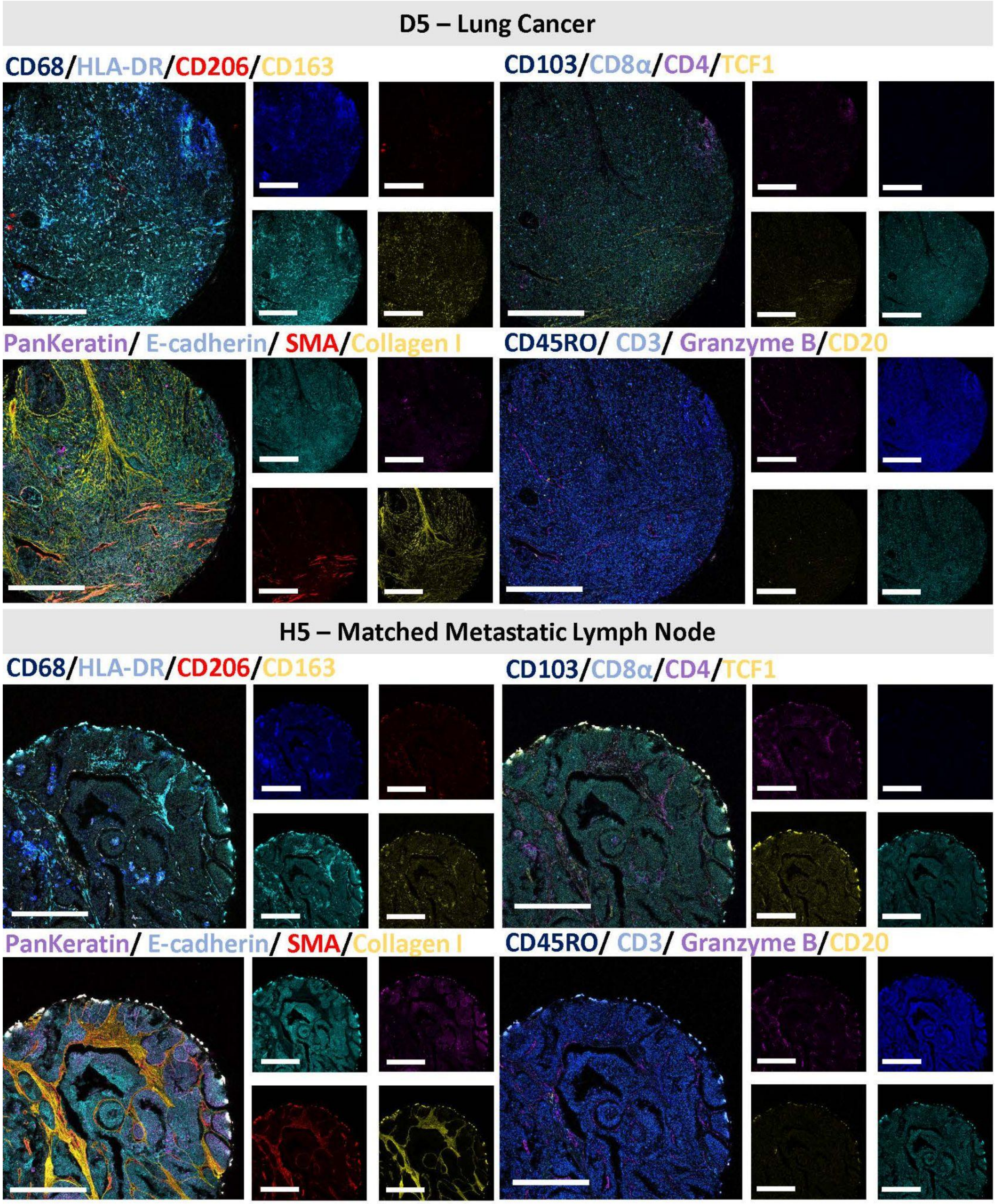
D5 – Lung Cancer
CD68/HLA-DR/CD206/CD163
CD103/CD8α/CD4/TCF1
PanKeratin/ E-cadherin/ SMA/Collagen I
CD45RO/ CD3/ Granzyme B/CD20
H5 – Matched Metastatic Lymph Node
CD68/HLA-DR/CD206/CD163
CD103/CD8α/CD4/TCF1
PanKeratin/ E-cadherin/ SMA/Collagen I
CD45RO/ CD3/ Granzyme B/CD20

Supplementary Figure 5. Representative IMC images of primary lung cancer sample D5 and its matched metastatic lymph node sample H5. Scale bars represent 500-µm.

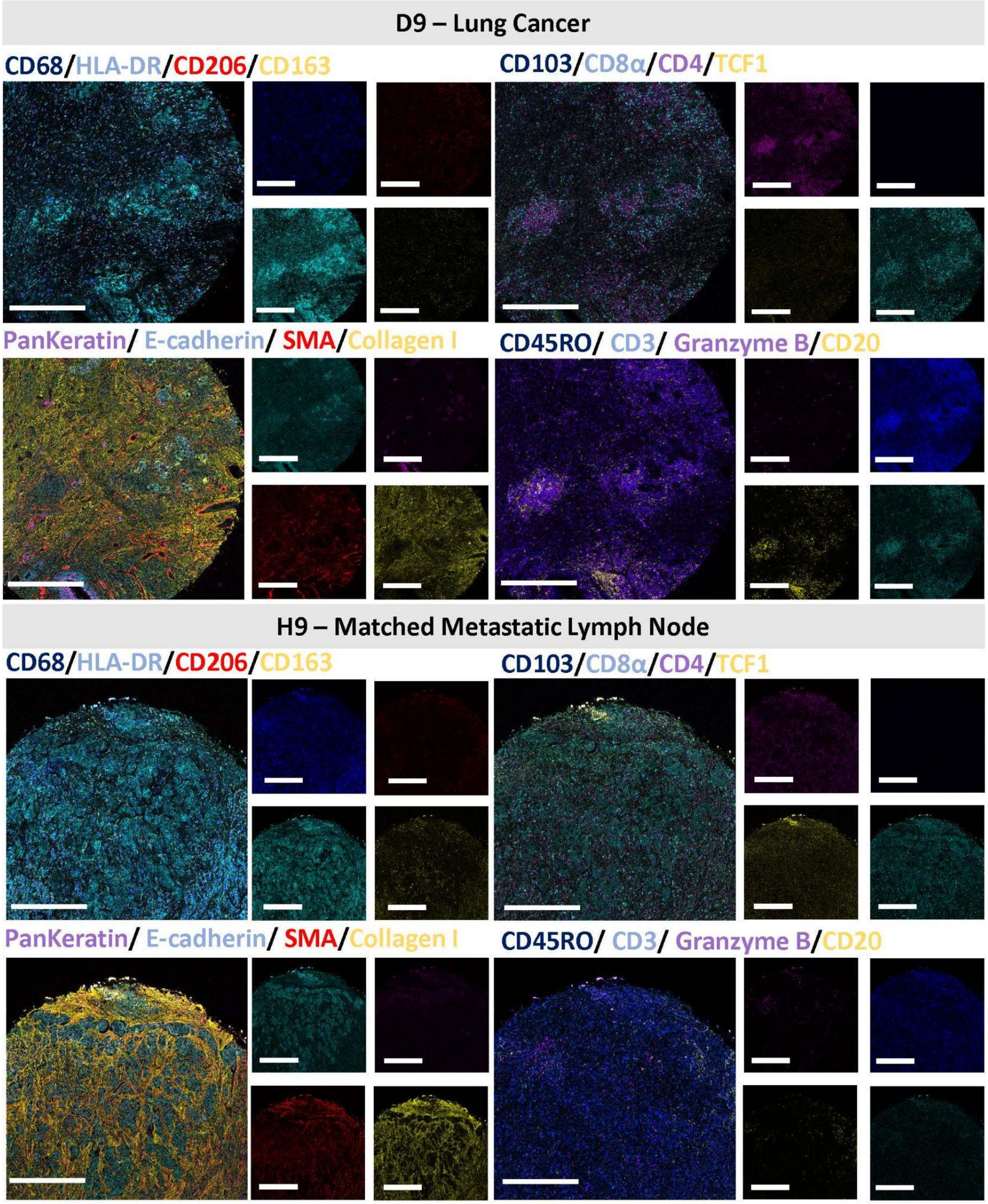
D9 – Lung Cancer
CD68/HLA-DR/CD206/CD163
CD103/CD8α/CD4/TCF1
PanKeratin/ E-cadherin/ SMA/Collagen I
CD45RO/ CD3/ Granzyme B/CD20
H9 – Matched Metastatic Lymph Node
CD68/HLA-DR/CD206/CD163
CD103/CD8α/CD4/TCF1
PanKeratin/ E-cadherin/ SMA/Collagen I
CD45RO/ CD3/ Granzyme B/CD20

Supplementary Figure 6. Representative IMC images of primary lung cancer sample D9 and its matched metastatic lymph node sample H9. Scale bars represent 500-µm.

Lung Cancer Tissues | **Matched Metastatic Lymph Node**

B7

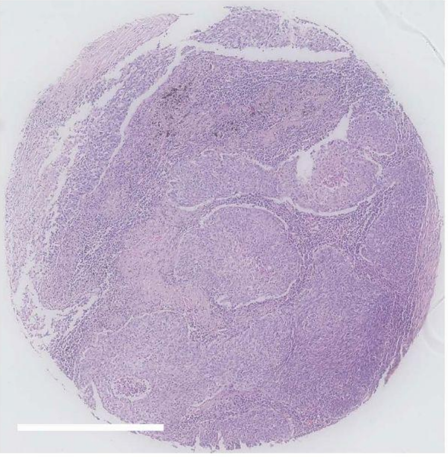

Age: 42
Sex: Male
Pathology diagnosis: SCC
TNM: T3N1M0
Grade: 3
Stage: IIIA
Type: Malignant

F7

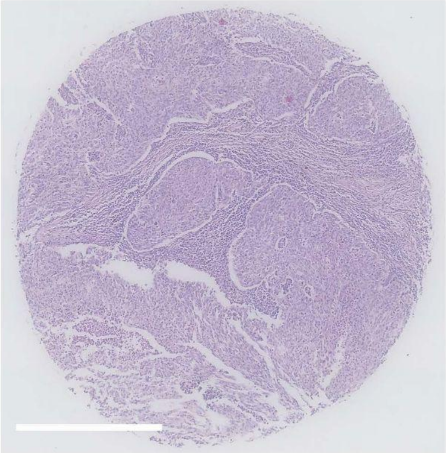

C3

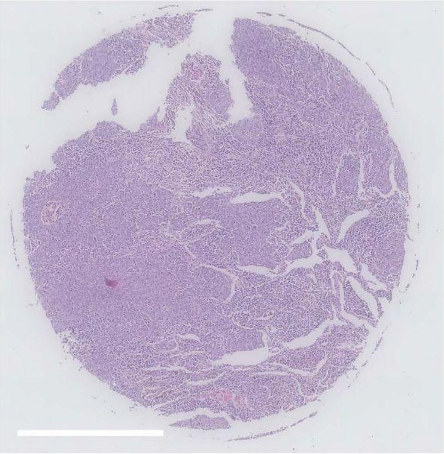

Age: 63
Sex: F
Pathology diagnosis: SCC
TNM: T3N1M0
Grade: 3
Stage: IIIA
Type: Malignant

G3

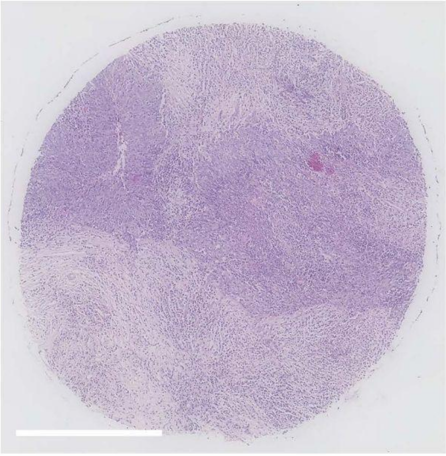

D1

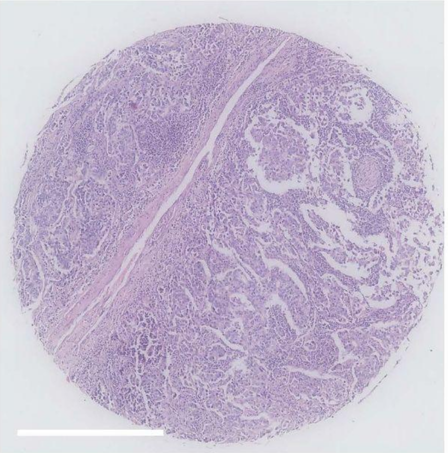

Age: 65
Sex: M
Pathology diagnosis: ADENOCA
TNM: T2N1M0
Grade: 3
Stage: IIIA
Type: Malignant

H1

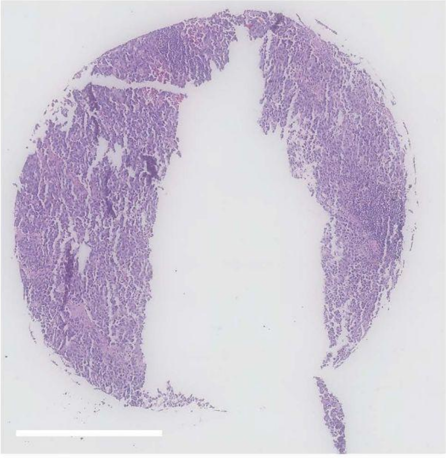

Supplementary Figure 7. Representative H&E images of primary lung cancer samples and their matched metastatic lymph node samples in patients' cohort 1.

*SCC: squamous cell carcinoma, * ADENOCA: adenocarcinoma. Scale bars represent 500-µm.

Lung Cancer Tissues | **Matched Metastatic Lymph Node**

D2

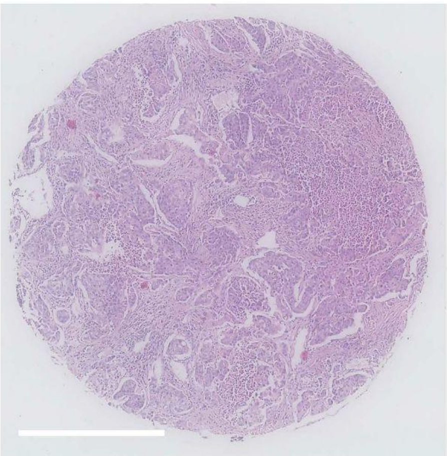

Age: 59
Sex: M
Pathology diagnosis: ADENOCA
TNM: T3N1M0
Grade: 3
Stage: IIIA
Type: Malignant

H2

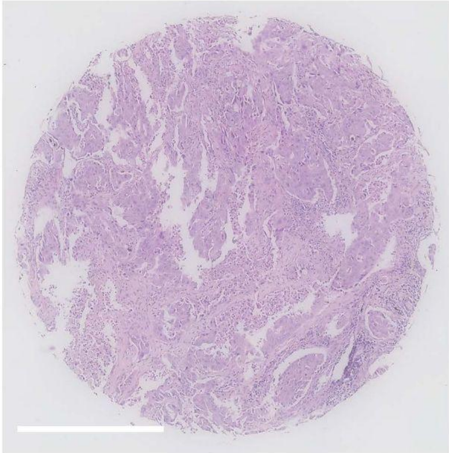

D5

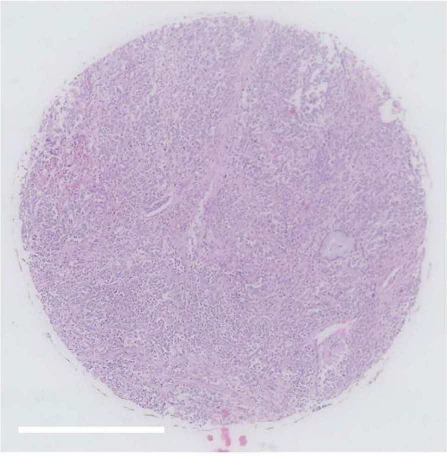

Age: 60
Sex: M
Pathology diagnosis: ADENOCA
TNM: T2N1M0
Grade: 3
Stage: IIA
Type: Malignant

H5

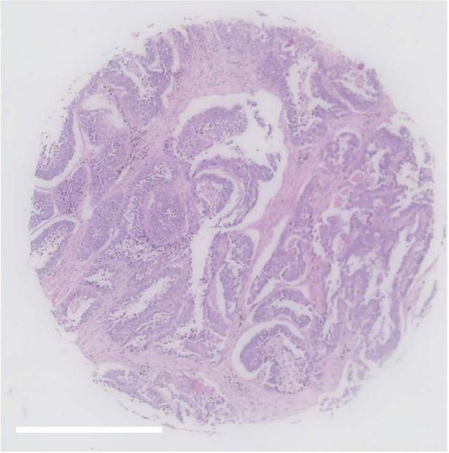

D9

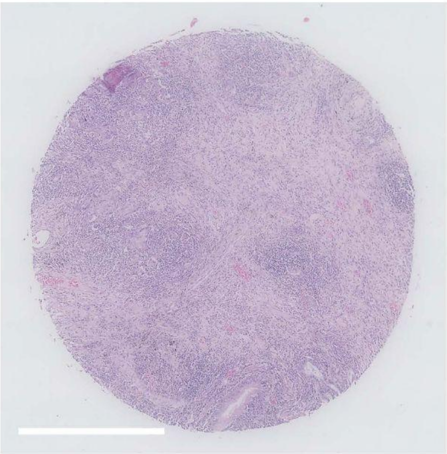

Age: 52
Sex: F
Pathology diagnosis: ADENOCA
TNM: T2N1M0
Grade: 3
Stage: IIA
Type: Malignant

H9

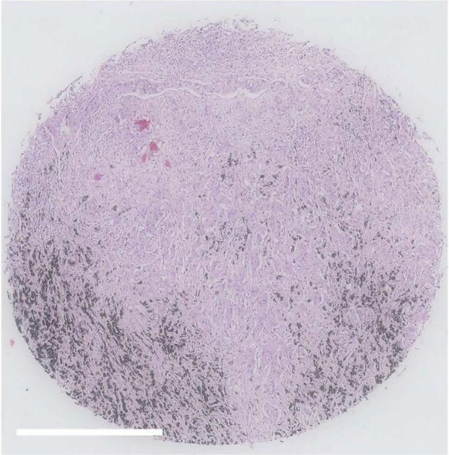

Supplementary Figure 8. Representative H&E images of primary lung cancer samples and their matched metastatic lymph node samples in patients' cohort 1.

*SCC: squamous cell carcinoma, * ADENOCA: adenocarcinoma. Scale bars represent 500-μm.

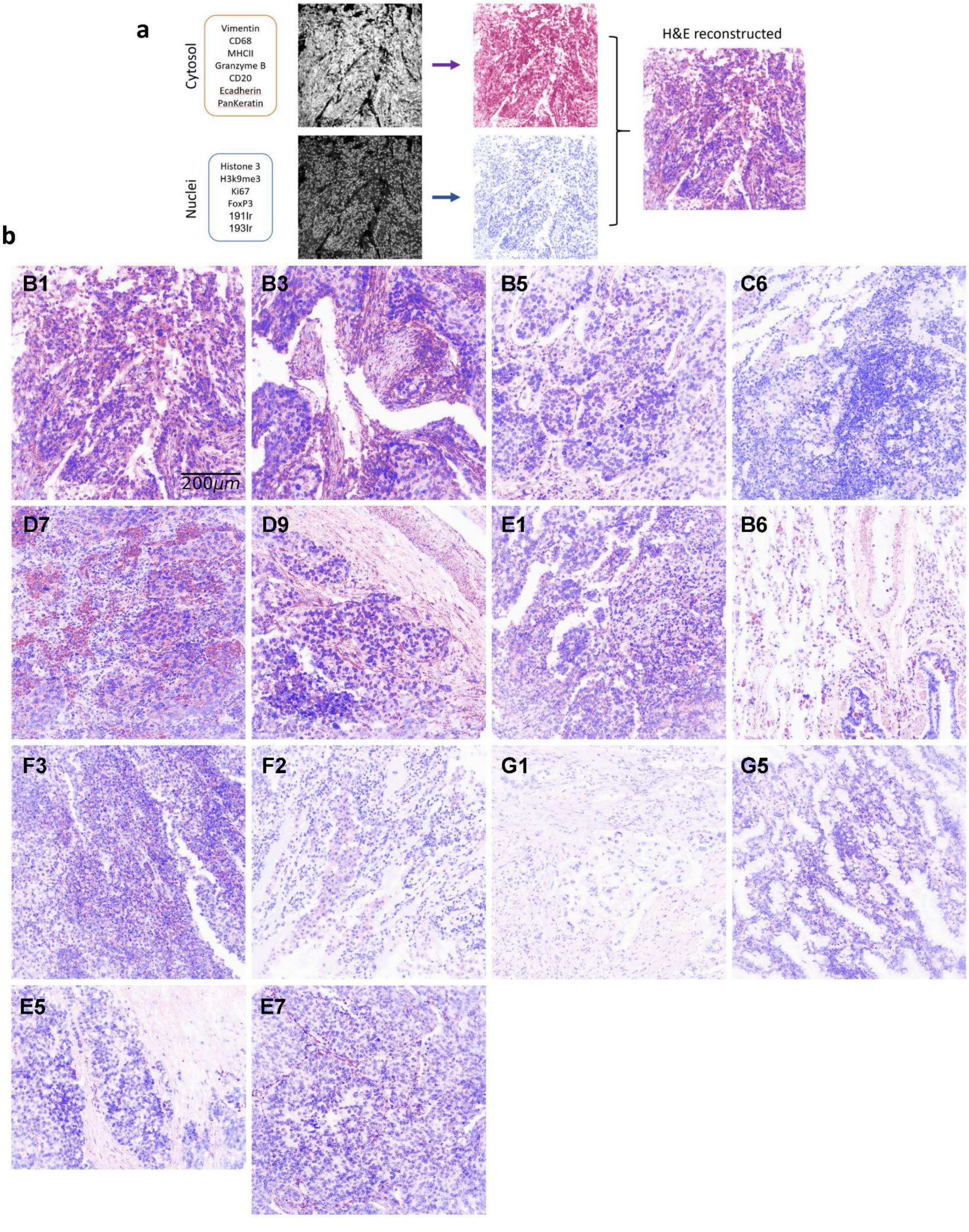
a
Cytosol
Vimentin
CD68
MHCII
Granzyme B
CD20
Ecadherin
PanKeratin
Nuclei
Histone 3
H3k9me3
Ki67
FoxP3
191Ir
193Ir
H&E reconstructed
b
B1
200μm
B3
B5
C6
D7
D9
E1
B6
F3
F2
G1
G5
E5
E7

Supplementary Figure 9. Virtual H&E images of the patients' cohort 2 using imaging mass cytometry data. Scale bars represent 200-µm.

- **a.** Multiple markers were used to identify the cytosol (Vimentin, CD68, MHC-II, Granzyme B, CD20, E-cadherin, Pankeratin) and the nucleus (Histone 3, H3K9me3, Ki67, FoxP3, Intercalator 191Ir/193Ir). The cytosol was presented in a purple shade whereas the nuclei were presented in a blue shade.
- **b.** Virtual H&E images of the patients' (n=14) lung cancer samples were illustrated.

Marker Intensity Variation with the Cell-based Segmentation on UMAP

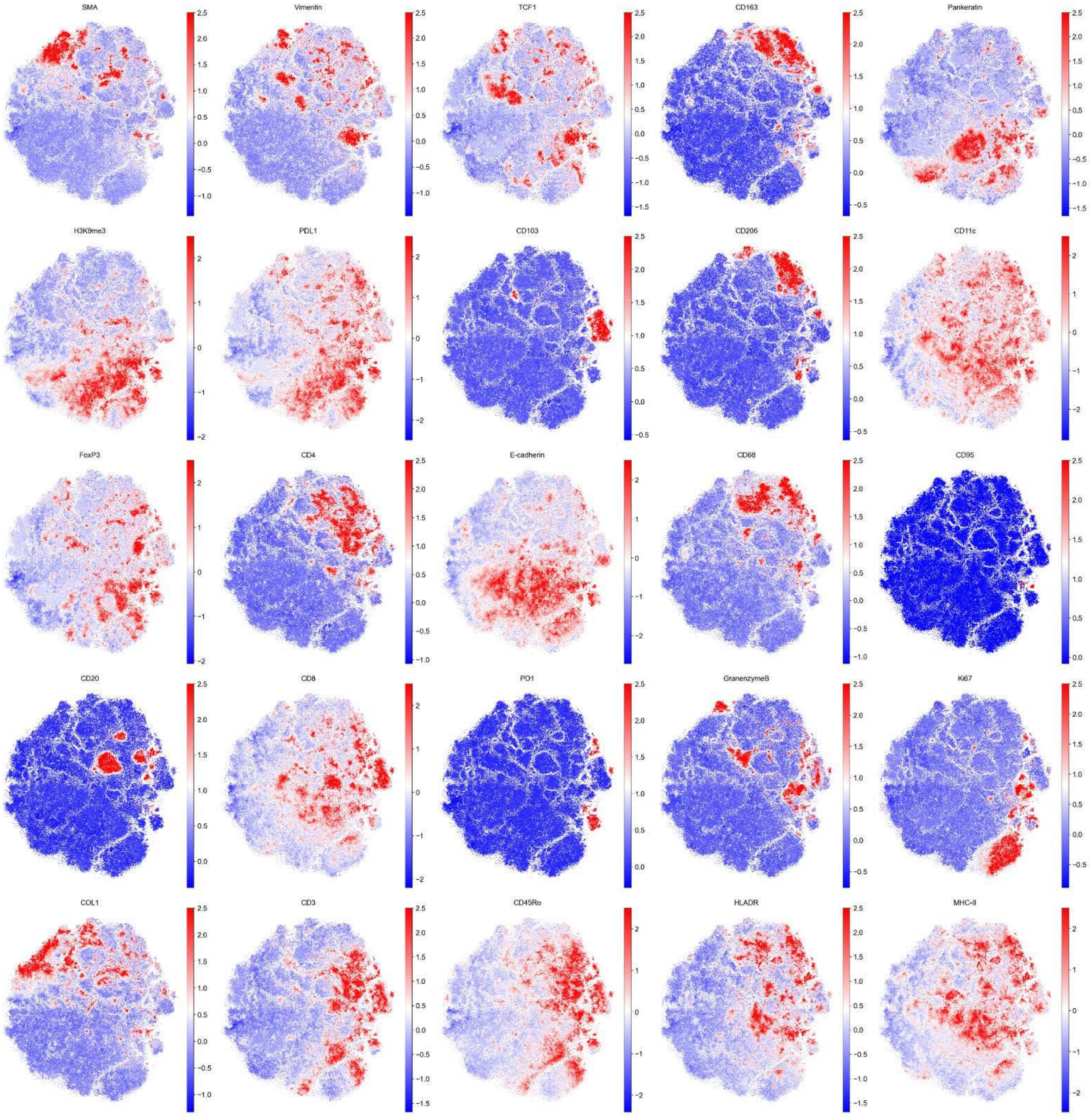

Supplementary Figure 10. Variation in the mean intensity of single cell markers across all samples in cohort 1 was displayed on t-distributed stochastic neighbor embedding (t-SNE) embedding. Red indicated a high expression level and blue demonstrated a low expression level at the single-cell level (Number of patient samples = 12 and n = 264,191 cells).

Combined Markers

Tumor/ Stroma/ CD8α/ M1/ M2

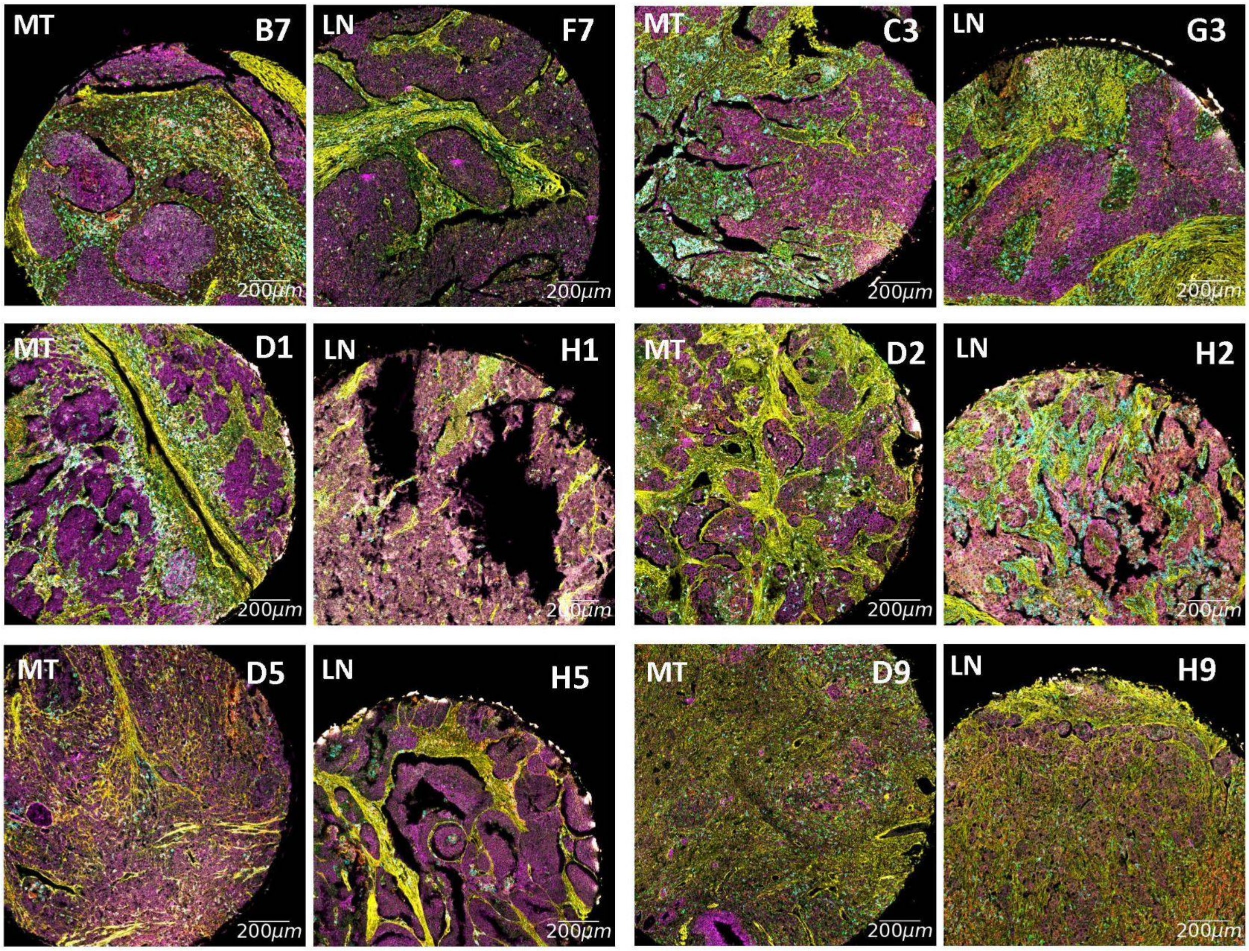

Supplementary Figure 11. Representative tissue images of cohort 1 patients' primary lung cancer samples and their matched metastatic lymph nodes (B7-F7, C3-G3, D1-H1, D2-H2, D5-H5, D9-H9) were shown by combining tumor region (purple), stroma region (yellow), CD8α (green), M1 (red), and M2 (cyan). Scale bars represent 200-μm.

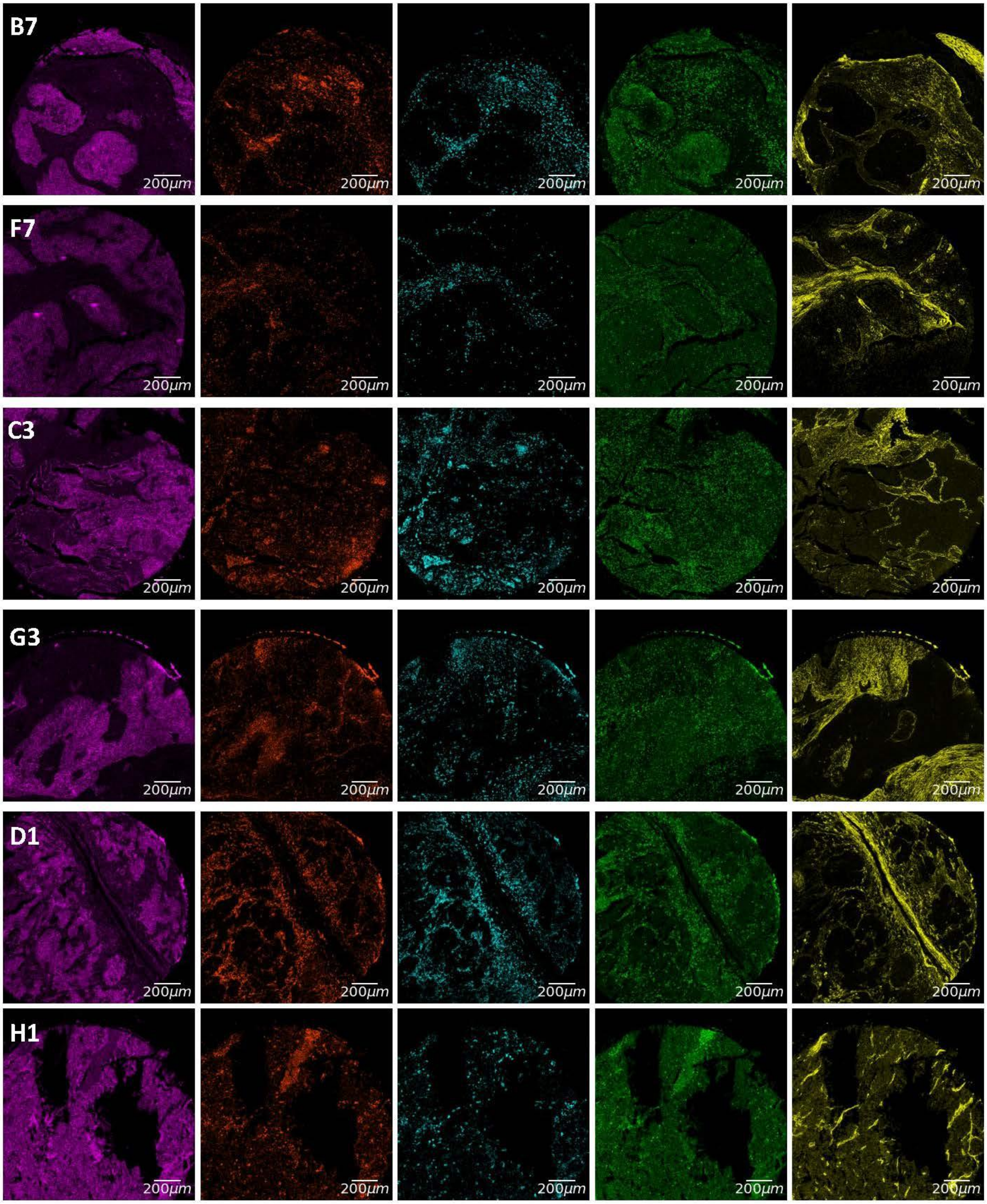
B7
F7
C3
G3
D1
H1
200μm

Supplementary Figure 12. Representative tissue images of cohort 1 patients' primary lung cancer samples and their matched metastatic lymph nodes including B7/F7, C3/G3, and D1/H1 were presented. Tissue regions were marked as tumor region (purple), stroma region (yellow), CD8α (green), M1 (red), and M2 (cyan). Scale bars represent 200-µm.

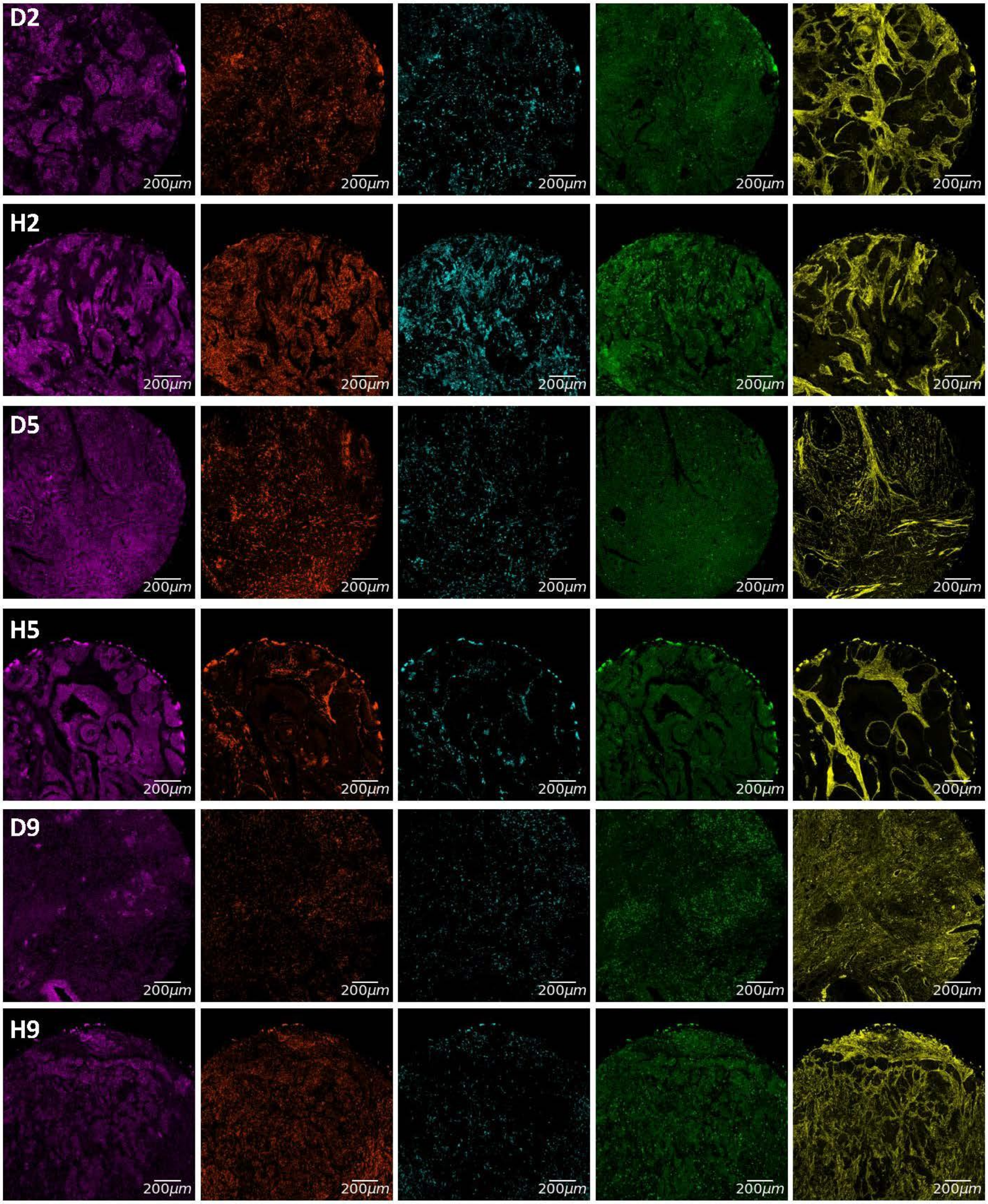

D2
H2
D5
H5
D9
H9
200μm

Supplementary Figure 13. Representative tissue images of cohort 1 patients' primary lung cancer samples and their matched metastatic lymph nodes including D2/H2, D5/H5, and D9/H9 were provided. Individual anatomical regions were marked as tumor region (purple), stroma region (yellow), CD8α (green), M1 (red), and M2 (cyan). Scale bars represent 200-μm.

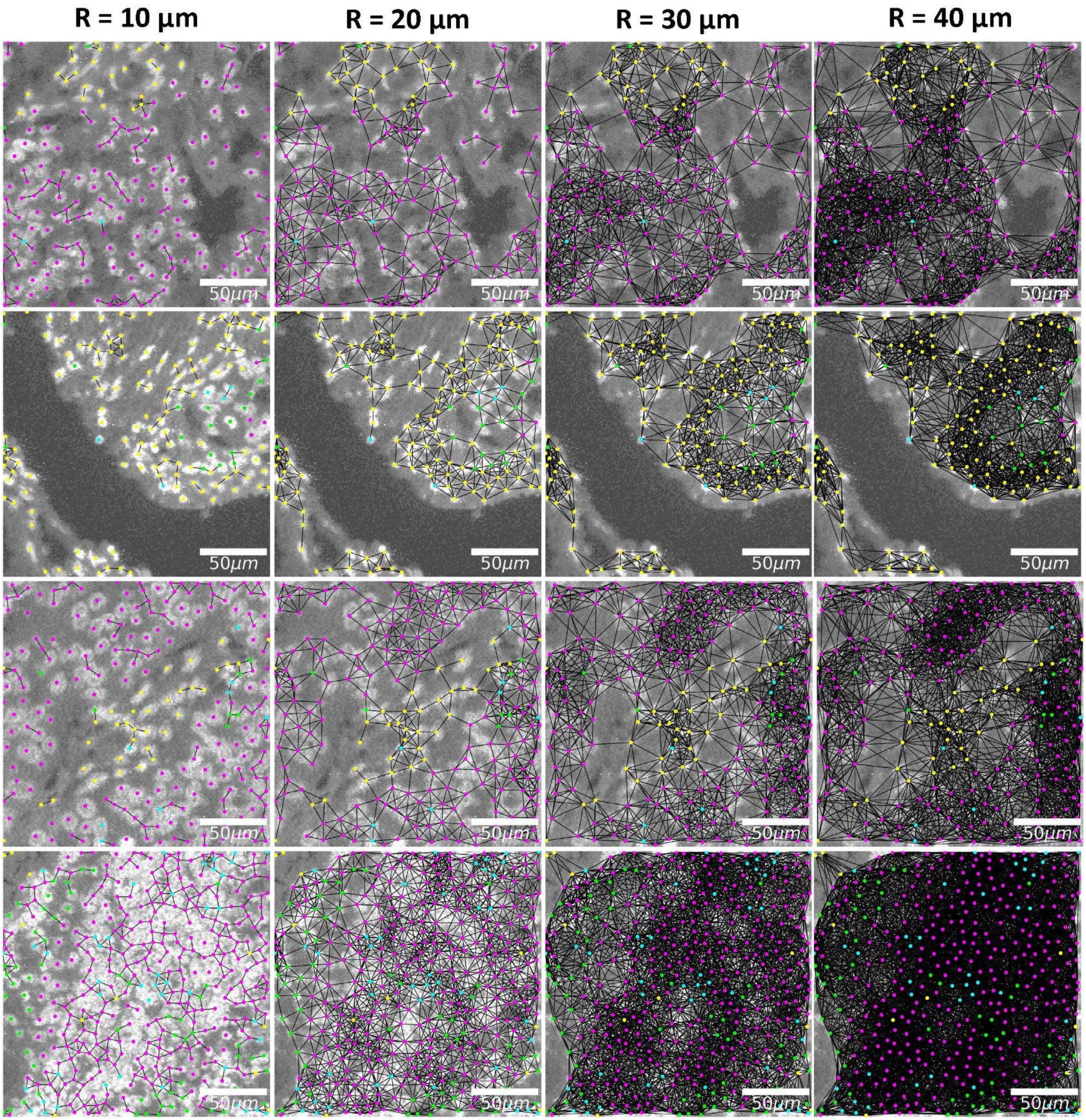
R = 10 μm
R = 20 μm
R = 30 μm
R = 40 μm
50μm

Supplementary Figure 14. Comparisons of spatial cell neighborhood graphs constructed at different pixel distances radius between centroids were illustrated. A radius of 10-µm was used for the far left column to connect the cell centroids, 20-µm for the center-left panel, 30-µm for the center-right panel, and 40-µm for the right panel. The optimal radius was chosen empirically with the radius such that the constructed graph captured cell neighboring information at the best. Scale bars represent 50-µm.

Marker Intensity Variation with the Pixel-based Segmentation on UMAP

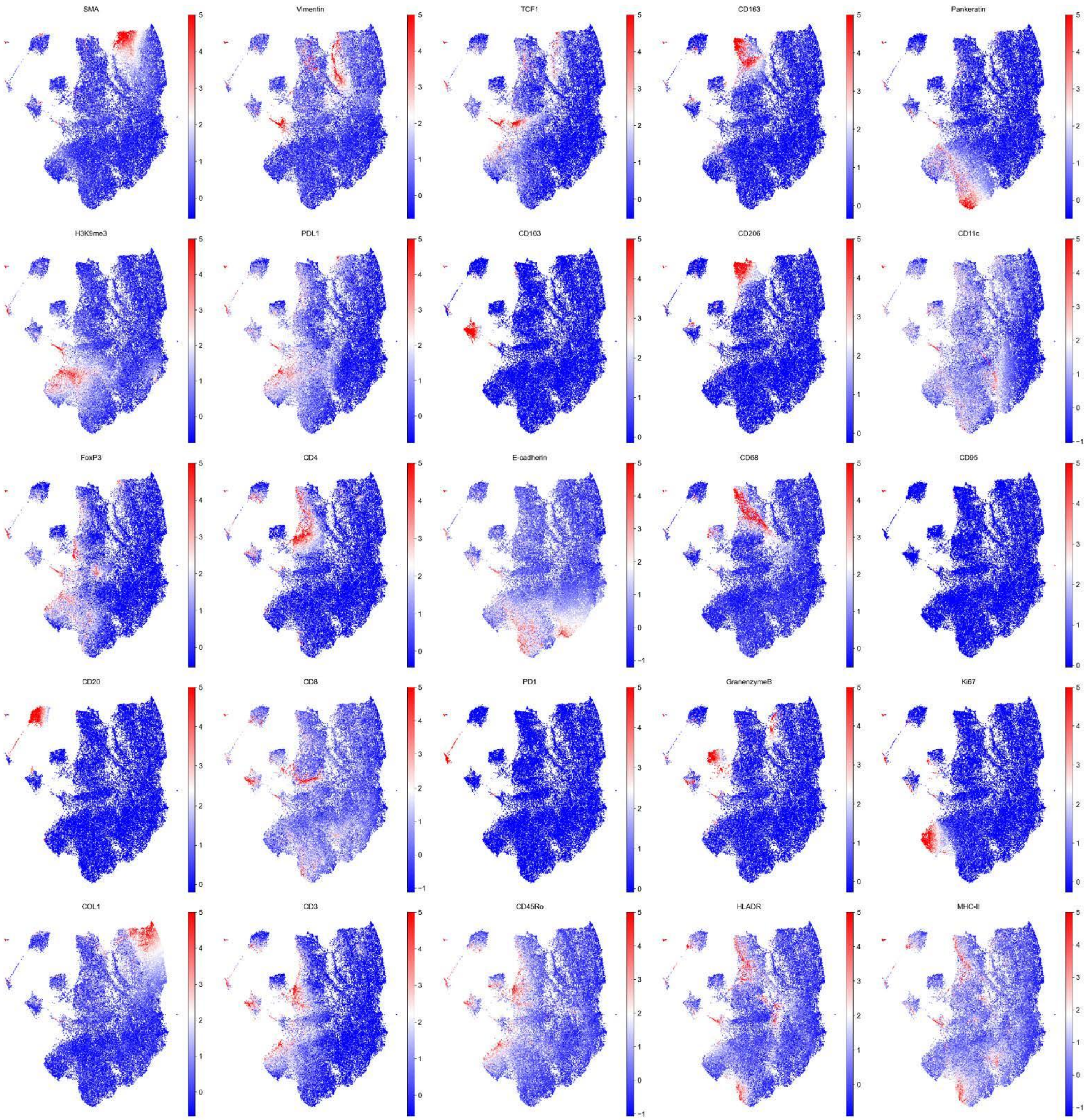

Supplementary Figure 15. The intensity variation of pixel-level markers across all samples in cohort 1 using the Uniform Manifold Approximation and Projection (UMAP) embedding (n=5,093,784 pixels).

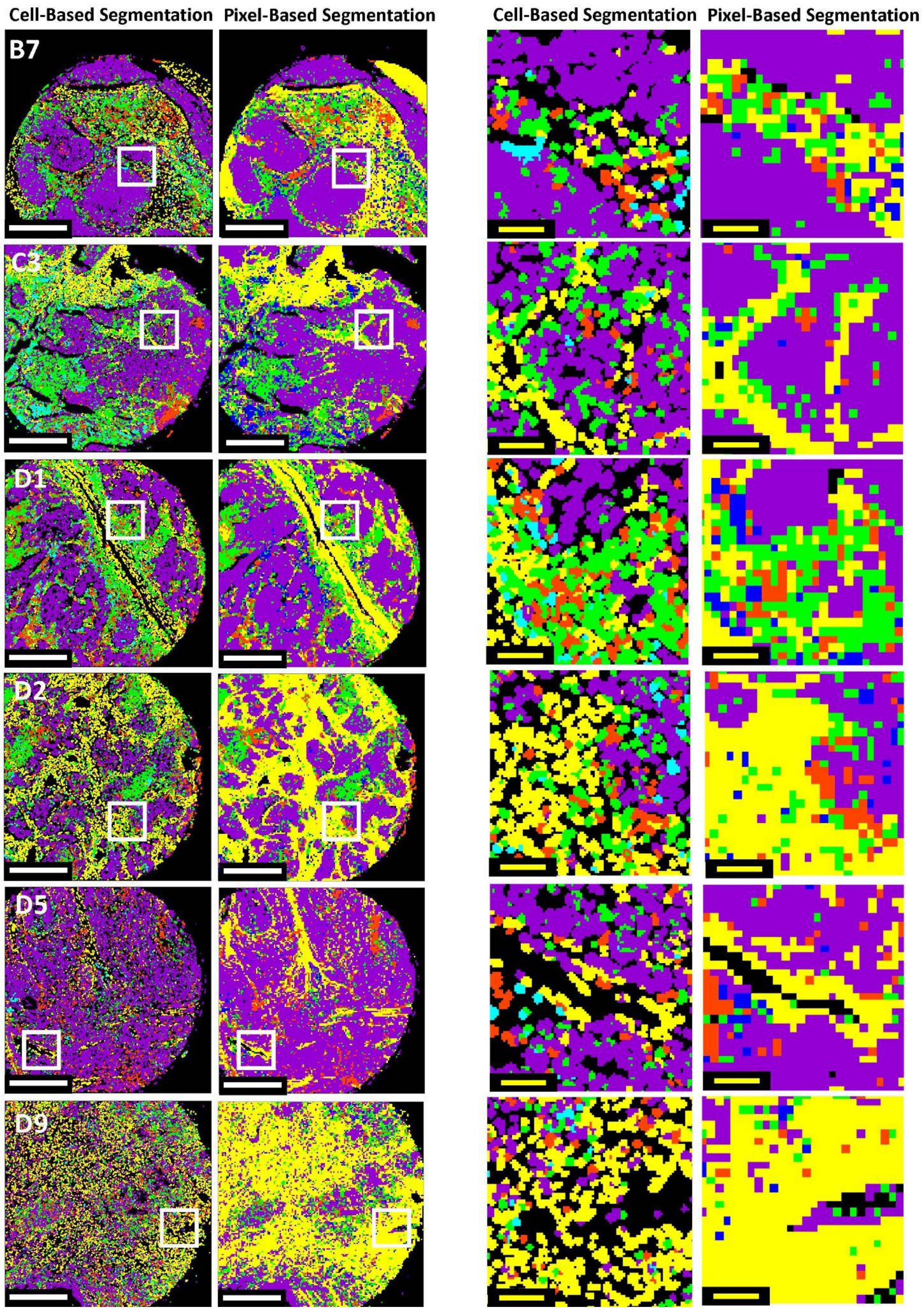
Cell-Based Segmentation
Pixel-Based Segmentation
Cell-Based Segmentation
Pixel-Based Segmentation
B7
C3
D1
D2
D5
D9

Supplementary Figure 16. Pixel-level immune microenvironment classification outperforms the cell-level segmentation in identifying stromal and dense tumor regions. Comparisons of cell-level and pixel-level immune microenvironment classification were shown with zoomed-in examples per region (right). White Scale bars represent 500-µm and yellow scale bars represent 50-µm.

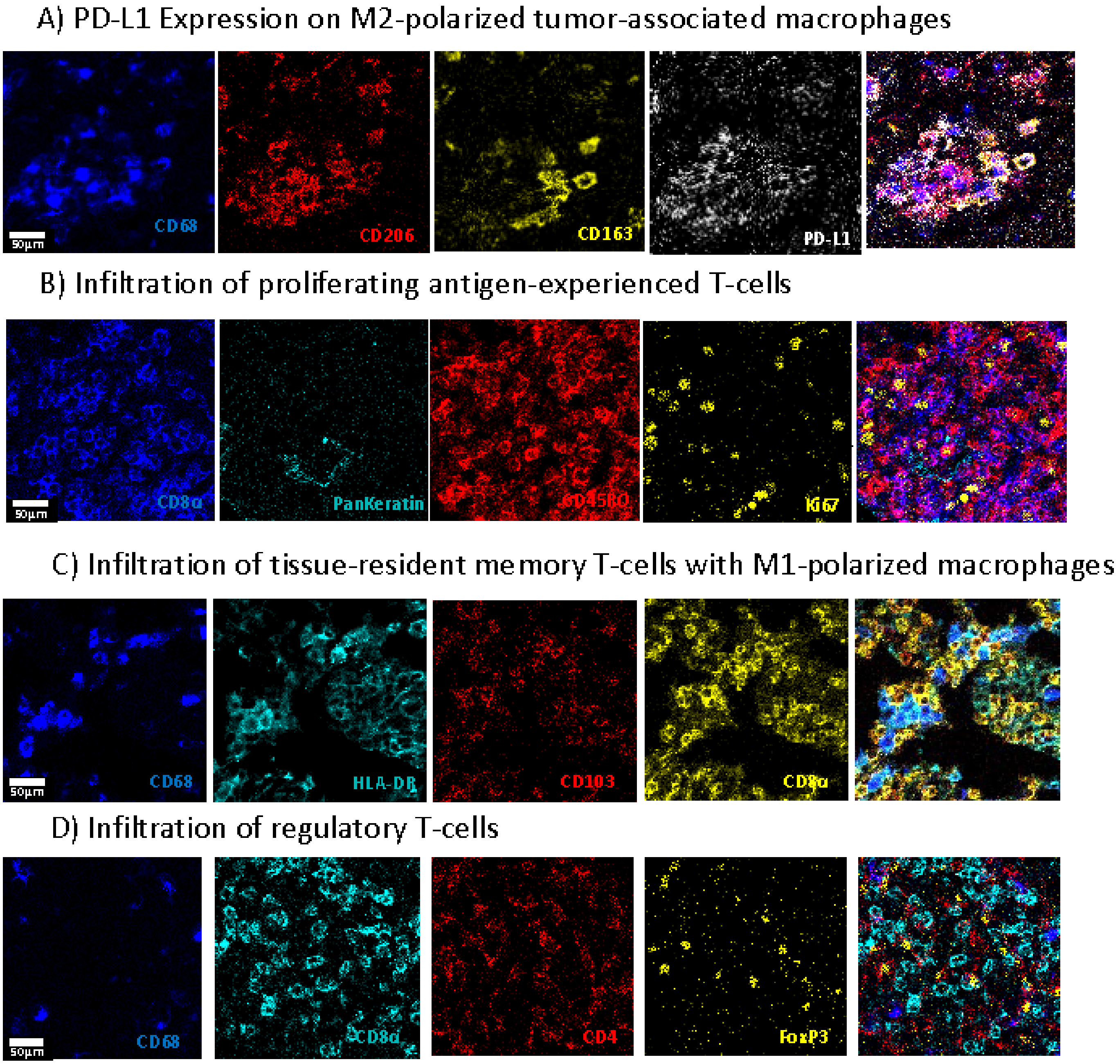
A) PD-L1 Expression on M2-polarized tumor-associated macrophages
50µm
CD68
CD206
CD163
PD-L1
B) Infiltration of proliferating antigen-experienced T-cells
50µm
CD8α
PanKeratin
CD45RO
Ki67
C) Infiltration of tissue-resident memory T-cells with M1-polarized macrophages
50µm
CD68
HLA-DR
CD103
CD8α
D) Infiltration of regulatory T-cells
50µm
CD68
CD8α
CD4
FoxP3

Supplementary Figure 17. Visual representation of immune cellular phenotypes included PD-L1+ M2 tumor-associated macrophages, antigen-experienced T-cells, tissue-resident memory T-cells, and regulatory T-cells using imaging mass cytometry raw data. Scale bars represent 50-µm.

a PD-L1+ M2 macrophages were shown with the expression of CD68 (blue), CD206 (red), CD163 (yellow), and PD-L1 (white).

b Proliferating antigen-experiences T-cells were demonstrated with the expression of CD8α (blue), CD45RO (red), and Ki67 (yellow).

c Tissue-resident memory T-cells were indicated with the expression of CD8α (yellow) and CD103 (red).

d Regulatory T-cells were presented with the expression of CD4 (red) and FoxP3 (yellow).

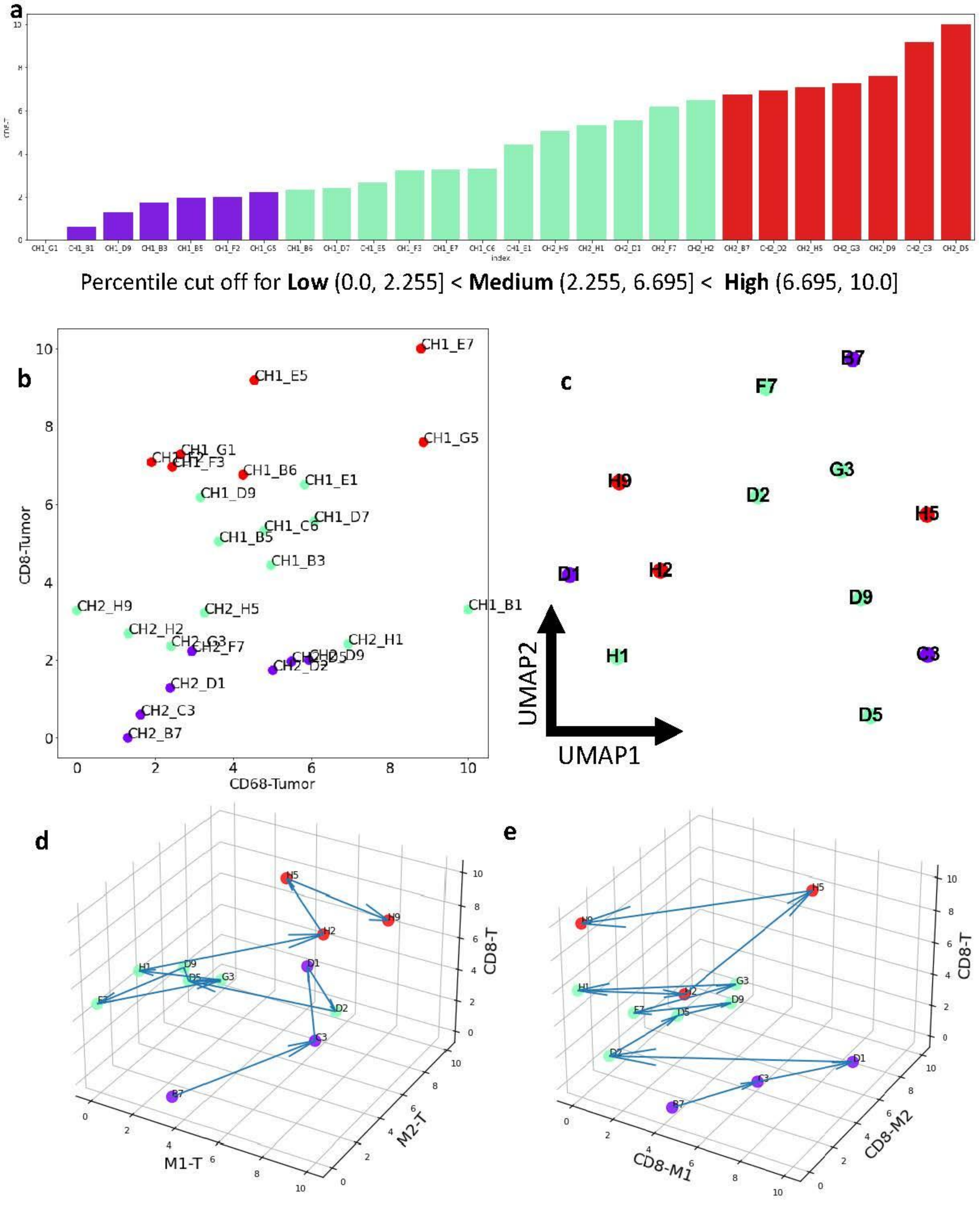
a
Percentile cut off for **Low** (0.0, 2.255] < **Medium** (2.255, 6.695] < **High** (6.695, 10.0]
b
CD8-Tumor
CD68-Tumor
CH1_E7
CH1_E5
CH1_G5
CH1_G1
CH1_F3
CH1_B6
CH1_E1
CH1_D9
CH1_D7
CH1_C6
CH1_B5
CH1_B3
CH2_H9
CH2_H5
CH1_B1
CH2_H2
CH2_G3
CH2_F7
CH2_H1
CH2_D2
CH2_D1
CH2_C3
CH2_B7
c
B7
F7
G3
H9
D2
H5
D1
H2
D9
H1
D5
UMAP2
UMAP1
d
M1-T
M2-T
CD8-T
e
CD8-M1
CD8-M2
CD8-T

Supplementary Figure 18. High-dimensional patient stratification for the immune infiltration.

a Weighted CD8-T infiltration scores within the two patients' cohorts were shown using a bar plot. CH1 refers to cohort 1 and CH2 refers to cohort 2. 75th percentile scores were considered high (Red) and 25th percentile scores were considered low (Purple). The remaining scores were considered intermediate (Green).

b 2D scatter plot of the CD68-T vs. CD8-T distribution for the two patients' cohorts based on their immune infiltration scores (low in purple, intermediate in green, and high in red) was demonstrated.

c UMAP provided the distribution of the patients within the new patients' cohort based on their SpatialVizScore for all possible interactions (T-CD8, T-M1, T-M2, etc).

d-e 3D scatter plot of the CD8-T, M1-T, and M2-T distribution for the patients within the second cohort of patients was presented. The arrows indicated the ascending distribution of CD8-T scores.

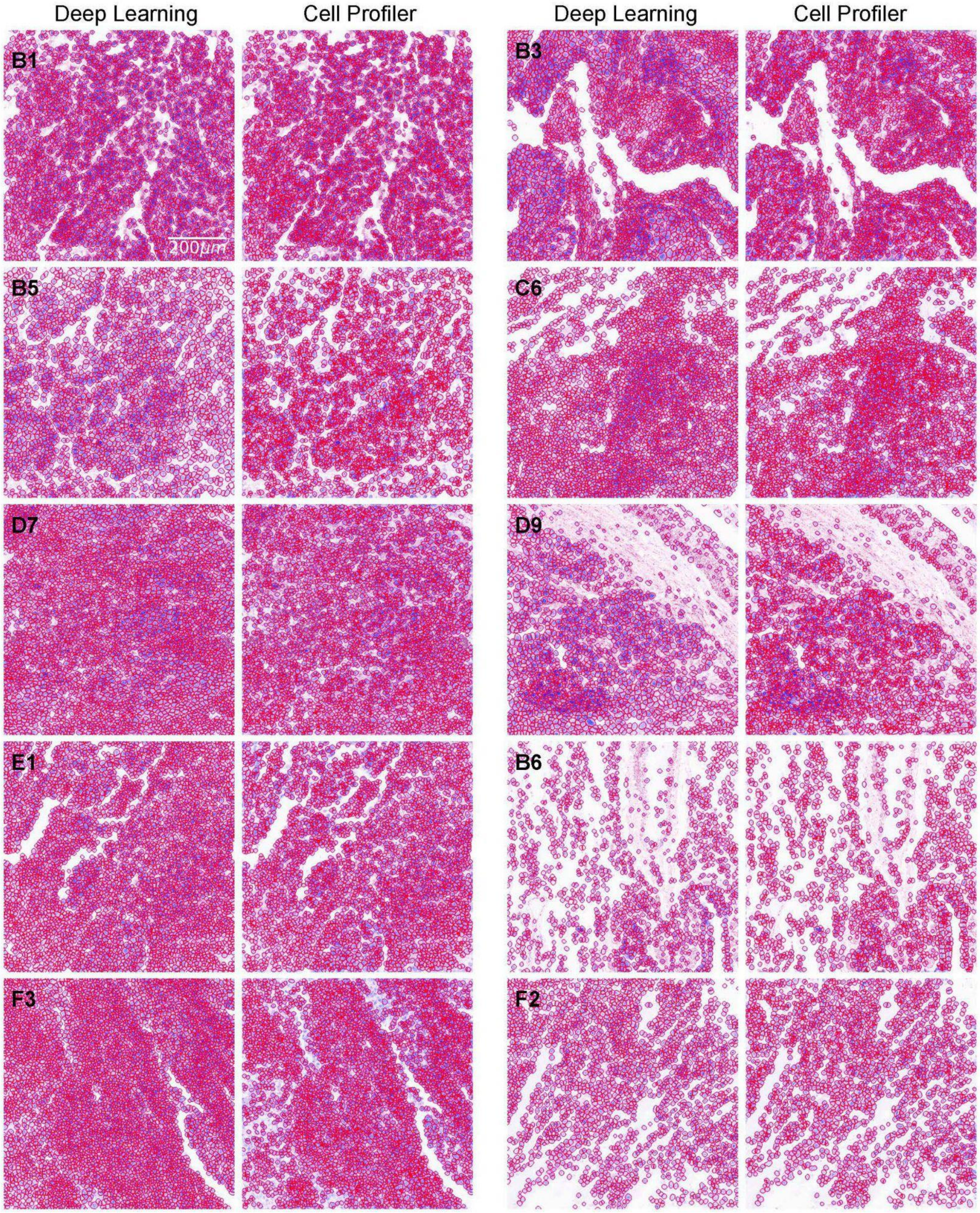
Deep Learning
Cell Profiler
Deep Learning
Cell Profiler
B1
200μm
B3
B5
C6
D7
D9
E1
B6
F3
F2

Supplementary Figure 19. Comparisons of single-cell segmentation techniques using a deep-learning method (CellPose) and open-source software (CellProfiler) were presented. Segmentation results were overlaid on virtual H&E for samples in cohort 2. Single-cell segmentation results from both methods were similar in terms of performance. Scale bars represent 200-μm.

Primary Lung Cancer ROIs

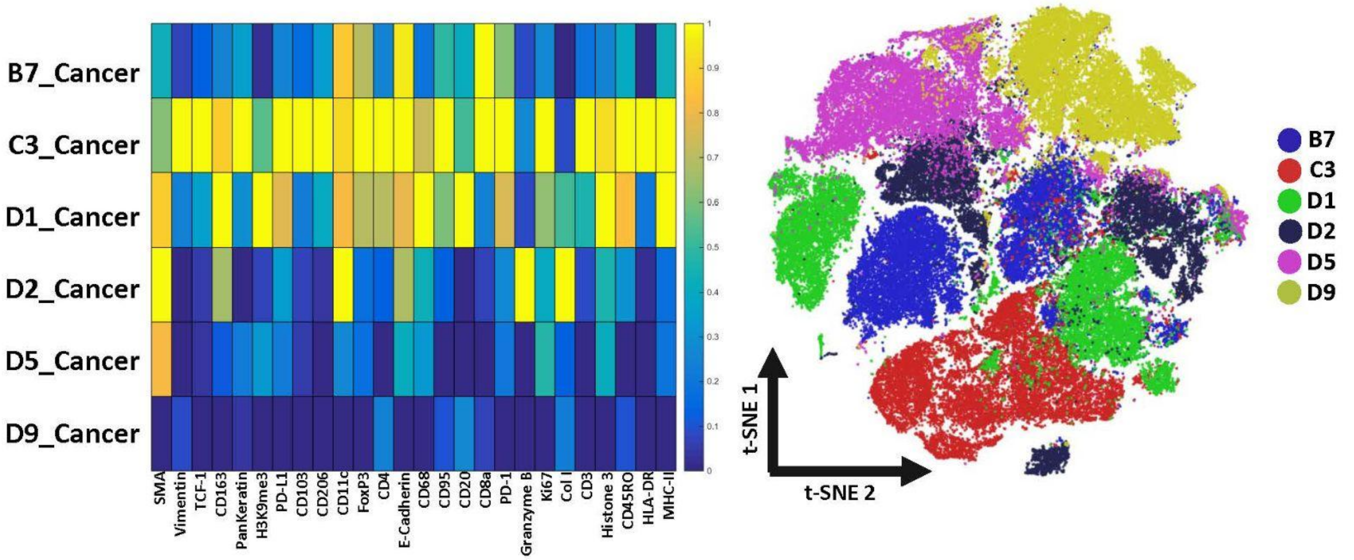

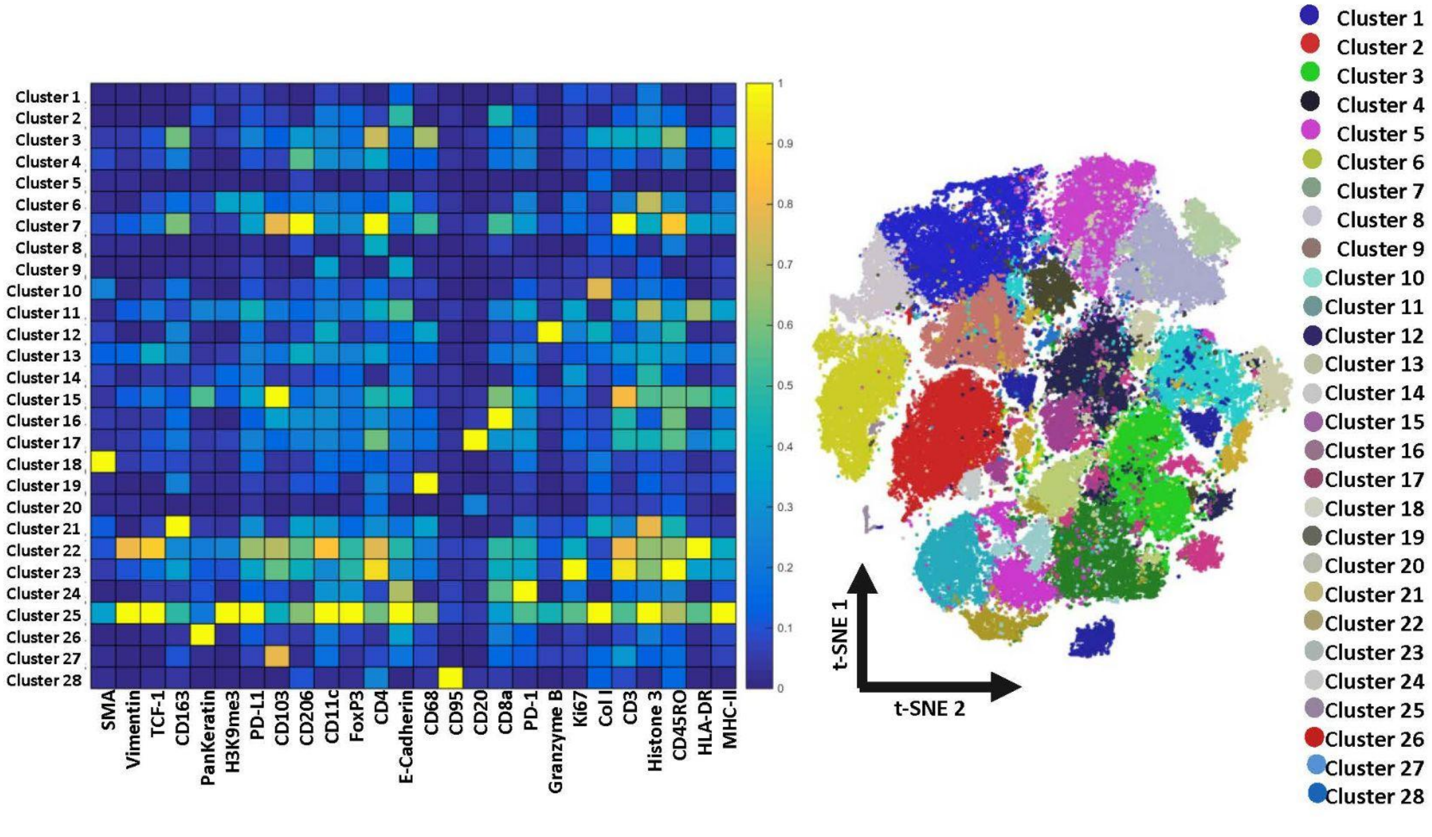

Supplementary Figure 20. Benchmarking SpatialVizScore against HistoCAT was presented. The top row demonstrated the marker expression profile for regions of interest (ROIs) in cohort 1 patients and their distribution on a t-SNE plot. The bottom row provided the results from the HistoCAT's unsupervised clustering with the cluster marker expression and their distribution on a t-SNE plot.

Matched Metastatic Lymph Node ROIs

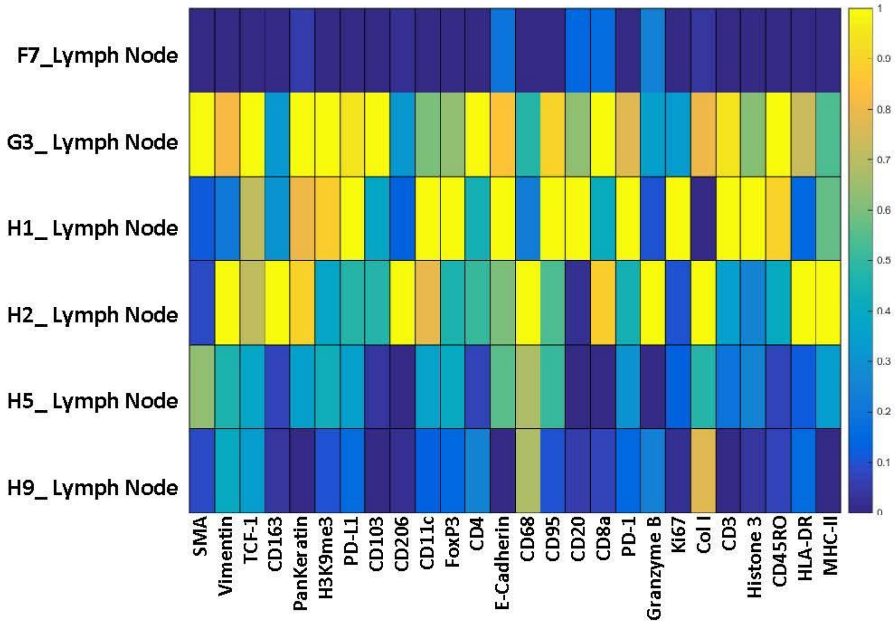

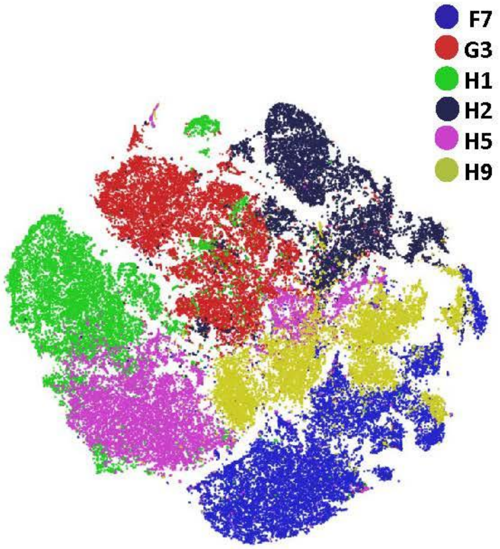

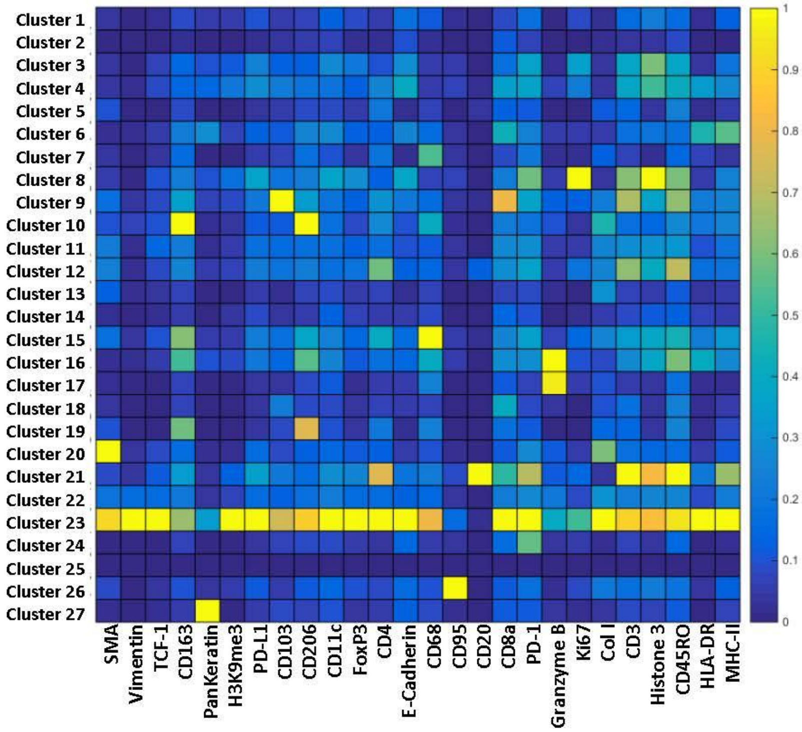

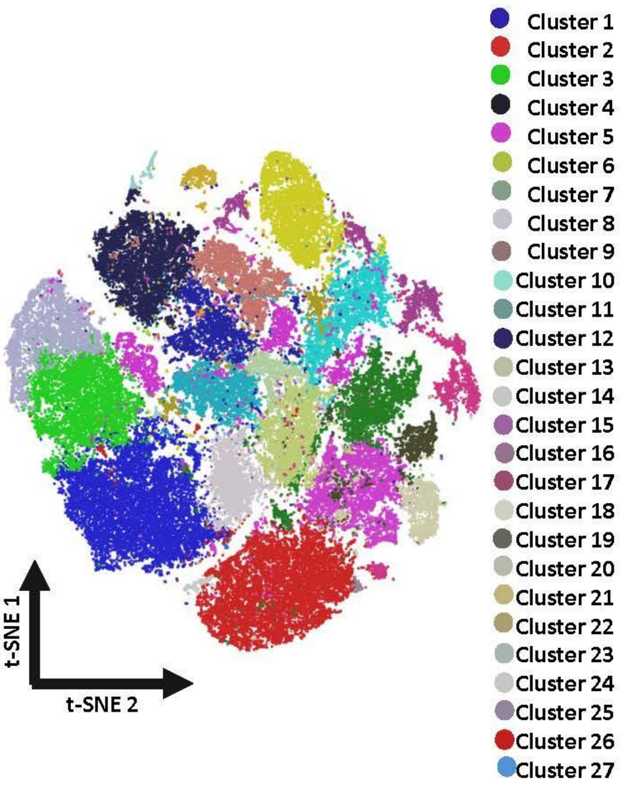

Supplementary Figure 21. Benchmarking SpatialVizScore against HistoCAT was shown. The top row indicated expression profiles of the marker for ROIs in lymph nodes of cohort 1 patients and their distribution on a t-SNE plot. The bottom row demonstrates the results from the HistoCAT's unsupervised clustering with the cluster marker expression and their distribution on a t-SNE plot.

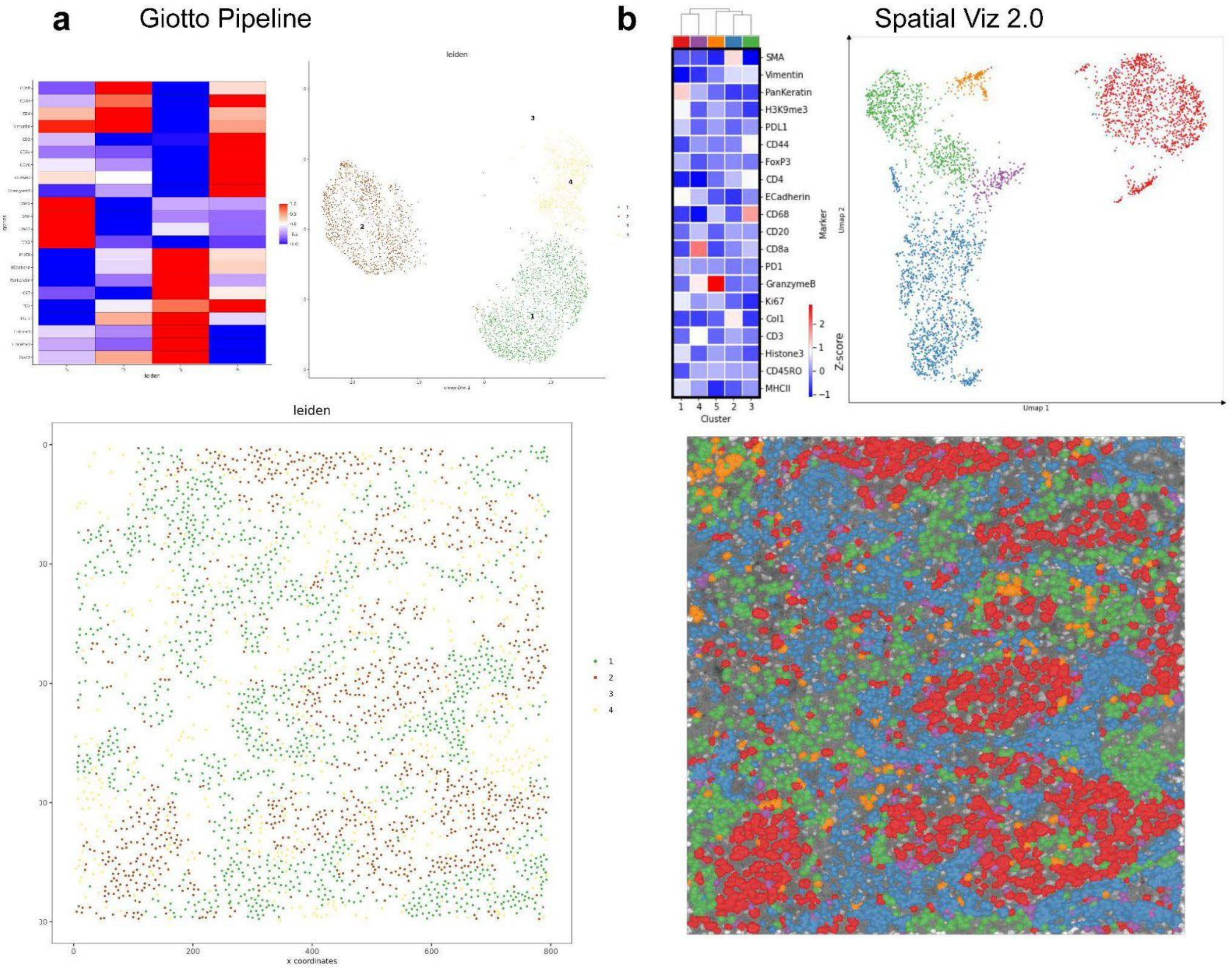
a
Giotto Pipeline
leiden
leiden
x coordinates
1
2
3
4
b
Spatial Viz 2.0
SMA
Vimentin
PanKeratin
H3K9me3
PDL1
CD44
FoxP3
CD4
ECadherin
CD68
CD20
CD8a
PD1
GranzymeB
Ki67
Col1
CD3
Histone3
CD45RO
MHCII
Marker
Z-score
Cluster
Umap 1
Umap 2

Supplementary Figure 22. Benchmarking SpatialVizScore **(a)** against Giotto **(b)** was shown. Our method results in a similar clustering result compared to Giotto. Giotto shows the centroid of the segmented cells whereas our method shows the cell mask with different morphological variations.

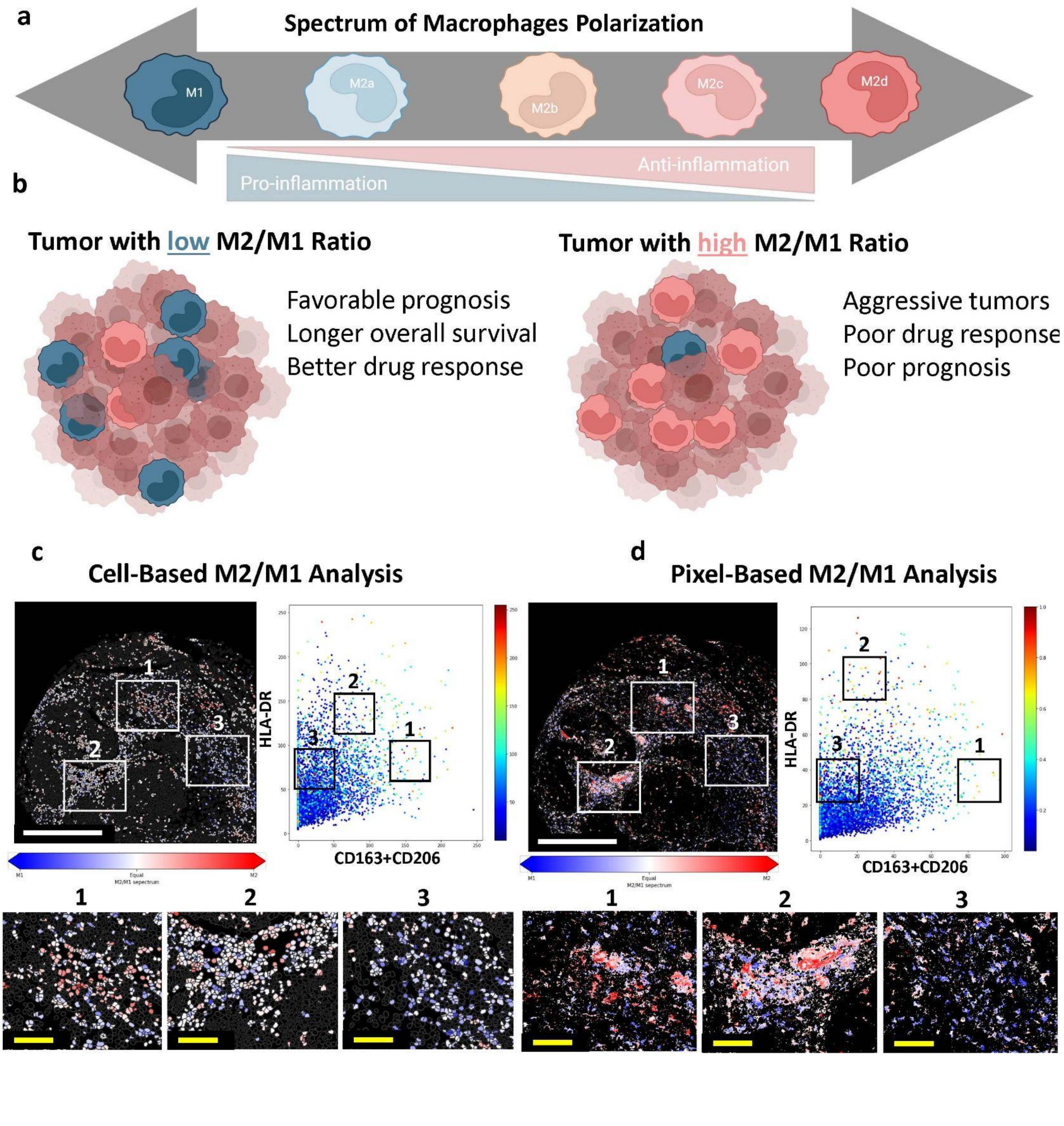
a
Spectrum of Macrophages Polarization
M1
M2a
M2b
M2c
M2d
Pro-inflammation
Anti-inflammation
b
Tumor with low M2/M1 Ratio
Favorable prognosis
Longer overall survival
Better drug response
Tumor with high M2/M1 Ratio
Aggressive tumors
Poor drug response
Poor prognosis
c
Cell-Based M2/M1 Analysis
HLA-DR
CD163+CD206
M1
Equal
M2/M1 sepectrum
M2
d
Pixel-Based M2/M1 Analysis
HLA-DR
CD163+CD206
M1
Equal
M2/M1 sepectrum
M2

Supplementary Figure 23. The spectrum of macrophage polarization was implemented within SpatialVizScore.

a Macrophages exhibit a spectrum of phenotypes that are classified as M1, M2a, M2b, M2c, and M2d. They exhibit a range of pro-inflammation to anti-inflammation functions.

b M2/M1 ratios showed a more consistent correlation with lung cancer disease progression and outcomes than the discrete count of M1 and M2 macrophages.

c-d M2/M1 ratio was quantified by two approaches including the cell-based **(c)** and the pixel-based **(d)** analysis. Created with BioRender.com, White Scale bars represent 500-µm, and yellow scale bars represent 50-µm.

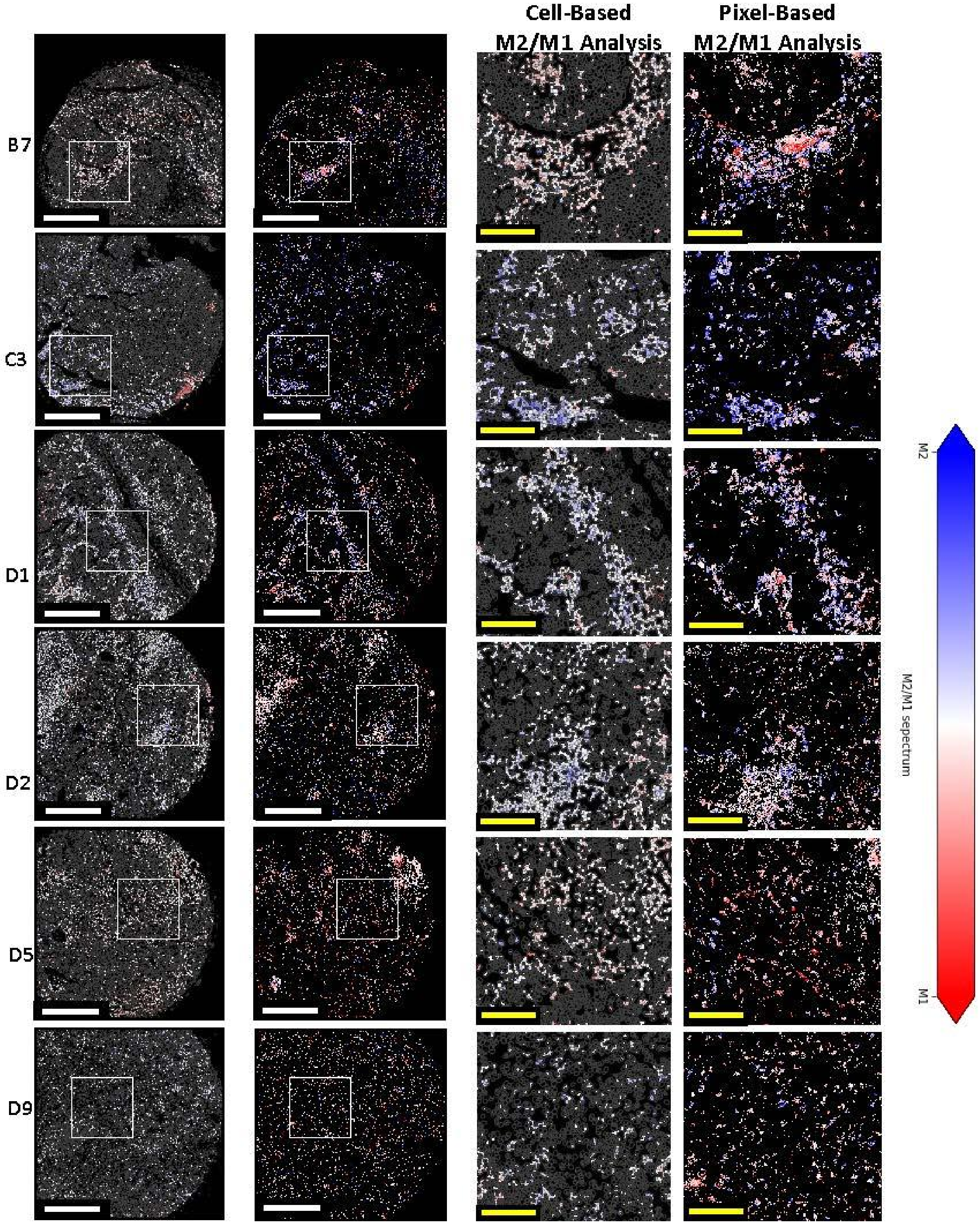

Cell-Based M2/M1 Analysis
Pixel-Based M2/M1 Analysis
B7
C3
D1
D2
D5
D9
M2
M2/M1 sepectrum
M1

Supplementary Figure 24. M2/M1 ratio analysis using both the cell-based analysis and the pixel-based analysis was presented. The first column indicated cell-based M2/M1 spectrum analysis. The second column demonstrated pixel-based M2/M1 spectrum analysis. The respective zoomed-in region was shown in the last two columns. White Scale bars represent 500-μm and yellow scale bars represent 50-μm.

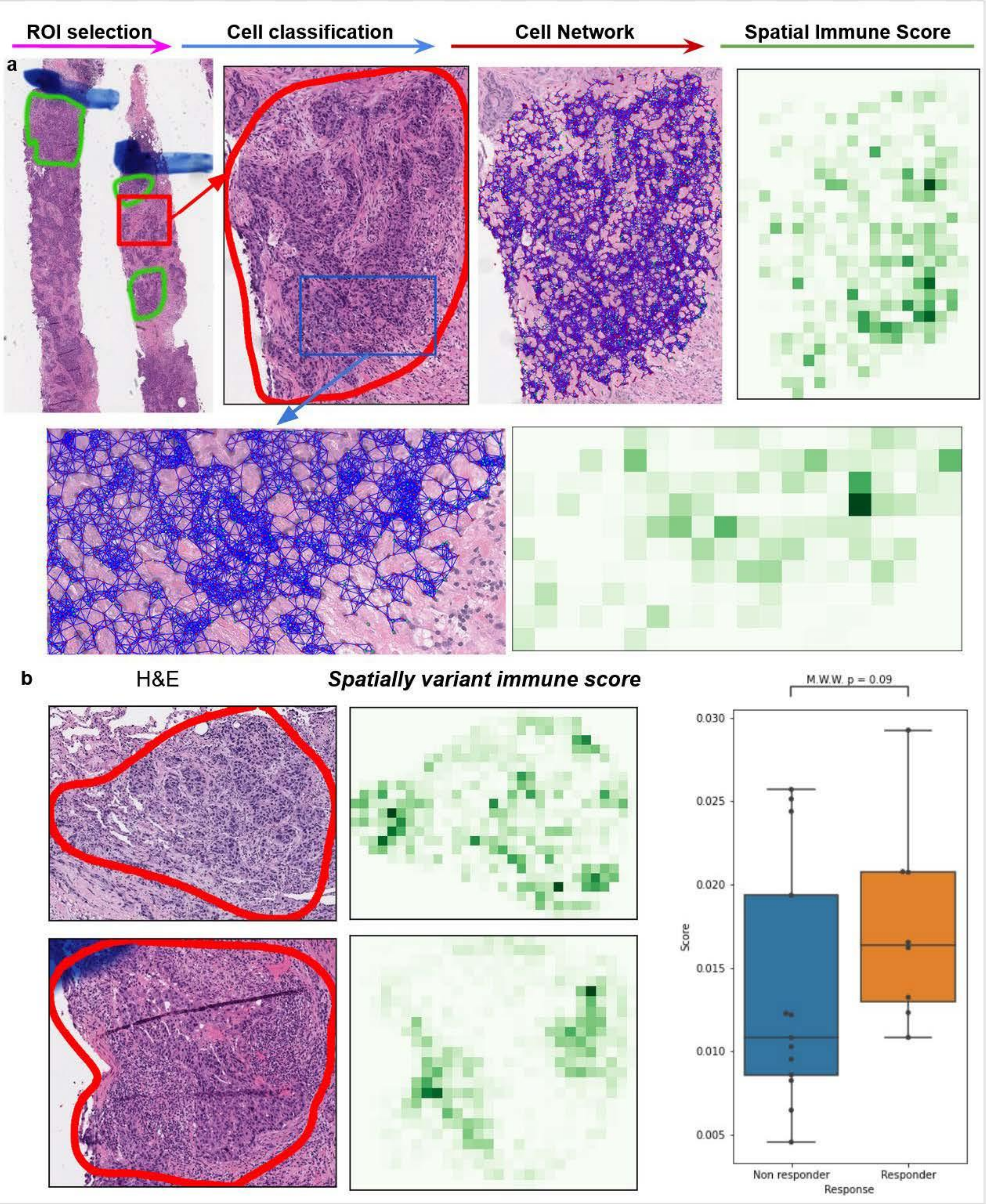

ROI selection
Cell classification
Cell Network
Spatial Immune Score
a
b
H&E
Spatially variant immune score
M.W.W. p = 0.09
0.030
0.025
0.020
0.015
0.010
0.005
Score
Non responder
Responder
Response

Supplementary Figure 25. Validation of the SpatialVizScore on breast cancer H&E images with drug response data was shown.

a The workflow of the analysis included annotating tumor regions on patients' H&E images, classifying cell phenotypes based on their morphologies, generating their network, and quantifying their neighborhood score.

b Examples of spatial variant immunoscore for non-responder (left-top) compared to responder (left-bottom). Responder patients yielded higher immunoscores than non-responder patients (right). P-value calculated using Wilcoxon Rank Sum Test. Box plot demonstrated the distribution of the data with a minimum, first quartile (Q1), median, third quartile (Q3), and maximum. Original Data was overlaid on the boxplot.

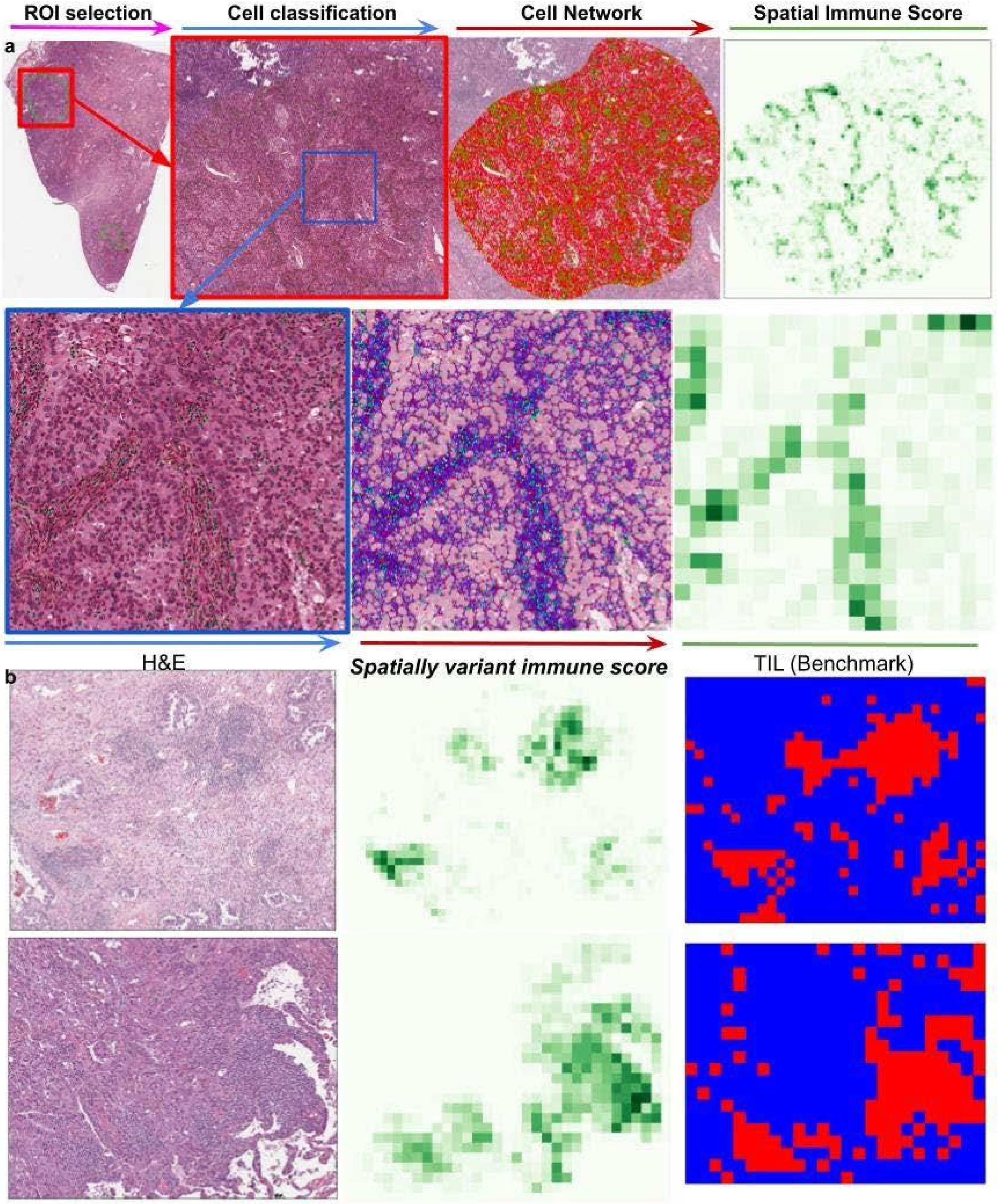
ROI selection
Cell classification
Cell Network
Spatial Immune Score
a
H&E
Spatially variant immune score
TIL (Benchmark)
b

Supplementary Figure 26. Validation of the SpatialVizScore rationale on lung cancer H&E whole slide images was demonstrated.

a The workflow of the analysis included annotating tumor regions on patients' H&E images, classifying cells phenotypes based on their morphologies, generating their network, and quantifying their neighborhood score.

b Benchmarking SpatialVizScore in generating the spatially variant immune scores that agreed well with the previously reported tumor-infiltrating lymphocytes map approach using deep learning (Saltz et al, Cell Reports 2018).

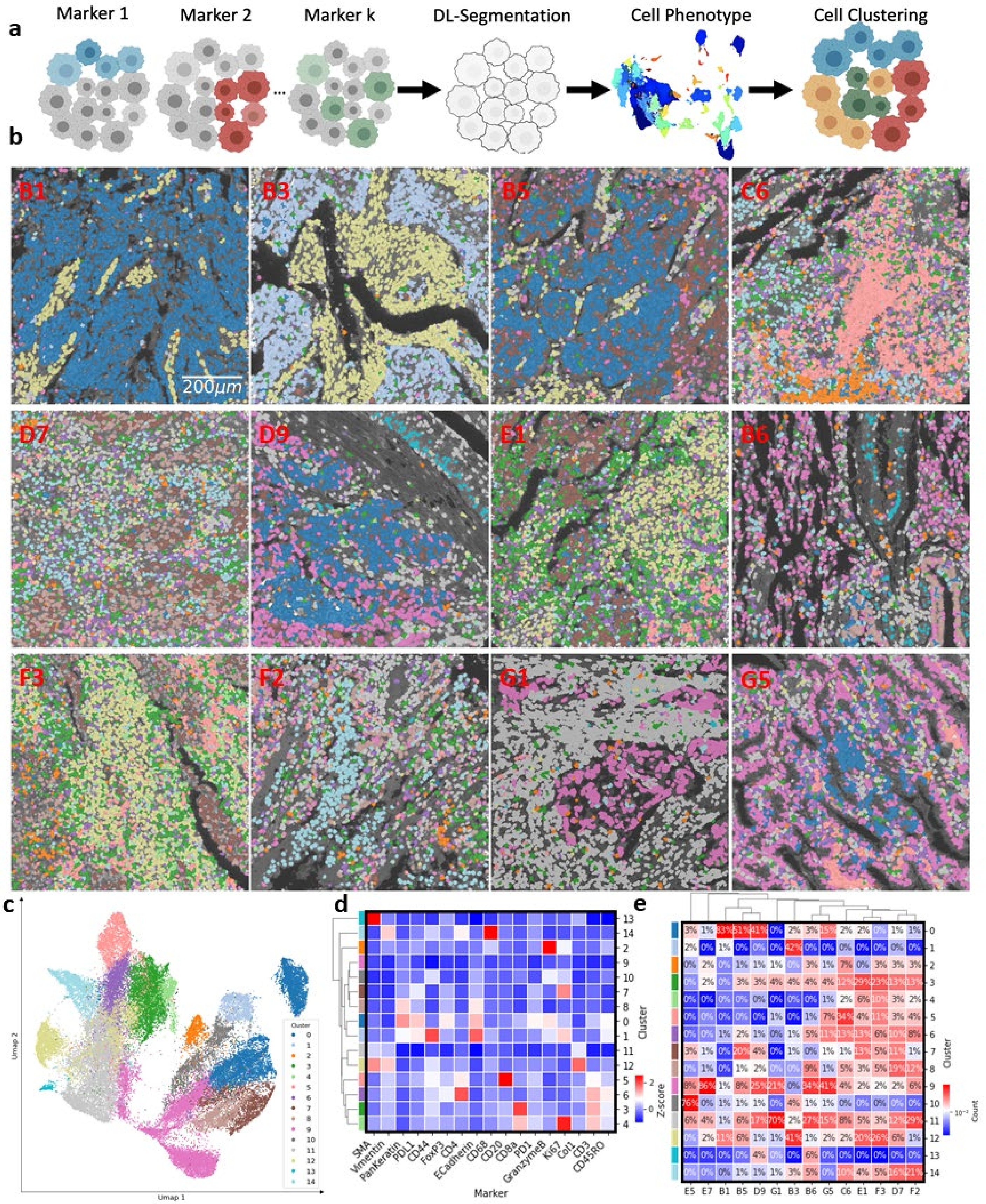
a
Marker 1
Marker 2
Marker k
DL-Segmentation
Cell Phenotype
Cell Clustering
b
B1
B3
B5
C6
200μm
D7
D9
E1
B6
F3
F2
G1
G5
c
Umap 2
Umap 1
Cluster
0
1
2
3
4
5
6
7
8
9
10
11
12
13
14
d
SMA
Vimentin
PanKeratin
PDL1
CD44
FoxP3
CD4
ECadherin
CD68
CD20
CD8a
PD1
GranzymeB
Ki67
Col1
CD3
CD45RO
Marker
Cluster
Z-score
e
E5
E7
B1
B5
D9
G1
B3
B6
G5
C6
E1
F3
D7
F2
Cluster
Count

Supplementary Figure 27. Cell-level segmentation and clustering showed the heterogeneity of markers' expressions within the patient population was presented.

a Representative schematic indicated the process of cell-level segmentation. Cell segmentation was performed using the deep-learning CellPose algorithm. The nuclei were segmented using signals from intercalators conjugated to 191Ir and 193Ir along with other markers associated with the nucleus including Histone 3, Ki67, and FoxP3. The cytosol was segmented by expanding the nuclei by 2 pixels. Number of cells n = 44,379. Created with BioRender.com

b The resulting data was clustered using all markers in the antibody panel excluding widely expressed markers including signals from intercalator 191Ir and 193Ir, histone 3, and MHC-II. The resulting clusters are visualized on the tissue samples by attributing each cell mask to the corresponding cluster color, yielding distinct separation of different anatomical regions. Scale bars represent 200-µm.

c UMAP visualized the distribution and the separation of the resulting 15 clusters from the single-cell phenotypes showing the heterogeneity in markers' expressions within the patient population.

d Correlative heatmap demonstrated the co-expression of markers across the 15 clusters that make up the data set.

e Marker abundance heatmap presented the cluster distributions for all patients' tissues.

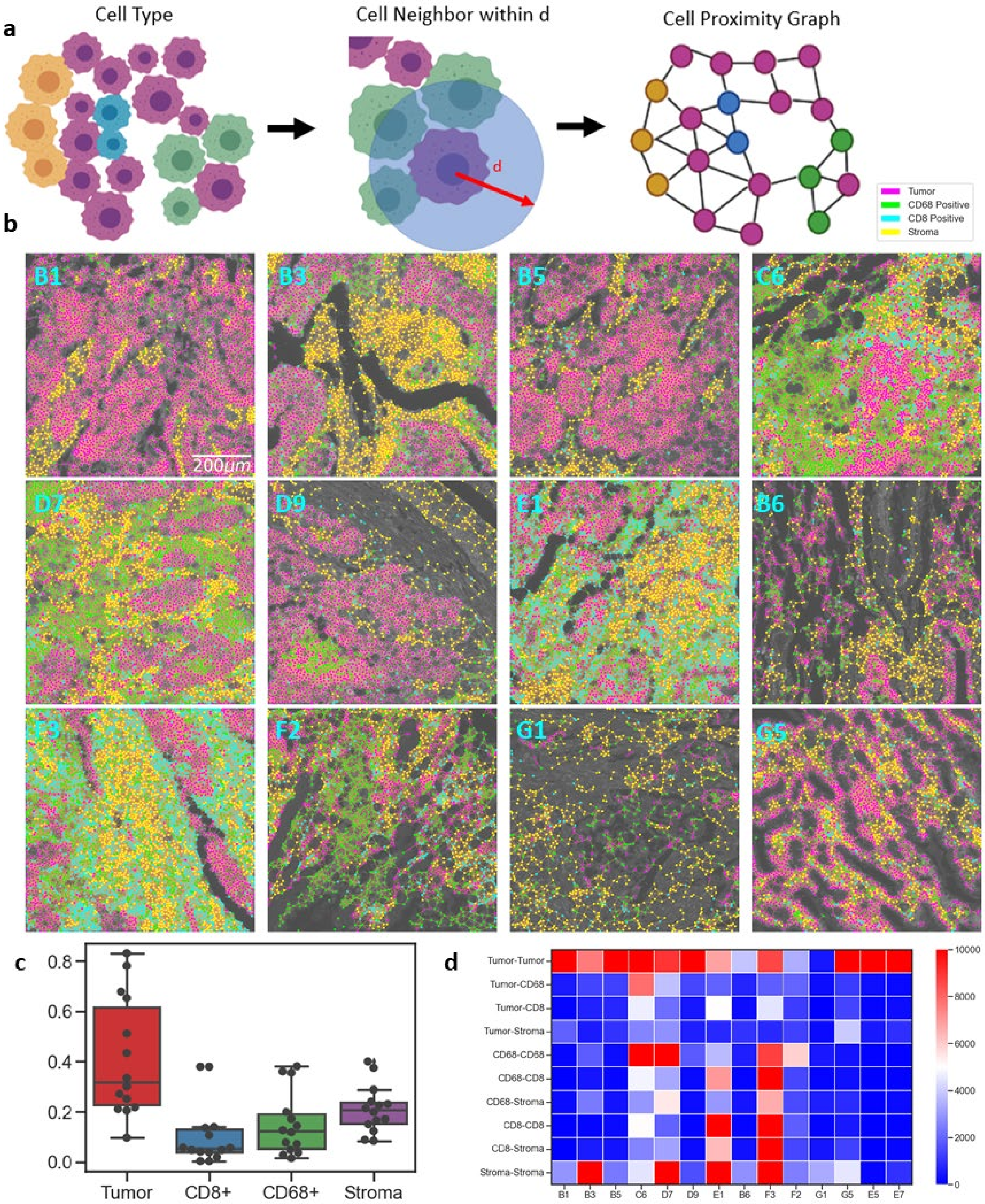

a
Cell Type
Cell Neighbor within d
d
Cell Proximity Graph
Tumor
CD68 Positive
CD8 Positive
Stroma
b
B1
B3
B5
C6
D7
D9
E1
B6
F3
F2
G1
G5
200μm
c
Tumor
CD8+
CD68+
Stroma
d
Tumor-Tumor
Tumor-CD68
Tumor-CD8
Tumor-Stroma
CD68-CD68
CD68-CD8
CD68-Stroma
CD8-CD8
CD8-Stroma
Stroma-Stroma
B1 B3 B5 C6 D7 D9 E1 B6 F3 F2 G1 G5 E5 E7
10000
8000
6000
4000
2000
0

Supplementary Figure 28. Tissue neighborhood analysis was demonstrated using single-cell data to identify the tumor/epithelial, stromal, and immune composition of patients' tissues.

a Representative schematic provided the process of neighborhood analysis. Each cell is assigned to a distinct type (tumor/epithelial, stroma, CD8+, or CD68+) based on the highest intensity values of markers. Tumor cells (T) were marked by the expression of pankeratin and e-cadherin, and stromal cells (S) were marked by the expression of collagen Type 1 and SMA. Additional cells were marked by the expression of CD8α and CD68. T-marked cells may also be major epithelial cells in paracancerous/adjacent tissues. A Cell network was generated by connecting every cell centroid to its neighboring centroids within a 30-μm distance. Created with BioRender.com

b Cell network graphs on patients' samples were shown. Magenta indicated tumor/epithelial regions, yellow demonstrated stromal regions, green corresponded to the CD68+ regions, and blue provided the CD8+ tissue regions. Scale bars represent 200-μm.

C Box plot demonstrated the tissue composition of patients' samples. Tumor/epithelial regions were the most predominant followed by stromal regions, CD68+, and lastly CD8+ regions. N = 14. Box plot demonstrated the distribution of the data with a minimum, first quartile (Q1), median, third quartile (Q3), and maximum. Original Data was overlaid on the boxplot.

d Heatmap yielded the distribution of neighborhood scores across different tissue regions.

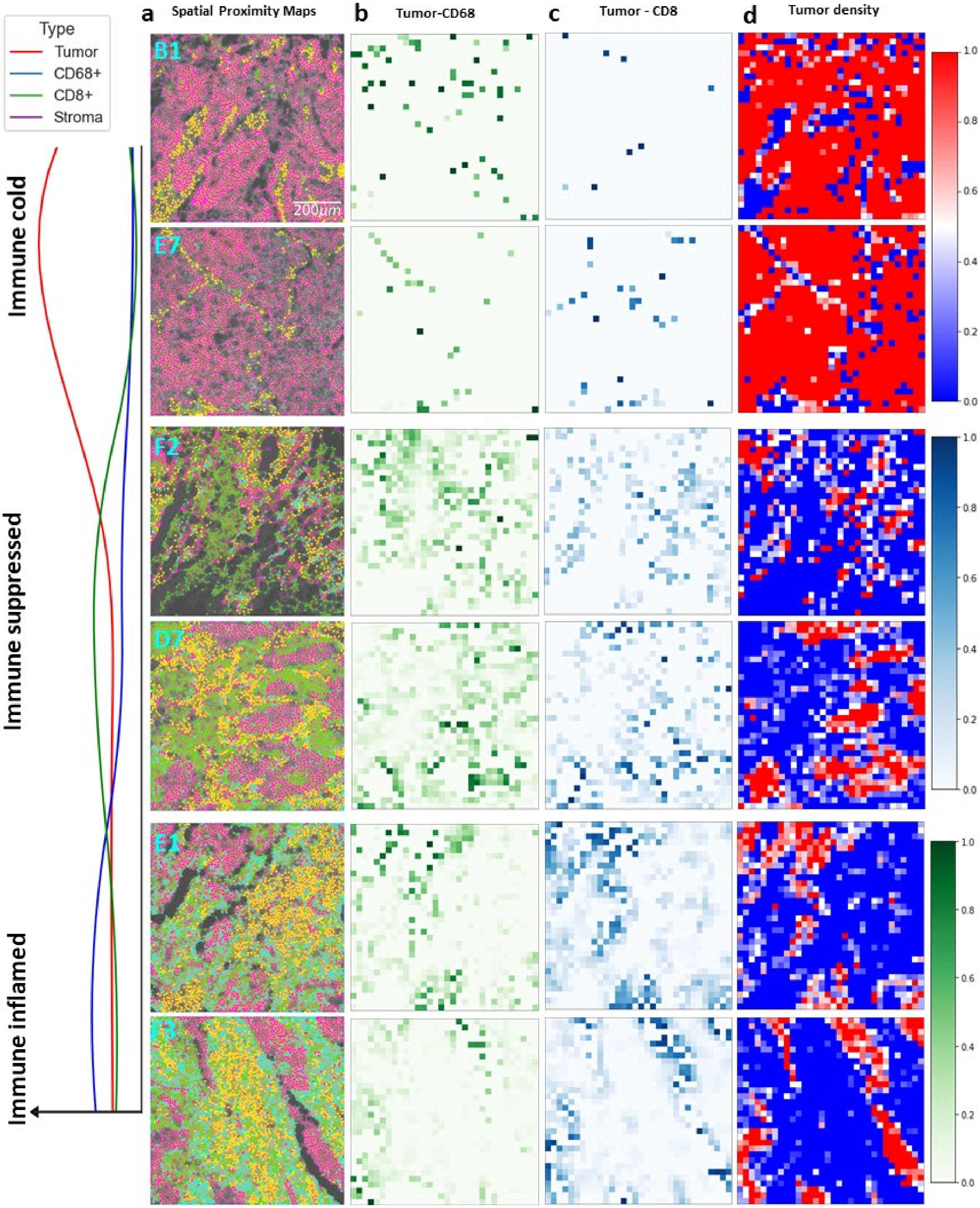

a Spatial Proximity Maps
b Tumor-CD68
c Tumor - CD8
d Tumor density
Type
Tumor
CD68+
CD8+
Stroma
Immune cold
Immune suppressed
Immune inflamed
B1
E7
F2
D7
E1
F3
200μm
1.0
0.8
0.6
0.4
0.2
0.0

Supplementary Figure 29. Spatial interaction maps and immunoscoring of the tumor microenvironment in lung cancer were presented using single-cell segmentation masks to reveal the immune continuum of patients' cancerous and paracancerous tissues.

a Cell network and spatial proximity maps on lung cancer samples demonstrated the interaction of tumor/T (in magenta), stroma/S (in yellow), tumor-CD68 (in green), and tumor-CD8 (in blue). T-marked cells may also be major epithelial cells in paracancerous/adjacent tissues. Scale bars represent 200-µm.

b Spatial interaction map provided the distribution of tumor/epithelial and CD68+ immune cells. It follows a continuum where it's highest for immune inflamed and immune suppressed tumors but damps down for immune cold tumors.

c Spatial interaction map yielded the distribution of tumor/epithelial and CD8+ immune cells. It follows a continuum across immune inflamed, immune suppressed, and immune cold tumors.

d Spatial density map demonstrated the distribution of tumor/epithelial density across the patients' samples.

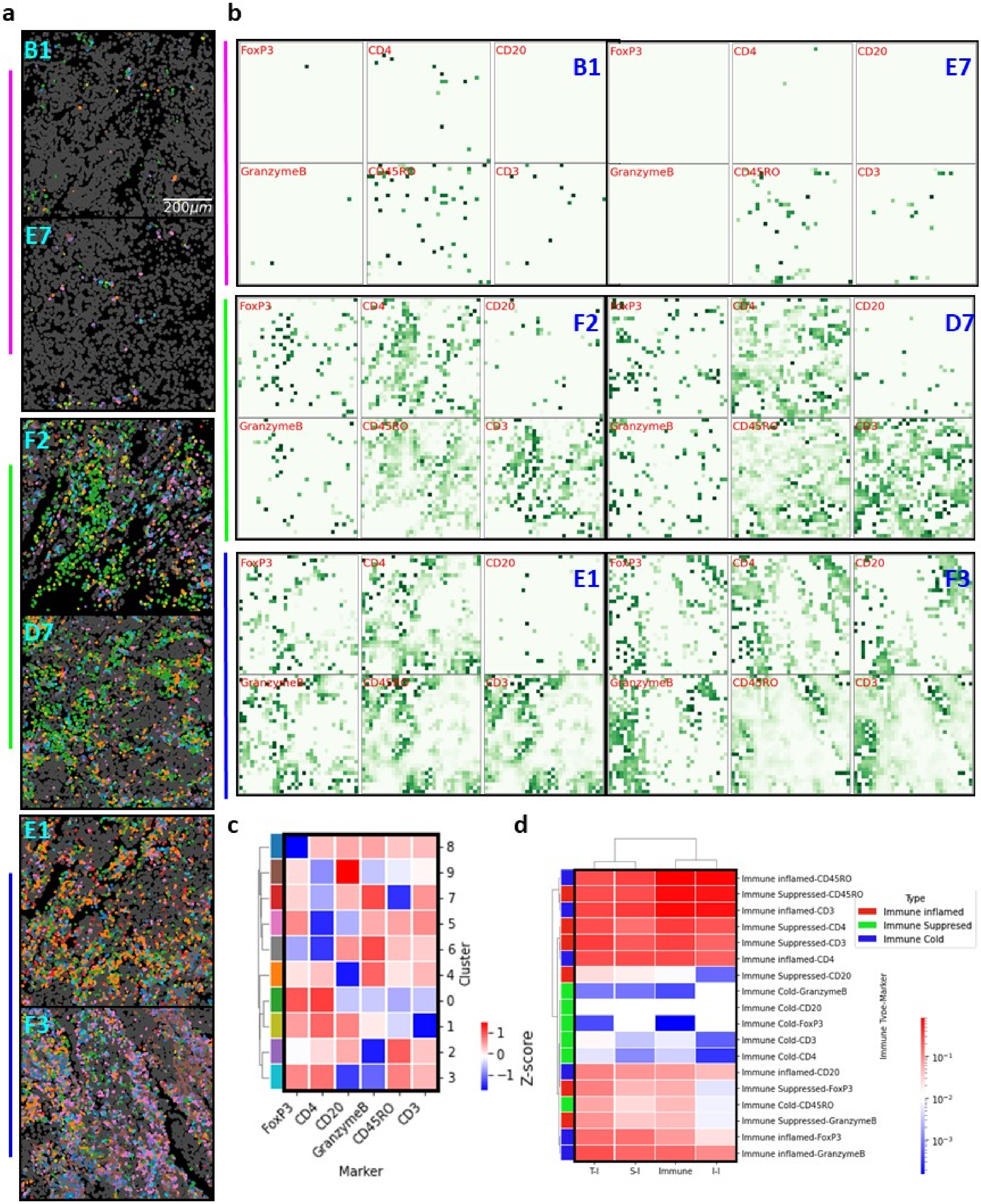

a
b
c
d
B1
E7
F2
D7
E1
F3
200μm
FoxP3
CD4
CD20
GranzymeB
CD45RO
CD3
Cluster
Marker
Z-score
Type
Immune inflamed
Immune Suppresed
Immune Cold
Immune Type-Marker
Immune inflamed-CD45RO
Immune Suppressed-CD45RO
Immune inflamed-CD3
Immune Suppressed-CD4
Immune Suppressed-CD3
Immune inflamed-CD4
Immune Suppressed-CD20
Immune Cold-GranzymeB
Immune Cold-CD20
Immune Cold-FoxP3
Immune Cold-CD3
Immune Cold-CD4
Immune inflamed-CD20
Immune Suppressed-FoxP3
Immune Cold-CD45RO
Immune Suppressed-GranzymeB
Immune inflamed-FoxP3
Immune inflamed-GranzymeB
T-I
S-I
Immune
I-I

Supplementary Figure 30. Spatial immune phenotyping, interaction maps, and immunoscoring of the tumor microenvironment in lung cancer were presented using single-cell segmentation masks to the unique distribution of immune infiltration patterns.

a Cell mask on lung cancer samples represented the immune phenotypes in cancerous and paracancerous tissue samples, highlighting the cluster distribution on the patients' tissues. Scale bars represent 200-μm.

b Spatial interaction map provided the distribution of tumor/epithelial and immune cell markers across the three different immune states.

c Correlative heatmap demonstrated the co-expression of markers across the 10 clusters from the multiplexed dataset.

d Heatmap yielded the distribution of scores from the marker neighborhoods across different tissue regions.

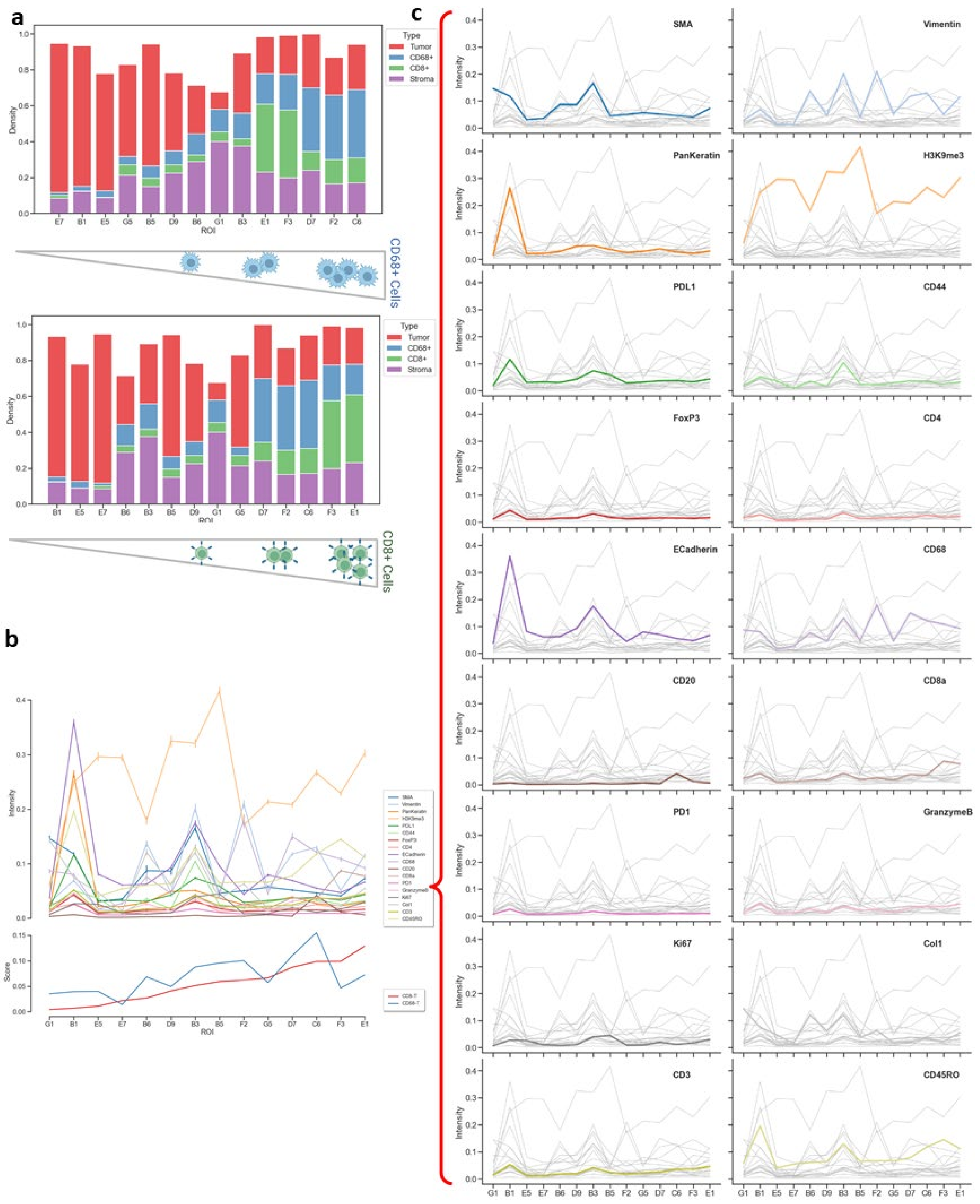
a
b
c
Type
Tumor
CD68+
CD8+
Stroma
Density
ROI
E7 B1 E5 G5 B5 D9 B6 G1 B3 E1 F3 D7 F2 C6
B1 E5 E7 B6 B3 B5 D9 G1 G5 D7 F2 C6 F3 E1
CD68+ Cells
CD8+ Cells
Intensity
Score
G1 B1 E5 E7 B6 D9 B3 B5 F2 G5 D7 C6 F3 E1
CD8-T
CD68-T
SMA
Vimentin
PanKeratin
H3K9me3
PDL1
CD44
FoxP3
CD4
ECadherin
CD68
CD20
CD8a
PD1
GranzymeB
Ki67
Col1
CD3
CD45RO

Supplementary Figure 31. The immune continuum for lung cancer patients, containing cancerous and paracancerous tissues, ranges can be classified into immune inflamed, immune cold, and immune desert.

a Bar graphs with stacked columns demonstrated the composition of patients' tissues from tumor/epithelial in red, stroma in magenta, CD68+ in blue, and CD8+ in green. The top bar graph corresponded to the composition of patients' tissues from the lowest to highest CD68+ scores whereas the bottom bar graph displayed the composition of patients' tissues from the lowest to highest CD8+ scores.

b The distribution of immune markers across patients' samples (top) after arranging them from the lowest CD8+ to the highest (bottom).

c Individual marker distribution plots across patients samples were arranged from the lowest immune CD8+ scores (immune cold tumors) to the highest immune CD8+ cores (immune inflamed tumors). The markers included SMA, Vimentin, Pankeratin, H3K9me3, PD-L1, CD44, FoxP3, CD4, E-cadherin, CD68, CD20, CD8α, PD1, Granzyme B, Ki67, collagen 1, CD3, and CD45RO.

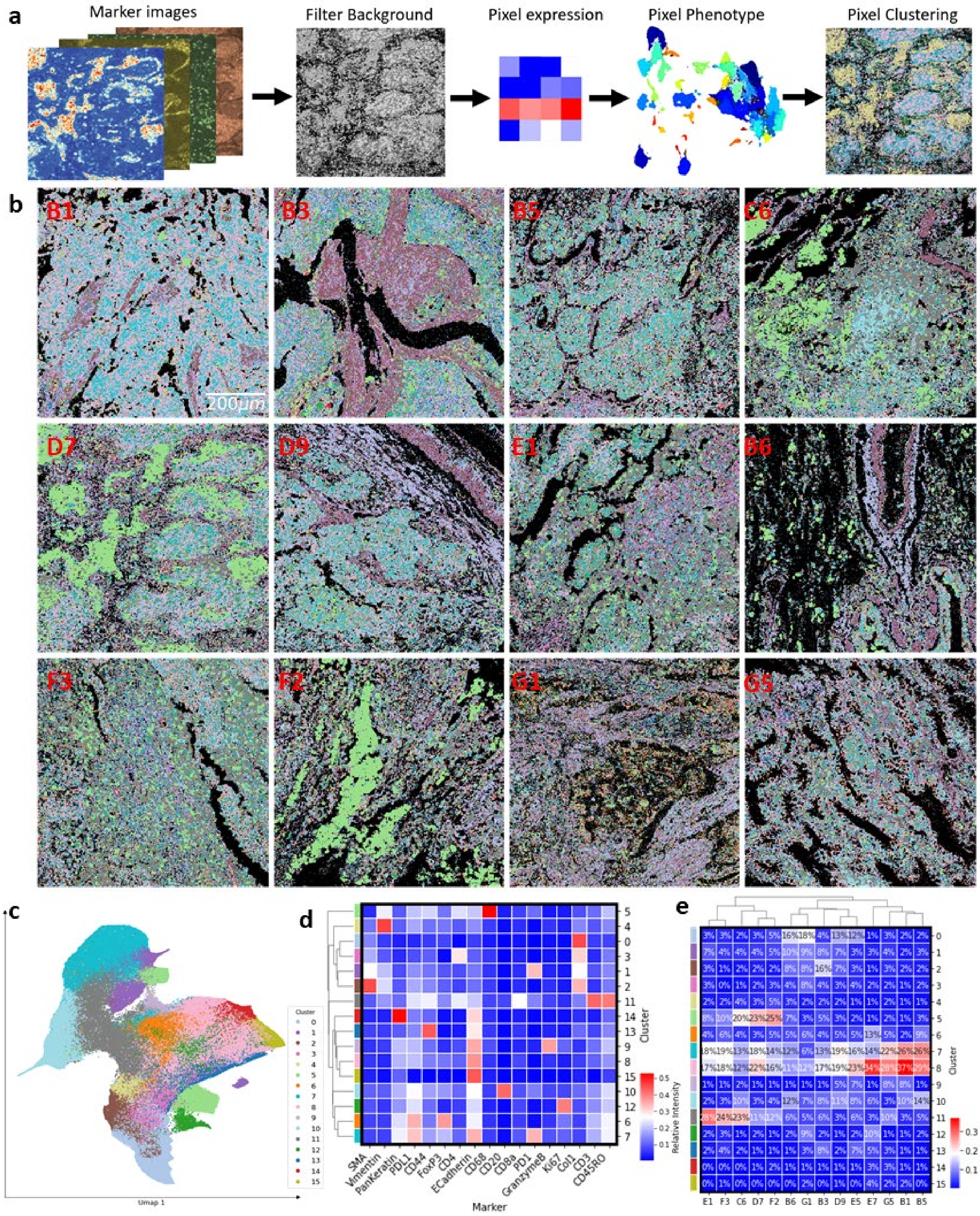
a
Marker images
Filter Background
Pixel expression
Pixel Phenotype
Pixel Clustering
b
B1
200μm
B3
B5
C6
D7
D9
E1
B6
F3
F2
G1
G5
c
Cluster
0
1
2
3
4
5
6
7
8
9
10
11
12
13
14
15
Umap 1
d
SMA
Vimentin
PanKeratin
PDL1
CD44
FoxP3
CD4
ECadherin
CD68
CD20
CD8a
PD1
GranzymeB
Ki67
Col1
CD3
CD45RO
Marker
Cluster
Relative Intensity
e
E1
F3
C6
D7
F2
B6
G1
B3
D9
E5
E7
G5
B1
B5
Cluster
Count

Supplementary Figure 32. Pixel-level classification and clustering reveal the distinct distributions within patients' samples.

a Representative schematic demonstrates the process of pixel classification. The intensity information for all markers at all pixel locations (pixel size = 1-µm) was extracted and normalized. The background signal was filtered by eliminating pixel locations with an intensity value lower than 0.3. The resulting marker intensity data considered as non-background was clustered. Number of pixels n = 1,589,784.

b The resulting pixel intensity data was clustered using all markers in the antibody panel excluding widely expressed markers including signals from intercalator 191Ir and 193Ir, histone 3, and MHC-II. The resulting clusters are visualized on the tissue samples by attributing each pixel to the corresponding cluster color, indicating distinct separation of different anatomical regions. Scale bars represent 200-µm.

c UMAP visualized the distribution and the separation of the resulting 16 clusters from the pixel-level phenotypes.

d Correlative heatmap demonstrated the co-expression of markers across the 16 clusters that make up the data set.

e Marker abundance heatmap provided the cluster compositions for all patients' tissues.

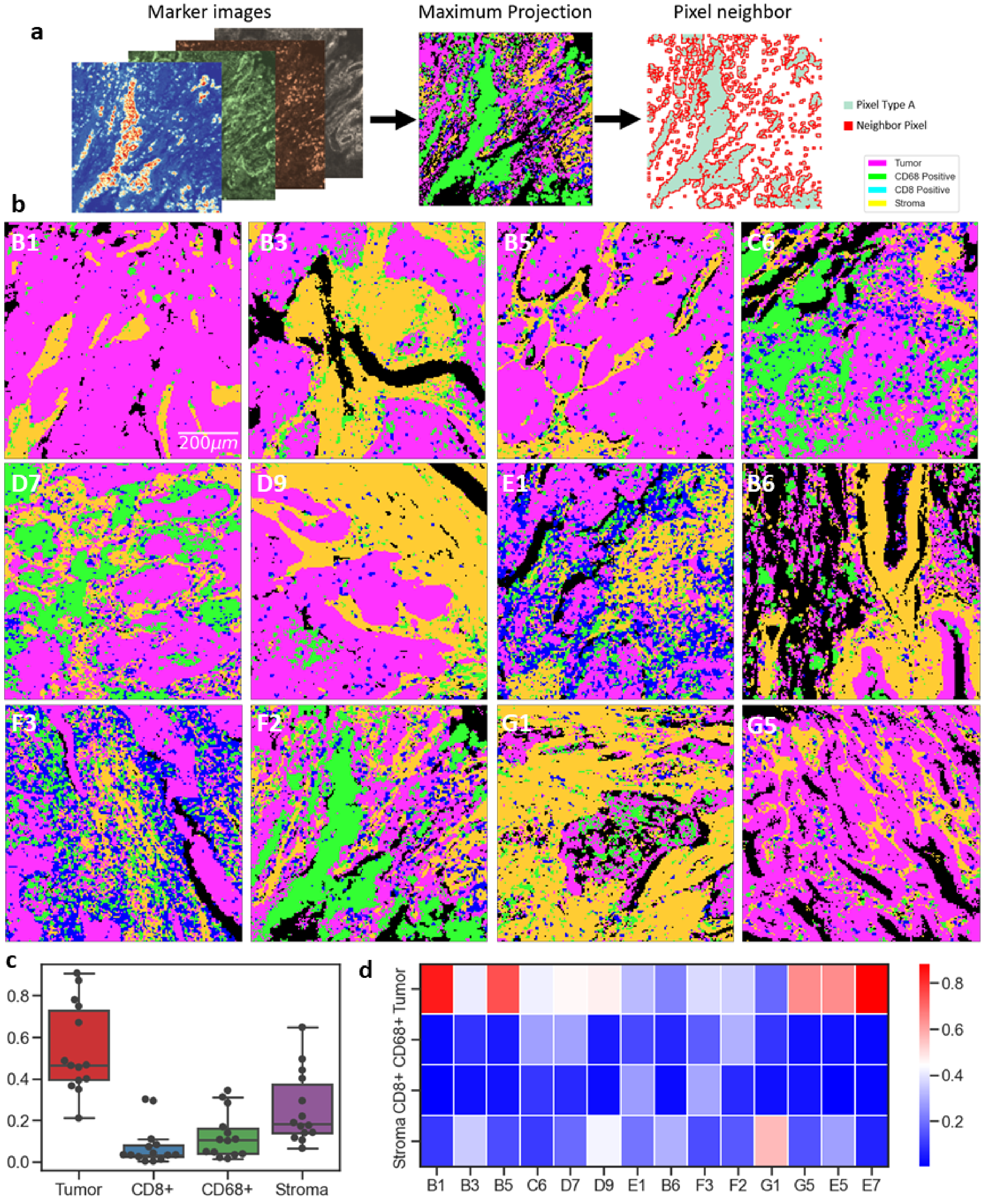
a
Marker images
Maximum Projection
Pixel neighbor
Pixel Type A
Neighbor Pixel
Tumor
CD68 Positive
CD8 Positive
Stroma
b
B1
200μm
B3
B5
C6
D7
D9
E1
B6
F3
F2
G1
G5
c
0.8
0.6
0.4
0.2
0.0
Tumor
CD8+
CD68+
Stroma
d
Stroma CD8+ CD68+ Tumor
B1 B3 B5 C6 D7 D9 E1 B6 F3 F2 G1 G5 E5 E7
0.8
0.6
0.4
0.2

Supplementary Figure 33. Spatial interaction maps and immunoscoring of the tumor microenvironment in lung cancer using pixel-level classification outperform in segmenting stromal regions.

a Pre-identified marker images were used to mark distinct tissue regions (Tumor/epithelial: pankeratin and e-cadherin, Stroma: SMA and collagen Type 1, CD8ɑ, and CD68) resulting in 4 final images. The maximum projection image is generated by dividing each ROI image into patches with pixel size = 5-µm where each patch is assigned a tissue region based on the maximum intensity value of the pre-identified markers list. Pixel neighborhood analysis was then performed within the unique tissue regions.

b Maximum projection images of ROIs were shown using magenta for tumor/epithelial regions, yellow for stromal regions, green for CD68+ regions, and blue for CD8+ regions. Scale bars represent 200-µm.

c Box plot indicated the tissue composition of patients' samples. Tumor/epithelial regions are the most predominant followed by stromal regions, CD68+, and lastly CD8+ regions. N = 14. Box plot demonstrated the distribution of the data with a minimum, first quartile (Q1), median, third quartile (Q3), and maximum. Original Data was overlaid on the boxplot.

d Heatmap demonstrated the density across different tissue regions.

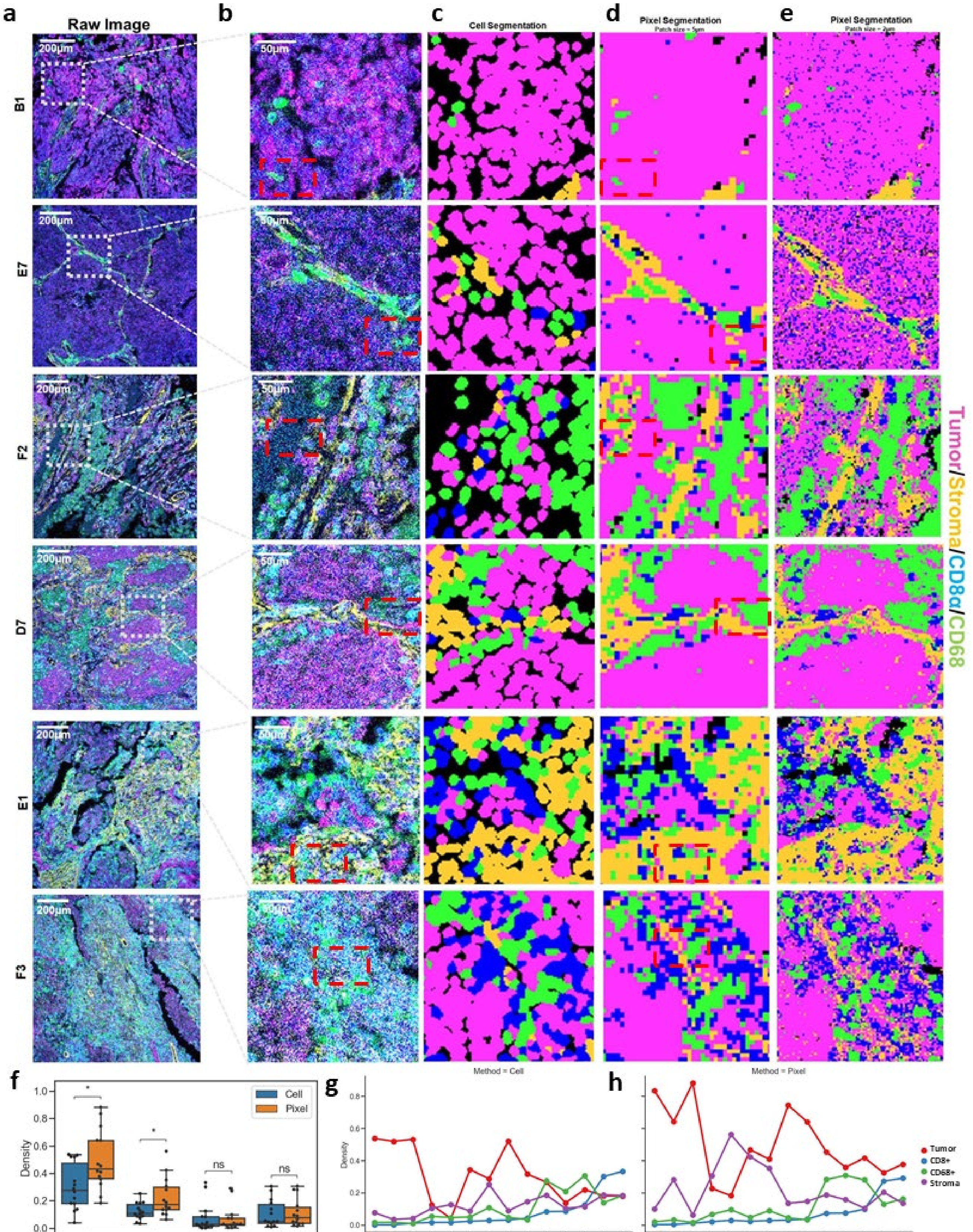
a
Raw Image
b
c
Cell Segmentation
d
Pixel Segmentation
Patch size = 5μm
e
Pixel Segmentation
Patch size = 2μm
B1
E7
F2
D7
E1
F3
200μm
50μm
Tumor/Stroma/CD8α/CD68
f
Cell
Pixel
Density
Tumor
Stroma
CD8+
CD68+
ns
g
Method = Cell
h
Method = Pixel
ROI
Tumor
CD8+
CD68+
Stroma

Supplementary Figure 34. Pixel-level immune microenvironment classification outperforms the cell-level segmentation in identifying stromal and dense tumor regions.

a Tissue regions in an 800-μmx800-μm section can be visualized by assigning false colors to isotope signals and their conjugated antibodies and delimiter showing the region of comparison in each ROI. Scale bars represent 200-μm.

b Zoomed-in views of tissue regions in each ROI were shown. Scale bars represent 50-μm.

c Single cell level reconstruction of tissue was presented. The view was created with the cell mask assigned to the corresponding cell type color.

d Pixel level reconstruction of tissue was demonstrated. The view was created with pixel patches region assigned to corresponding marker type color with patches size of 5.

e Pixel-level reconstruction of tissue was presented. The view was created with pixel patches region assigned to corresponding marker type color with patches size of 2.

f Comparison of area density for tumor, stroma, CD8+, and CD68+ between single cell and pixel-level tissue reconstruction method. N = 14. Asterisk indicates the statistical significance for pairwise comparison. P-value calculated using Wilcoxon Rank Sum Test (ns: $0.05 < p$, *: $p <= 0.01$). Box plot demonstrated the distribution of the data with a minimum, first quartile (Q1), median, third quartile (Q3), and maximum. Original Data was overlaid on the boxplot.

g Line plot demonstrated the variation of area density across ROI for single cell level reconstruction of tissue.

h Line plot provided the variation of area density across ROI for the pixel-level reconstruction of tissue.

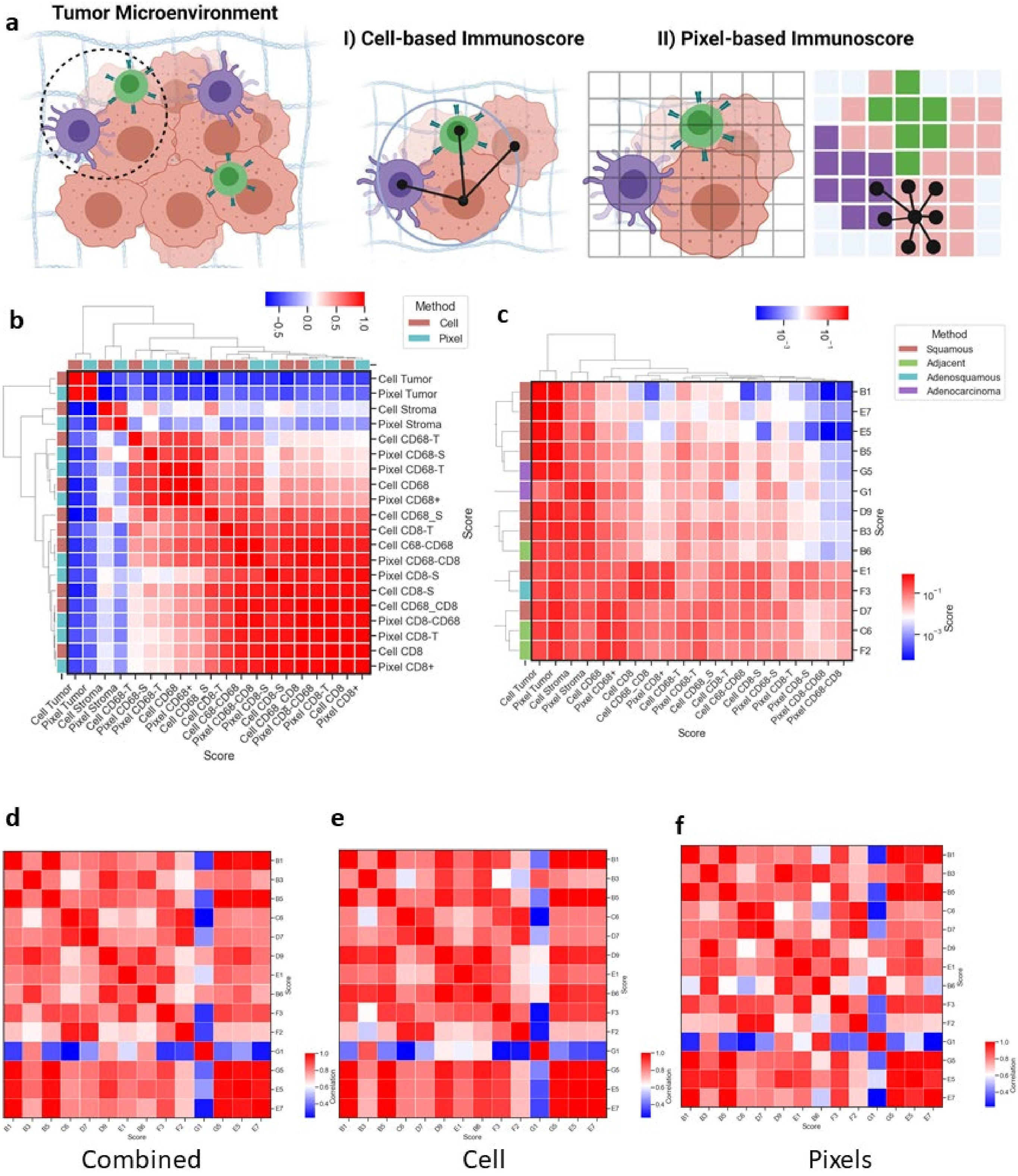
a
Tumor Microenvironment
I) Cell-based Immunoscore
II) Pixel-based Immunoscore
b
Method
Cell
Pixel
Cell Tumor
Pixel Tumor
Cell Stroma
Pixel Stroma
Cell CD68-T
Pixel CD68-S
Pixel CD68-T
Cell CD68
Pixel CD68+
Cell CD68_S
Cell CD8-T
Cell C68-CD68
Pixel CD68-CD8
Pixel CD8-S
Cell CD8-S
Cell CD68_CD8
Pixel CD8-CD68
Pixel CD8-T
Cell CD8
Pixel CD8+
Score
c
Method
Squamous
Adjacent
Adenosquamous
Adenocarcinoma
B1
E7
E5
B5
G5
G1
D9
B3
B6
E1
F3
D7
C6
F2
Score
d
e
f
Combined
Cell
Pixels

Supplementary Figure 35. Cell-level segmentation and pixel-level classification are in agreement in immunoscoring lung cancer patients' samples.

a Cell-level segmentation and pixel-level classification analysis were used to quantify spatially variant immunoscores. Created with BioRender.com

b Heatmaps demonstrated the cell-level and the pixel-level immunoscores for all cancer subtypes that were included within the patient population.

c Representative schematic provided the difference between cell-based and pixel-based immunoscoring. The cell-based immunoscore relies on cell segmentation masks to identify individual cells. The cell network is generated by identifying the distance between cell centroids within a diameter of 30 µm distance. The pixel-level immunoscore relies on the pixel-based classification where the 800 µm x 800 µm patients' tissues are divided into 160x160 square patches with patch size = 5 µm. Each patch is assigned a phenotype based on the highest intensity of marker expression. Similar to the cell network, the pixel network is generated to assess the neighborhood of the anatomical tissue region.

d-f Correlation heatmaps between all scores for all ROIs were generated by d cell-level segmentation and pixel-level classification, e cell-level segmentation only, and f pixel-level classification only.

Supplementary Tables

Marker	Clone	Metal Tag	Dilution
CD20	H1	161Dy	1:400
CD3	Polyclonal	170Er	1:100
CD4	ERP6855	156Gd	1:200
CD45RO	UCHL1	173Yb	1:50
CD68	KP1	159Tb	1:50
CD8a	C8/144B	162Dy	1:100
FoxP3	236A/E7	155Gd	1:30
Pan-Keratin	C11	148Nd	1:100
Granzyme B	EPR20129-17	167Er	1:100
Ki-67	B56	168Er	1:50
PD-1	ERP4877(2)	165Ho	1:50
PD-L1	SP142	150Nd	1:50
SMA	1A4	141Pr	1:200
Collagen Ty1	Polyclonal	169Tm	1:300
E-cadherin	24E10	158Gd	1:50
Histone 3	D1H2	171Yb	1:50
Vimentin	D21H3	143Nd	1:100
CD44	IM7	153Eu	1:100
MHC-II	6C6	175Lu	1:200
H3K9me3	EPR16601	149Sm	1:400
HLA-DR	LN3	174Yb	1:200
CD11c	Polyclonal	154Sm	1:100

CD163	EDHu-1	147Sm	1:100
CD206	5C11	152Sm	1:200
CD95	EPR5700	160Gd	1:250
CD103	EPR4166(2)	151Eu	1:400
TCF1	C63D9	144Nd	1:35
Intercalator	—	191Ir/193Ir	1:400

Supplementary Table 1. Antibody panel used for imaging mass cytometry.

**Patient Tissue**	**Age**	**Sex**	**Organ/ Anatomic Site**	**Pathology Diagnosis**	**TNM**	**Grade**	**Stage**	**Type**
**TMA: LC814a (Cohort 1)**								
B7	42	M	Lung	Squamous cell carcinoma	T3N1M0	3	IIIA	Malignant
C3	63	F	Lung	Squamous cell carcinoma	T3N1M0	3	IIIA	Malignant
D1	65	M	Lung	Adenocarcinoma	T2N1M0	3	IIIA	Malignant
D2	59	M	Lung	Adenocarcinoma	T3N1M0	3	IIIA	Malignant
D5	60	M	Lung	Adenocarcinoma	T2N1M0	3	IIA	Malignant
D9	52	F	Lung	Adenocarcinoma	T2N1M0	3	IIA	Malignant
F7	42	M	Lymph Node	Metastatic Squamous cell carcinoma of B7	–	–	–	Malignant
G3	63	F	Lymph Node	Metastatic Squamous cell carcinoma of C3	–	–	–	Malignant
H1	65	M	Lymph Node	Metastatic Adenocarcinoma of D1	–	–	–	Malignant
H2	59	M	Lymph Node	Metastatic Adenocarcinoma of D2	–	–	–	Malignant
H5	60	M	Lymph Node	Metastatic Adenocarcinoma of D5	–	–	–	Malignant
H9	52	F	Lymph Node	Metastatic Adenocarcinoma of D9	–	–	–	Malignant
**TMA: LC1002a (Cohort 2)**								

B1	63	F	Lung	Squamous cell carcinoma	T1N0M0	2	I	Malignant
B3	55	M	Lung	Squamous cell carcinoma	T2N0M0	2	I	Malignant
B5	39	F	Lung	Squamous cell carcinoma	T2N1M0	2	II	Malignant
C6	40	M	Lung	Cancer adjacent lung tissue	-	-	-	-
D7	66	M	Lung	Squamous cell carcinoma	T2N2M0	3	IIIA	Malignant
D9	65	M	Lung	Squamous cell carcinoma	T2N0M0	3	I	Malignant
E1	56	M	Lung	Squamous cell carcinoma	T2N1M0	2	II	Malignant
B6	39	F	Lung	Cancer adjacent lung tissue	-	-	-	-
F3	54	M	Lung	Adenosquamous carcinoma	T2N1M0	3*	II	Malignant
F2	58	M	Lung	Cancer adjacent lung tissue	-	-	-	-
G1	73	M	Lung	Adenocarcinoma	T2N0M0	2	I	Malignant
G5	54	F	Lung	Mucinous adenocarcinoma	T3N0M0	1	IIIA	Malignant
E5	52	M	Lung	Squamous cell carcinoma	T4N1M0	2*	IIIB	Malignant
E7	65	M	Lung	Squamous cell carcinoma	T2N1M0	3	II	Malignant

Supplementary Table 2. Patient specifications for cancer type, stage, and grade.

Sample	Tumor-Tumor	Tumor-M1	Tumor-M2	Tumor-CD8+	Tumor-Stroma	M1-M1	M1-M2	M1-CD8+	M2-M2	M2-CD8+	CD8+-Stroma	CD8+-CD8+	Stroma-Stroma
B7	283720	10898	4977	44405	10951	10192	2952	12117	3048	6876	11726	35756	21186
C3	397902	25135	16261	81736	28903	16296	5734	14485	21286	27163	32882	96316	82006
D1	470050	28797	14711	42048	18751	31136	8306	34895	7066	12230	27184	91010	37874

D2	164708	17011	4965	44354	21602	14204	2072	18882	1822	5987	27220	68796	102156
D5	603728	46266	7343	61557	42108	13456	681	6126	1430	1276	5785	11650	14220
D9	216636	37595	10643	72512	82918	14862	2658	18767	1802	5427	35400	41674	119520
F7	124674	3821	452	17619	5504	5288	893	7473	468	1612	9758	21020	23276
G3	538956	43917	8921	78040	10940	34028	4328	20792	6538	13019	34509	78340	85478
H1	864988	49826	14092	36230	37235	53738	2169	14516	1694	1531	5102	7884	16476
H2	90874	38341	7646	21055	9403	57150	9666	17039	10136	6908	14657	17892	47862
H5	600544	13510	3018	73305	18030	9562	424	3672	346	838	5251	35544	18108
H9	40202	22480	1315	20731	18218	107824	5905	28223	974	2224	21618	20908	64622

	Cell count density		Cell neighbor interaction density			
**Patient Sample**	**Tumor**	**Stroma**	**IP_T**	**IP_S**	**IN_T**	**IN_S**
**B1**	**0.7808**	**0.1222**	**0.0068**	**0.0008**	**0.0393**	**0.0132**
**B3**	0.3335	0.3743	0.0512	0.0331	0.0877	0.1076
**B5**	**0.6776**	**0.1491**	**0.059**	**0.0275**	**0.0957**	**0.03**
**C6**	0.251	0.1695	0.0987	0.0695	0.1551	0.081
**D7**	0.2997	0.2393	0.0872	0.099	0.11	0.1439
**D9**	0.4331	0.2247	0.0406	0.0256	0.0494	0.0267
**E1**	0.2044	0.2297	0.1289	0.1464	0.0723	0.0972
**B6**	0.2703	0.2873	0.0268	0.0272	0.0683	0.0462
**F3**	0.217	0.1978	0.099	0.1726	0.0462	0.1306
**F2**	0.2098	0.1641	0.0621	0.0653	0.1004	0.0732
**G1**	0.0955	0.3996	0.004	0.0587	0.035	0.0759
**G5**	**0.5114**	**0.213**	**0.0663**	**0.0619**	**0.0571**	**0.0445**
**E5**	**0.6521**	**0.0871**	**0.0108**	**0.0004**	**0.0399**	**0.0095**
**E7**	**0.8312**	**0.0833**	**0.0216**	**0.0117**	**0.0141**	**0.0135**

Supplementary Table 3. Spatially variant Immunocore using the cell-based segmentation

Marker	Immune Cell Phenotype	Justification for adding to the immunoscore	Ref
CD33+<br>CD45+<br>CD32+<br>CD117+<br>CD203+<br>MTF+	Mast cells	Mast cells showed an inconsistent prognostic effect on different solid tumors suggesting that they can act both as pro-tumor and antitumor cells. It could be very valuable to investigate mast cells in lung cancers.	PMID: 29651440<br>PMID: 28446910
CD3+<br>CD4+<br>CTLA-4+<br>LAG-3+<br>OX40+<br>CD62L+<br>FoxP3+<br>STAT5+	Regulatory T cells	Treg cells are generally associated with poor clinical outcomes and reduced overall survival. However, Treg cells in different regions of the tumor hold different prognostic values. For example, high intratumoral Treg cells are associated with much shorter survival when compared with peritumoral Treg cells. They also hold a precious clinical value to determine patients' eligibility to receive immune checkpoint inhibitors. Treg could be a valuable addition to the immunoscores to further increase their clinical impact.	PMID: 26462617
CD80+<br>CD86+<br>CCR5+<br>CD11b+<br>CD11c+<br>CD14+<br>CD68+<br>CD163+<br>HLA-DR+<br>CD33+<br>MHC-II+	Macrophages	Macrophages can exist in several activation states including M1 and M2. M1 macrophages were associated with proinflammatory and antitumor effects whereas M2 macrophages were associated with anti-inflammatory and tolerogenic properties. CD68 alone can't differentiate between the two phenotypes, so additional antibodies could be added to the antibody panel to further study their prognostic value and reflect the findings on the immunoscore.	PMID: 29065107
CD11b+<br>CD14+<br>CD15+<br>CD33+<br>CD66b+	Myeloid-derived suppressor cells	They were shown to have immunosuppressive capabilities and were shown to support tumor invasion and metastasis. They can be added to the immunoscore to reflect their negative immune modulation nature.	PMID: 29348500
CD80+<br>CD86+<br>CD83+<br>CD11b+<br>CD11c+<br>CCR7+<br>CD14+<br>CD1a+	Dendritic cells	Given the different phenotypes that make up the dendritic cell population, they are associated with differential prognostic outcomes. CD103+ DCs and CD208+ DCs were associated with positive prognostic values. Thereby, it could be interesting to leverage multiplex panels of antibodies to decipher the differential prognostic values of dendritic cells and reflect that on the immunoscores.	PMID: 25446897
CD56+<br>CD94+	Natural killer cells	Prior reports showed that NK at some levels was associated with positive disease outcomes. However, if NK cells are present in high	PMID: 29018445

C122+<br>NKG2A+<br>NKG2D+<br>Eomes+		amounts, their profile changes, and they become associated with negative disease outcomes. It might be valuable to use image analysis to further investigate these thresholding effects and reflect that on the immunoscore	
CD19+<br>CD20+<br>CD21+<br>CD40+	B cells	Although B cells were generally linked to good disease outcomes, some studies reported a negative prognostic value of B cells. This can be attributed to the different subtypes and phenotypes of B cells. Multiplex imaging and image analysis could be used to decipher the B cells phenotypes and their differential prognostic values.	PMID: 28626234
BTLA+<br>CD3+<br>CD4+<br>CD40L+<br>CD57+<br>CD84+<br>CXCR4+<br>CXCR5+<br>ICOS+<br>CD10+<br>CD150+	T follicular helper cells	TFH was associated with positive disease outcomes and overall survival, so it could be a valuable addition to the immunoscore to further investigate its spatial prognostic value.	PMID: 29628290
CD3+<br>CD4+<br>CCR3+<br>CCR4+<br>CCR8+<br>CXCR4+<br>STAT5+<br>STAT6+	T helper type 2 (Th2) cells	Th2 cells show conflicting results in solid tumors, suggesting that there are several phenotypes of Th2 cells whereas each phenotype is associated with different prognostic values. Multiplex imaging technologies along with high-dimensional image analysis can be used to decipher Th2 phenotypes and their prognostic value.	PMID: 29628290
CCR4+<br>CCR6+<br>CD3+<br>CD4+<br>STAT3+<br>Batf+	T helper type 17 (Th17) cells	Th17 cells have differential prognostic values based on their location within patients' tumors. Intraepithelial Th17 cells were shown to have a positive prognostic value whereas stromal Th17 didn't maintain the same impact. Using multiplex imaging to identify Th17 along with spatial image analysis could yield a valuable spatially variant immunoscore.	PMID: 26719303
CCR1+<br>CCR5+<br>CD3+<br>CD4+<br>CXCR3+<br>STAT1+<br>STAT4+<br>T-bet+	T helper type 1 (Th1) cells	It is associated with a positive prognosis for several solid tumors but was associated with reduced survival for NSCLC.	PMID: 22419253

Supplementary Table 4: The clinical significance of the spatial infiltration patterns of immune cell types

	CD68-T	CD68-S	CD68-CD8	CD8-T	CD8-S	CD8-CD68
CH2_B7	0.0764	0.0393	0.0583	0.1844	0.0705	0.0361
CH2_C3	0.0806	0.0439	0.0863	0.2484	0.0954	0.0495

CH2_D1	0.1029	0.038	0.0936	0.1527	0.0901	0.057
CH2_D2	0.0501	0.0623	0.0563	0.1898	0.1259	0.0444
CH2_D5	0.1425	0.0238	0.0214	0.2699	0.032	0.0232
CH2_D9	0.1434	0.1298	0.0572	0.2064	0.1358	0.0427
CH2_F7	0.0606	0.0308	0.0512	0.1689	0.0789	0.0356
CH2_G3	0.0531	0.0693	0.0621	0.1981	0.1231	0.0397
CH2_H1	0.0842	0.0127	0.0147	0.1465	0.0189	0.0097
CH2_H2	0.0993	0.0679	0.0574	0.1777	0.069	0.062
CH2_H5	0.0424	0.0258	0.0158	0.1929	0.0221	0.0131
CH2_H9	0.0674	0.1256	0.0766	0.1392	0.1284	0.1005
CH1_B1	0.0383	0.0132	0.0013	0.0218	0.0022	0.0015
CH1_B3	0.0875	0.1076	0.0193	0.0519	0.0314	0.0264
CH1_B5	0.0944	0.0302	0.0116	0.0578	0.027	0.0122
CH1_C6	0.16	0.0834	0.088	0.0931	0.0662	0.1086
CH1_D7	0.1155	0.1501	0.0555	0.0698	0.0859	0.0877
CH1_D9	0.0493	0.0268	0.0119	0.0399	0.0253	0.014
CH1_E1	0.0868	0.1318	0.1807	0.123	0.1279	0.1446
CH1_B6	0.0496	0.0361	0.0275	0.0682	0.0526	0.0286
CH1_F3	0.0621	0.1777	0.1912	0.0907	0.1718	0.1823
CH1_F2	0.1008	0.0736	0.0632	0.0588	0.0632	0.07
CH1_G1	0.0336	0.0758	0.0113	0.0061	0.0602	0.0121
CH1_G5	0.0574	0.0453	0.0139	0.0647	0.0596	0.0117
CH1_E5	0.0338	0.0087	0.0028	0.0768	0.0073	0.0021
CH1_E7	0.0147	0.015	0.0042	0.0924	0.0155	0.0045

Supplementary Table 5. Interaction score between CD8+, CD68+, tumor, and stromal cells in both cohorts 1 and 2.

	CD68-T	CD68-S	CD68-CD8	CD8-T	CD8-S	CD8-CD68
CH2_B7	4.25	1.81	3	6.76	4.03	1.91
CH2_C3	4.54	2.08	4.48	9.18	5.5	2.65
CH2_D1	6.07	1.73	4.86	5.56	5.18	3.07
CH2_D2	2.44	3.17	2.9	6.96	7.29	2.37
CH2_D5	8.8	0.89	1.06	10	1.76	1.2
CH2_D9	8.86	7.17	2.94	7.59	7.88	2.28

CH2_F7	3.16	1.31	2.63	6.17	4.52	1.89
CH2_G3	2.64	3.59	3.2	7.28	7.13	2.11
CH2_H1	4.78	0.24	0.71	5.32	0.98	0.45
CH2_H2	5.82	3.5	2.95	6.5	3.94	3.35
CH2_H5	1.91	1.01	0.76	7.08	1.17	0.64
CH2_H9	3.63	6.92	3.97	5.05	7.44	5.48
CH1_B1	1.62	0.27	0	0.6	0	0
CH1_B3	5.01	5.85	0.95	1.74	1.72	1.38
CH1_B5	5.49	1.27	0.54	1.96	1.46	0.59
CH1_C6	10	4.42	4.57	3.3	3.77	5.92
CH1_D7	6.94	8.37	2.85	2.41	4.94	4.77
CH1_D9	2.38	1.07	0.56	1.28	1.36	0.69
CH1_E1	4.96	7.28	9.45	4.43	7.41	7.91
CH1_B6	2.4	1.62	1.38	2.35	2.97	1.5
CH1_F3	3.26	10	10	3.21	10	10
CH1_F2	5.93	3.84	3.26	2	3.6	3.79
CH1_G1	1.3	3.97	0.53	0	3.42	0.59
CH1_G5	2.94	2.17	0.66	2.22	3.38	0.56
CH1_E5	1.31	0	0.08	2.68	0.3	0.03
CH1_E7	0	0.37	0.15	3.27	0.78	0.17

Supplementary Table 6. Scaled interaction score between CD8 +, CD68 +, tumor and stroma cells in cohorts 1 and 2.

Technology	Multiplexing limits	Immunoscore	Reference
Hematoxylin and Eosin (H&E)	No	Assessing the infiltration of tumor-infiltrating lymphocytes in solid tumors including colorectal, breast, lung, prostate, etc concerning patients' survival.	DOI: 10.21873/anticanres.13041<br>DOI: 10.1016/j.celrep.2018.03.086
Immunohistochemistry (IHC)	Yes, 1-3	Assessing the infiltration of CD3+ and CD8+ within the tumor core and the invasive margin of several tumor types including colon, lung, pancreatic, etc. with survival, and disease recurrence.	DOI: 10.1007/s00262-020-02834-y<br>DOI: 10.1186/s40425-018-0488-6<br>DOI: 10.1038/s41379-019-0291-z

Immunofluorescence (IF)	Yes, cyclic experiment	Assessing the infiltration of several immune markers including CD3, CD4, CD8, CD20, FoxP3, PD-L1, etc within several tumor types including lung cancer, breast cancer, etc.	DOI: 10.1093/jnci/dju435 DOI: 10.1038/s41598-021-01116-6
Imaging mass cytometry (IMC)	Yes, simultaneous detection ~35 markers	Dissecting the immune microenvironment in diffuse large B cell lymphoma using a panel of different markers checkpoint markers (PD-1, PD-L1, TIM-3, VISTA, LAG-3), chemokines and their receptors (CXCR3, CCR4), immune markers (CD3, CD3, CD8, CD45RO, FoxP3, Granzyme B, etc)	DOI: 10.1101/2021.02.01.21250775

Supplementary Table 7. Comparing other imaging technologies with IMC for developing immunoscores for cancers.

**Patient ID Number**	**Race**	**Clinical Response**	**Age (years)**	**Number of prior treatment lines**	**Checkpoint Inhibitor**	**Chemotherapy partner**	**Overall survival (months)**
1	black	non-responder	46	3	pembrolizumab	Nab-paclitaxel	23
2	black	non-responder	45	5	atezolizumab	Nab-paclitaxel	18
3	black	non-responder	32	0	pembrolizumab	Gemcitabine/ Carboplatin	5
4	black	responder	66	0	atezolizumab	Nab-paclitaxel	21
5	white	responder	67	0	atezolizumab	Nab-paclitaxel	NA
6	black	responder	39	2	pembrolizumab	Gemcitabine/ Carboplatin	NA

Supplementary Table 8. Breast cancer patient characteristics.

**Patient barcode**	**Cancer subtype**	**Tumor Status**	**Vital Status**	**Last contact day to**	**Death day to**	**Therapy type**	**Treatment best respond**
TCGA-05-4402		TUMOR FREE	Dead	[Not Available]	244	Chemotherapy	Responder
TCGA-44-7660		TUMOR FREE	Alive	162	[Not Applicable]	Vaccine	Non Responder

TCGA-44-A47F		TUMOR FREE	Alive	246	[Not Applicable]	Chemotherapy	Responder
TCGA-49-AAR2		TUMOR FREE	Alive	1945	[Not Applicable]	Chemotherapy	Responder
TCGA-50-5055		WITH TUMOR	Alive	785	[Not Applicable]	Chemotherapy	Non Responder
TCGA-50-5068		WITH TUMOR	Dead	1499	1499	Chemotherapy	Non Responder
TCGA-53-A4EZ		TUMOR FREE	Alive	280	[Not Applicable]	Chemotherapy	Responder
TCGA-55-1596		TUMOR FREE	Alive	1375	[Not Applicable]	Chemotherapy	Responder
TCGA-55-5899		[Not Available]	Alive	87	[Not Applicable]	Chemotherapy	Responder
TCGA-55-6712		WITH TUMOR	Alive	24	[Not Applicable]	Chemotherapy	Non Responder
TCGA-55-6979		WITH TUMOR	Dead	[Not Available]	237	Chemotherapy	Non Responder
TCGA-55-6982		WITH TUMOR	Dead	[Not Available]	995	Chemotherapy	Non Responder
TCGA-55-6983		TUMOR FREE	Alive	1826	[Not Applicable]	Chemotherapy	Responder
TCGA-55-7815		TUMOR FREE	Alive	54	[Not Applicable]	Chemotherapy	Non Responder
TCGA-55-7914		WITH TUMOR	Alive	3	[Not Applicable]	Chemotherapy	Non Responder
TCGA-55-8513		TUMOR FREE	Alive	146	[Not Applicable]	Chemotherapy	Non Responder
TCGA-55-8615		TUMOR FREE	Alive	15	[Not Applicable]	Chemotherapy	Non Responder
TCGA-55-A48Y		TUMOR FREE	Alive	42	[Not Applicable]	Chemotherapy	Responder
TCGA-62-A471		TUMOR FREE	Alive	883	[Not Applicable]	Chemotherapy	Responder
TCGA-64-5778		TUMOR FREE	Alive	926	[Not Applicable]	Chemotherapy	Non Responder
TCGA-69-7974		WITH TUMOR	Alive	184	[Not Applicable]	Chemotherapy	Non Responder
TCGA-71-6725		TUMOR FREE	Alive	61	[Not Applicable]	Chemotherapy	Non Responder
TCGA-73-4675		TUMOR FREE	Alive	40	[Not Applicable]	Chemotherapy	Responder

TCGA-73-7498		TUMOR FREE	Alive	621	[Not Applicable]	Chemotherapy	Responder
TCGA-86-8279		TUMOR FREE	Alive	21	[Not Applicable]	Chemotherapy	Responder
TCGA-86-8674		TUMOR FREE	Alive	0	[Not Applicable]	Chemotherapy	Non Responder

Supplementary Table 9. Cohort 3 of 26 TCGA lung tumor patients with clinical information.
