# Supplementary material for: Spatially variant immune infiltration scoring in human cancer tissues
Source: NPJ Precis Oncol. 2022 Sep 1;6:60. doi: 10.1038/s41698-022-00305-4 (PMC9437065; doi:10.1038/s41698-022-00305-4)
Supplement: Supplementary file 2 — REPORTING SUMMARY [file 41698_2022_305_MOESM2_ESM.pdf]

nature portfolio

Corresponding author(s): Ahmet F. Coskun, Ph.D.

Last updated by author(s): Jul 7, 2022

Reporting Summary

Nature Portfolio wishes to improve the reproducibility of the work that we publish. This form provides structure for consistency and transparency in reporting. For further information on Nature Portfolio policies, see our Editorial Policies and the Editorial Policy Checklist.

Statistics

For all statistical analyses, confirm that the following items are present in the figure legend, table legend, main text, or Methods section.

n/a	Confirmed	
☐	☒	The exact sample size (*n*) for each experimental group/condition, given as a discrete number and unit of measurement
☐	☒	A statement on whether measurements were taken from distinct samples or whether the same sample was measured repeatedly
☐	☒	The statistical test(s) used AND whether they are one- or two-sided<br>*Only common tests should be described solely by name; describe more complex techniques in the Methods section.*
☐	☒	A description of all covariates tested
☐	☒	A description of any assumptions or corrections, such as tests of normality and adjustment for multiple comparisons
☐	☒	A full description of the statistical parameters including central tendency (e.g. means) or other basic estimates (e.g. regression coefficient) AND variation (e.g. standard deviation) or associated estimates of uncertainty (e.g. confidence intervals)
☐	☒	For null hypothesis testing, the test statistic (e.g. *F*, *t*, *r*) with confidence intervals, effect sizes, degrees of freedom and *P* value noted<br>*Give P values as exact values whenever suitable.*
☐	☒	For Bayesian analysis, information on the choice of priors and Markov chain Monte Carlo settings
☐	☒	For hierarchical and complex designs, identification of the appropriate level for tests and full reporting of outcomes
☐	☒	Estimates of effect sizes (e.g. Cohen's *d*, Pearson's *r*), indicating how they were calculated

Our web collection on statistics for biologists contains articles on many of the points above.

Software and code

Policy information about availability of computer code

Data collection	Acquired original data by Imaging Mass Cytometry Technology through the Hyperion Imaging System.
Data analysis	1) Open source code in Jupyter Notebook and complementary analysis using 2) ImageJ, 3) CellProfiler, and 4) HistoCAT.

For manuscripts utilizing custom algorithms or software that are central to the research but not yet described in published literature, software must be made available to editors and reviewers. We strongly encourage code deposition in a community repository (e.g. GitHub). See the Nature Portfolio guidelines for submitting code & software for further information.

Data

Policy information about availability of data

All manuscripts must include a data availability statement. This statement should provide the following information, where applicable:

- Accession codes, unique identifiers, or web links for publicly available datasets
- A description of any restrictions on data availability
- For clinical datasets or third party data, please ensure that the statement adheres to our policy

SpatialViz analysis codes and example datasets are available at: https://github.com/coskunlab/SpatialVizScore

Human research participants

Policy information about studies involving human research participants and Sex and Gender in Research.

Reporting on sex and gender	Patients tissues were from both male (n=18) and female donors (n=8).
Population characteristics	All patients had lung cancer from varying stages (I-IIIB) and grades (1-3).
Recruitment	The tissues samples were are de-identified.
Ethics oversight	The tissues samples were obtained from Biomax, US and all human tissues are collected under HIPAA approved protocols.

Note that full information on the approval of the study protocol must also be provided in the manuscript.

Field-specific reporting

Please select the one below that is the best fit for your research. If you are not sure, read the appropriate sections before making your selection.

☒ Life sciences ☐ Behavioural & social sciences ☐ Ecological, evolutionary & environmental sciences

For a reference copy of the document with all sections, see nature.com/documents/nr-reporting-summary-flat.pdf

Life sciences study design

All studies must disclose on these points even when the disclosure is negative.

Sample size	26 patient tissues.
Data exclusions	No data was excluded.
Replication	At least duplicate images of the same health and disease conditions.
Randomization	Defined spatially organized data based on tissue regions.
Blinding	Direct tissue data with visuals, no blinding was needed.

Reporting for specific materials, systems and methods

We require information from authors about some types of materials, experimental systems and methods used in many studies. Here, indicate whether each material, system or method listed is relevant to your study. If you are not sure if a list item applies to your research, read the appropriate section before selecting a response.

Materials & experimental systems

n/a	Involved in the study	
☐	☒	Antibodies
☒	☐	Eukaryotic cell lines
☒	☐	Palaeontology and archaeology
☒	☐	Animals and other organisms
☐	☒	Clinical data
☒	☐	Dual use research of concern

Methods

n/a	Involved in the study	
☒	☐	ChIP-seq
☒	☐	Flow cytometry
☒	☐	MRI-based neuroimaging

Antibodies

Antibodies used	Supplementary Table 1 shows the details of these antibodies, CD20, CD3, CD4, CD45RO, CD68, CD8a, FoxP3, Pan-Keratin, Granzyme B, Ki-67, PD-1, PD-L1, Smooth Muscle Actin (SMA), Collagen Type 1, E-cadherin, Histone 3, Vimentin, CD44, MHC-II, H3K9me3, HLA-DR, CD11c, CD163, CD206, CD95, CD103, and TCF1.
Validation	Antibodies were mostly obtained from Fluidigm as a validated immune panel. Additional validations were performed by immunohistochemistry (IHC) in tonsil formalin-fixed, paraffin-embedded (FFPE) sections.

Clinical data

Policy information about clinical studies

All manuscripts should comply with the ICMJE guidelines for publication of clinical research and a completed CONSORT checklist must be included with all submissions.

Clinical trial registration	*Provide the trial registration number from ClinicalTrials.gov or an equivalent agency.*
Study protocol	*Note where the full trial protocol can be accessed OR if not available, explain why.*
Data collection	*Describe the settings and locales of data collection, noting the time periods of recruitment and data collection.*
Outcomes	*Describe how you pre-defined primary and secondary outcome measures and how you assessed these measures.*
